# First Phase 1 Double-Blind, Placebo-Controlled, Randomized Rectal Microbicide Trial Using UC781 Gel with a Novel Index of *Ex Vivo* Efficacy

**DOI:** 10.1371/journal.pone.0023243

**Published:** 2011-09-28

**Authors:** Peter A. Anton, Terry Saunders, Julie Elliott, Elena Khanukhova, Robert Dennis, Amy Adler, Galen Cortina, Karen Tanner, John Boscardin, William G. Cumberland, Ying Zhou, Ana Ventuneac, Alex Carballo-Diéguez, Lorna Rabe, Timothy McCormick, Henry Gabelnick, Christine Mauck, Ian McGowan

**Affiliations:** 1 Center for HIV Prevention Research, UCLA AIDS Institute, Department of Medicine, David Geffen School of Medicine at the University of California Los Angeles, Los Angeles, California, United States of America; 2 Department of Biostatistics, University of California Los Angeles School of Public Health, Los Angeles, California, United States of America; 3 Magee-Womens Research Institute, University of Pittsburgh Medical School, Pittsburgh, Pennsylvania, United States of America; 4 Department of Psychiatry, Columbia University, New York, New York, United States of America; 5 CONRAD, Arlington, Virginia, United States of America; University of Cape Town, South Africa

## Abstract

**Objectives:**

Successful control of the HIV/AIDS pandemic requires reduction of HIV-1 transmission at sexually-exposed mucosae. No prevention studies of the higher-risk rectal compartment exist. We report the first-in-field Phase 1 trial of a rectally-applied, vaginally-formulated microbicide gel with the RT-inhibitor UC781 measuring clinical and mucosal safety, acceptability and plasma drug levels. A first-in-Phase 1 assessment of preliminary pharmacodynamics was included by measuring changes in *ex vivo* HIV-1 suppression in rectal biopsy tissue after exposure to product *in vivo*.

**Methods:**

HIV-1 seronegative, sexually-abstinent men and women (N = 36) were randomized in a double-blind, placebo-controlled trial comparing UC781 gel at two concentrations (0.1%, 0.25%) with placebo gel (1∶1∶1). Baseline, single-dose exposure and a separate, 7-day at-home dosing were assessed. Safety and acceptability were primary endpoints. Changes in colorectal mucosal markers and UC781 plasma drug levels were secondary endpoints; *ex vivo* biopsy infectibility was an ancillary endpoint.

**Results:**

All 36 subjects enrolled completed the 7–14 week trial (100% retention) including 3 flexible sigmoidoscopies, each with 28 biopsies (14 at 10 cm; 14 at 30 cm). There were 81 Grade 1 adverse events (AEs) and 8 Grade 2; no Grade 3, 4 or procedure-related AEs were reported. Acceptability was high, including likelihood of future use. No changes in mucosal immunoinflammatory markers were identified. Plasma levels of UC781 were not detected. *Ex vivo* infection of biopsies using two titers of HIV-1_BaL_ showed marked suppression of p24 in tissues exposed *in vivo* to 0.25% UC781; strong trends of suppression were seen with the lower 0.1% UC781 concentration.

**Conclusions:**

Single and 7-day topical rectal exposure to both concentrations of UC781 were safe with no significant AEs, high acceptability, no detected plasma drug levels and no significant mucosal changes. *Ex vivo* biopsy infections demonstrated marked suppression of HIV infectibility, identifying a potential early biomarker of efficacy. (Registered at ClinicalTrials.gov; #NCT00408538)

## Introduction

Efforts to reduce the sexual transmission of HIV-1 are pivotal to controlling the AIDS pandemic. Sustained plasma suppression reduces transmission but trials of HIV-specific vaccines and topical microbicides have been challenging in heterosexual couples and men who have sex with men (MSM) populations, especially given the still-poorly understood immune responses at the sexually-exposed mucosal portals of virus entry [1]–[9]. The recent results from both the Phase IIb CAPRISA 004 Trial of vaginally-applied 1% tenofovir gel and the Phase III iPrEx Trial of oral Truvada tablets (a co-formulation of tenofovir disoproxil fumarate and emtricitabine) have been exciting, first-time achievements in HIV prevention [10], [11].

Microbicides have been advanced as a topical mode of reducing HIV-1 transmission “per sexual act.” While discussed as a topical version of PrEP [12], use of topical microbicides is intended to provide a safe, acceptable, affordable form of protection from HIV-1 transmission, providing receptive partners (women and men) with options, especially when condom use is non-negotiable [13]. The spermicidal and contraceptive vaginal agent, nonoxynol-9 (N9) was demonstrated, post-approval, to create an increased risk for HIV-1 acquisition with frequent vaginal use. Significant epithelial sloughing was seen when applied rectally. This experience identified newer safety parameters to consider when evaluating microbicidal agents [14]–[16]. Until recently, clinical trial efforts have focused on vaginal transmission with mostly disappointing results [17]–[21]. A first-in-field success, CAPRISA 004 utilized a reverse-transcriptase inhibitor (1% tenofovir) gel applied 12 hours before and after vaginal intercourse. The study demonstrated a >50% reduction in HIV-1 transmission in those women using the gel for >80% of episodes [10], [11]. Equally exciting, in different risk groups, was the recent iPrEx trial demonstration of 44% reduction of HIV-1 transmission in ∼2500 higher-risk MSM at 11 study sites worldwide [11]. As in the CAPRISA trial, when the inherently difficult issue of adherence is teased apart, sub-analyses suggest the prevention rate may be 50% or higher. Both studies successfully demonstrated proof-of-concept for topical microbicides.

Rectal transmission of HIV-1 is thought to be 20–200-times more likely per sexual act than vaginal transmission, perhaps related to the single-cell epithelial lining and extensive, activated resident immunocyte populations [1]–[7], [22], [23]. Receptive anal intercourse (RAI) is highly prevalent among MSM and also in heterosexual sexual partnerships [24]–[30]. It is anticipated that when the mucosa is co-infected (such as with HSV) or significant trauma, the rate of rectal transmission per sex act would markedly increase [31]–[34].

This report describes the first IND-supported Phase 1 safety trial of two concentrations of UC781 (0.25% and 0.1%) as a rectal microbicide. UC781 is a potent non-nucleoside reverse transcriptase inhibitor (NNRTI) which binds tightly to HIV-1 RT [35]–[40], has activity against a wide range of subtype HIV-1 isolates and is poorly absorbed from mucosal surfaces with systemic limited bioavailability. UC781 displays *in vitro* nanomolar range EC_50_ activity against wild type HIV-1 virus and little to no cytotoxic effect on cell lines and primary cells. In pre-clinical studies of human cervical and colorectal explants pre-incubated with UC781, R5 HIV_BaL_ was markedly suppressed, decreasing the infection in migrating lymphoid cells [41], [42]. UC781 added *in vitro* showed 100% inhibition of HIV_BaL_ at 3.3 µg/ml and 90% inhibition at 0.33 µg/ml. These infectious doses are thought to be far in excess of ejaculate concentrations [43]–[45]. For comparison, the delivered doses (empirically assuming a 10× dilution by rectal fluids) in this trial for the 0.1% gel was a dose of 3.5 mg in 3.5 ml (1000 µg/ml) and for the 0.25% gel, a dose of 8.75 mg in 3.5 ml (2500 mg/ml).

Two concentrations of UC781 gel (0.10% and 0.25%) formulated for topical vaginal application and demonstrating safety in early dose-ranging vaginal safety studies [46]–[48] were applied rectally in this study. The product was delivered using the same applicator design as used in vaginal microbicide trials. The novelty of this “first in field” study was facilitated by the HPTN-056 study which established normative ranges and inter-subject variabilities in a host of recently developed mucosal indices to assess potential mucosal injury [49].

An innovative 2-stage trial design was used consisting of an initial, single rectal application of either of the two concentrations of UC781 or hydroxyethyl cellulose (HEC) placebo gel followed several weeks later by 7 once-daily, self-administered rectal applications. We have previously reported the acceptability evaluations carried out in this trial [50]. Here we report the clinical safety of vaginally-formulated UC781, plasma drug concentrations, mucosal safety and the use of novel *ex vivo* biopsy HIV-1 infection challenges of *in vivo* exposed tissue samples, Both concentrations of UC781 were shown to be clinically safe by all indices used with few Grade 2 and no Grade 3, 4 or procedure-related AEs. Plasma levels of UC781 were not seen and no significant mucosal abnormalities were detected. Remarkably, the *ex vivo* HIV-1 infection of tissue biopsies showed marked suppression with UC781 0.25%.

## Methods

The study was designed by the investigators with collaborative input from CONRAD and the NIAID/DAIDS/Prevention Sciences Integrated Preclinical-Clinical Program (IPCP), as stipulated in the award notice and reviewed by the U.S. Food and Drug Administration (FDA). The study was approved by the UCLA Office of the Human Research Protection Program Institutional Review Board (UCLA IRB) and all subjects provided written informed consent. The trial is registered at ClinicalTrials.gov, number NCT00408538 and is in compliance with the CONSORT 2010 recommendations for reporting of trial results (www.consort-statement.org) [51], [52].

### Study population

HIV-1 seronegative men and women between the ages of 18 and 64 (inclusive) with a history of consensual receptive anal intercourse (RAI) at least once in lifetime were eligible for screening. This was included to identify individuals with some familiarity of sexual or product insertion per rectum. Lower risk of HIV infection was supported by HIV screening tests for inclusion, the additional inclusion criteria of willingness to be sexually abstinent regarding rectal sex or any other rectal insertions one week prior to treatment and one week before and following each flexible sigmoidoscopy. Ongoing monitoring of plasma HIV RNA further supported this. Exclusion criteria included HIV infection, known inflammatory bowel disease or any other chronic gastrointestinal disorder and/or history of significant gastrointestinal bleeding as well as any allergies to methlyparaben, propylparaben or sorbic acid.

### Study Products

Two concentrations of UC781 (0.1% or 0.25% w/w), Carbomer 974P, methylcellulose, glycerin, purified water and common preservatives (methlyparaben and propylparaben), adjusted to pH 5.2, were prepared in vaginally-formulated, aqueous gel formulations. The single dose, prefilled vaginal applicators (the same type of applicators as used in UC781 vaginal microbicide trials) were packaged, overwrapped and shipped directly to the study site pharmacy. Each applicator contained either a dose of 3.5 mg in 3.5 ml (1000 µg/ml) for the 0.1% gel, a dose of 8.75 mg in 3.5 ml (2500 µg/ml) for the 0.25% gel, or 3.5 g in 3.5 ml of aqueous HEC (hydroxyethyl cellulose) gel, adjusted to pH 4.4, as the placebo gel [53].

### Study Design

**Figure 1:**
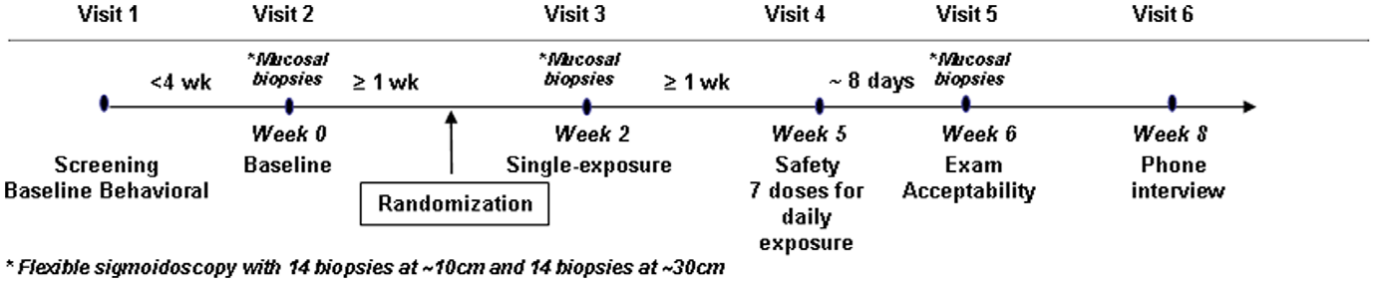
Study Flow Diagram.

This was a single site, blinded, multi-arm, two-treatment stage Phase 1 trial using two established UC781 drug concentrations and HEC placebo in 36 subjects in a 1∶1∶1 randomization (Figure 1). The protocol for this trial and supporting CONSORT checklist are available as supporting information; see Checklist S1 and Protocol S1. The first stage was a randomized single exposure, directly applied by clinical personnel, with the subject remaining supine for the 30 minute period until sample collection. The second stage, assessed separately, was a randomized (same groups), 7-day cumulative exposure delivered by participants at home. Participants were given a Product Use Log for the 7-day use and were called daily to encourage use and recording of symptoms/time of use.

The primary objectives of the study were to evaluate the safety and acceptability of 0.1% and 0.25% UC781 vaginal microbicide gel versus placebo when applied rectally.

The primary endpoints were (i) the frequency of ≥Grade 2 adverse events and (ii) extensive acceptability evaluations (reported elsewhere [50]. The primary tool for safety assessments and grading adverse events was the DAIDS AE Grading Table Version 1.0, December 2004, Addenda 1 (female genital) and 3 (rectal-specific) Grading Tables for Use in Microbicide Studies (http://rsc.tech-res.com/safetyandpharmacovigilance). AE gradings are defined as symptoms causing no or minimal (Grade1), causing greater than minimal (Grade 2) interference with or causing inability to perform (Grade 3) usual social and functional activities. Physical signs and laboratory values are graded similarly to the ones listed in the main DAIDS AE Grading Table. Details of demographics and adverse events are provided in Table S1 and Table S2.

One secondary objective of the study was to determine whether use of study products was associated with rectal mucosal damage. Secondary endpoints included rectal epithelial sloughing, histopathology, rectal microflora, fecal calprotectin, and a group of “mucosal immunotoxicity indices,” which included flow cytometric characterization of isolated mucosal mononuclear cells (MMCs), secreted mucosal immunoglobulins, secreted rectal fluid cytokines, mucosal tissue cytokine mRNA and susceptibility to HIV infection of rectal tissue biopsies.

An additional secondary objective was to determine the pharmacokinetics (PK) of UC781 vaginal microbicide gel administered rectally in a subset of participants. The endpoint was UC781 blood levels as an indicator of absorption of drug from the GI tract.

Enrollment was protocol-defined as having met initial, screening criteria at Visit 1 (eligibility requirements, consent signing, STI evaluations and baseline acceptability questionnaires answered via CASI) and having been randomized post Visit 2. During Visit 2, baseline sample collections and flexible sigmoidoscopy for each subject's baseline mucosal biopsy infectibility, PK as well as additional acceptability assessments occurred. PK assessments for UC781 were measured in a subset of enrolled, randomized participants. All subject visits were at the UCLA Digestive Diseases Clinic and/or UCLA Outpatient Endoscopy Suite.

Following enrollment, 36 subjects were randomized, in a double-blind fashion, to one of the three treatment arms. Randomization was performed in blocks of six, with each block containing two assignment codes for each of the three treatment groups. A subset of 9 subjects (3 from each group) also participated in a PK sub-study, which included 6 timepoints (pre-dosing, 0.25 hr, 2 hr, 4 hr, and 24 hr after the single dose, and 1 day post-daily dosing for 9 of the 36 study participants (3 participants in each study group: placebo, 0.25% UC781 and 0.1% UC781) were randomized for UC781 plasma levels for 24-hour PK (baseline/pre-dosing, 30 minute, 2, 4 and 24 hours following single exposure) and also single cumulative level following 7-day dosing (total of 54 samples). Randomization codes were generated by the study biostatistician using computer-generated random numbers. Once generated, the UCLA investigational pharmacy held primary responsibility for dispensing drug, randomizing subjects and maintaining the blind.

Each participant had three flexible sigmoidoscopies for mucosal biopsies and other sample collections: baseline (Visit 2), 30 minutes post single topical exposure (Visit 3) and the morning following completion of 7 daily at-home topical exposures (Visit 5). Rectosigmoid colonic biopsies (referred to hereafter as “colonic biopsies”) were collected endoscopically using large cup biopsy forceps via a flexible sigmoidoscope as previously described [49], [54]. The first set of colorectal biopsies were then acquired at 10 cm from the anal verge and then at 30 cm (14 biopsies acquired at each site; 28 total biopsies per visit).

Each study visit to the endoscopy suite for flexible sigmoidoscopy had an established sequence to minimize confounding of endpoint results. Phlebotomy for safety laboratories as well as UC781 PK plasma samples occurred first (pre-exposure at Visit3 and then at 30 minutes, 2 hr, 4 hr and 24 hr); rectal sponges (ULTRACELL® Aspen Surgical #40415, Caledonia, Michigan) then were applied via plastic anoscope as previously described [55] for collection of mucosal secreted immunoglobulins and cytokines as well as rectal microflora. A Normosol® preparatory enema was administered with participants evacuating enema contents into a toilet-seat plastic collection unit for stool sample collection for calprotectin (same-day shipment to Genova Diagnostics, Asheville, NC). Microflora samples were shipped same day for characterization to Dr. Sharon Hillier's Laboratory at Magee-Womens Research Institute, Pittsburgh, PA. Participants were then taken to the endoscopy room. Following digital examination, the flexible sigmoidoscope was introduced to a distance of 5 cm in the rectal vault where 50 cc of Normosol® solution was slowly infused. Following 30 seconds, at least 25–30 ml of lavage fluid was aspirated through the endoscope to a collection trap vial to assess epithelial sloughing. The endoscope was then advanced to collect biopsies as described above. Endoscopic pictures/grading were not recorded due to the known variability of the colorectal lining in health.

### Behavioral and acceptability measures (primary endpoint)

All participants completed the Baseline Behavioral Questionnaire (BBQ) prior to Visit 3. Product Acceptability Questionnaires (PAQs) were completed after the 7-day exposure. The PAQ included both close-ended and open-ended questions, assessed using a password-protected Web-based survey, enabling direct data entry. An extensive, final 1-hour, in-depth acceptability interview was conducted. Detailed methods and results of this portion of this trial have been published [50].

### Indicators of rectal mucosal damage

A diverse panel of mucosal assessments to evaluate potential injury or significant alteration from baseline immune parameters was used to detect changes post-gel exposure compared to baseline findings within individual subjects and between study groups [49]. The broad profile of mucosal markers of inflammation/injury is included based on several years of development, optimization and determination of *in vivo* stability, reproducibility and variability. The clinical relevance of two-fold or more changes in any of these parameters is not known but the absence of any changes provides a degree of confidence of non-injury [49]. The assays used to monitor mucosal injury, activation and/or inflammation are described below. Following the methods is a listing of which samples were collected at which visits.

### Fecal calprotectin

During enhanced mucosal inflammation, intestinal granulocytes, containing large amounts of the cytoplasmic protein calprotectin, migrate through the mucosal epithelia and granulocyte-derived calprotectin can be found in feces, providing a useful indirect index of mucosal inflammation [56], [57]. Fecal calprotectin levels correlate well with disease activity in Crohn's disease and ulcerative colitis [58], [59]and were utilized here to indirectly detect acute inflammation. Stool was collected using a kit with mailing materials (Genova Diagnostics, Asheville, NC). A 20 ml stool sample was collected from participant's enema evacuations and mailed same day. Reported results from the company have a range with <50 µg/ml = normal; greater than 50 µg/ml to 500 µg/ml equating to varying levels of inflammation. Fecal calprotectin assays have a sensitivity of 96% in discriminating between healthy controls (2 mg/l; 95% CI 2–3 mg/l) and subjects with active inflammatory bowel disease (91 mg/l; 95% CI 59–105 mg/1) [56], [57].

### Rectal secretions of immunoglobulins and cytokines collected by sponges

Rectal secretions were collected using 4 cellulose sponges (ULTRACELL®, Aspen Surgical Caledonia, Michigan) for secreted immunoglobulins (2 dedicated sponges) and secreted cytokines (2 dedicated sponges) per collection event, as previously reported [49], [60]. Sterile sponges were pre-moistened with 50 µl of phosphate buffered saline (Gibco BRL, Gaithersburg, MD) and attached to an adapted 2 ml plastic transfer pipette (Fisher Scientific, Pittsburgh, PA). Two pipettes with attached sponges were introduced into the rectum via the anoscope and held against the rectal mucosa under direct vision for 5 minutes, then placed on ice until freezing at −80° for batch processing. Samples with visible blood were discarded. Sponge tips were transferred to a 2 ml Spin-X column (Corning, Corning, NY), from which the acetate membrane had been removed. This was repeated for the second set of two sponges. Absorbed rectal secretions were eluted twice with a total volume of 250 µl of cold elution buffer (PBS containing 0.25% BSA (Sigma Chemicals, St Louis, MO), 1% Igepal (Sigma Chemicals, St Louis, MO) and 1× protease inhibitor cocktail (Sigma Chemicals, St Louis, MO) from the sponges by centrifugation (10,000 rpm, 30 minutes at 4 degrees). The recovered eluate was transferred to a pre-weighed 1.5 ml Eppendorf tube (Fisher Scientific, Pittsburgh, PA) and re-weighed. The recovered volume of secretion was calculated by subtracting the recovered volume from that recovered from control sponges that were run in parallel. Duplicate samples were pooled, (usually yielding 400 µl), frozen and retrieved in batches for further analysis.

Total IgG and total IgA were quantified (in duplicate) in the eluted rectal secretions by ELISA [54] and results expressed as ng/ml. Values in ng/ml were extrapolated from the relevant standard curves and means calculated for each sample. The Coomassie Dry™ Blue Protein Assay (Pierce, Rockford, IL) was used to extrapolate the quantity of total protein expressed as ng/ml [49]. Cytokine samples were quantified (pg/ml) using 50 µl in a Lincoplex® Human cytokine/chemokine multiplex immunoassay kit (used in BioRad Luminex® 100 System multiplexing array instrument). Seven secreted cytokine proteins were quantified in pg/ml (RANTES, MIP-1α, TNF-α, IFNγ, IL-12 (p-40), IL-6, IL-1β).

### Microflora

Rectal specimens obtained per rectum with a sterile cotton swab were placed in an anaerobic transport tube (Port-a-Cul; Becton-Dickinson Corp., Cockeysville, MD) and shipped by overnight mail to Dr. Sharon Hillier's Laboratory at Magee-Womens Research Institute, Pittsburgh, PA. The swab was removed, placed into 0.9 ml of buffered salt solution and vortexed to release fluid and processed as previously reported [7]. Plates were incubated in an anaerobic chamber for 72–96 hours for detection of anaerobes; agar plates for aerobic bacteria were evaluated after 48 hours of incubation at 37°C in 6% CO_2_. Given the enormous variety of normally-present bacteria in the colon and the unknown variations related to diet and time, the bacterial groups selected for monitoring before and after product exposure was based on a FDA defined and accepted panel. These microflora are identified in Table 1. Changes in bacterial frequencies and concentrations within exposed individuals as well as between study groups were examined between baseline visit and following the 7-day exposure (which included any changes occurring after the single dose exposure). Results are quantified by colonization growth on a scale from 0–4 as follows: 0 = no growth; 1 = 10^3^ cfu/ml; 2 = 10^5^ cfu/ml; 3 = 10^6^ cfu/ml 4 = 10^7^ cfu/ml. Raw means and SDs were computed by time (before or/after gel use), using McNemar's test for evaluating paired changes in colonization status [61], [62].

**Table 1:** Changes in pre-defined rectal bacteria populations following cumulative exposure to 0.1% or 0.25% UC781 topical gel or HEC placebo compared to baseline.

	The percentage of each trial groups' participants with bacteria present at baseline is listed followed by the percentage of participants with bacterial presence following cumulative single/7-day exposures, with p values listed.								
	0.1% UC781 (n = 12)			0.25% UC781 (n = 12)			HEC (n = 12)		
Bacteria	Baseline	Post-exposure	p value	Baseline	Post-exposure	p Value	Baseline	Post-exposure	p Value
*Lactobacillus (H_2_O_2_-producing)*	33%	50%	0.68	33%	25%	1	24%	50%	0.68
*Lactobacillus (H_2_O_2_-nonproducing)*	17%	17%	1	17%	0%	0.48	17%	25%	0.5
*Gardnerella vaginalis*	0	0		25%	25%	1	0%	8%	0.3
*Escherichia coli*	83%	83%	1	92%	75%	0.59	92%	92%	1
Other gram-negative rods	17%	8%	1	25%	25%	1	25%	8%	0.59
Anaerobic gram-positive cocci	92%	92%	1	100%	100%	1	83%	83%	1
Anaerobic gram-positive rods (Clostridium)	67%	83%	0.64	83%	67%	0.64	92%	75%	0.59
Anaerobic gram-positive rods (other)	83%	67%	0.64	67%	92%	0.32	67%	100%	0.09
Anaerobic gram-negative rods	100%	100%	1	100%	100%	1	100%	92%	1
Black-pigmented anaerobic gram-negative rods	58%	83%	0.37	92%	83%	1	100%	83%	0.47

**NB:** No differences between the HEC and the UC781 exposed groups were seen. No differences in female to male cultures were seen.

### Epithelial sloughing

Collected lavage fluid (>25 ml) was transported on ice to the laboratory. Using a protocol adapted from Patton and Phillips [14], [63], the fresh lavage samples were placed in a 100×15 mm Petri dish, on the platform of a Leica EZ4 inverted microscope with attached CANON Powershot A630 camera, to count epithelial sheets. The sheets were measured in quadrants using a permanent ruler, affixed to the platform, under the Petri dish, as previously reported [14], [63]. Positive findings were defined as a clear, spongy, elastic piece of cellular material (with nucleus) at least 2 mm in size. As epithelial sloughing does not have an absolute, quantifiable threshold, the previously used scoring system of 0–4 was used. Each of four Petri-dish quadrants was scored as either 0 or 1, (absence or presence of epithelial sheets) for a total score from 0–4.

### Histology

Histopathological scoring of inflammation was carried out on oriented, formalin-fixed paraffin-embedded, hematoxylin- and eosin-stained tissue biopsies. Colonic mucosal biopsy samples were obtained at both 10 cm and 30 cm (measured by endoscopic markings) from the dentate line. A pathologist with specialty training in gastrointestinal pathology, blinded to sample group, performed the batched, qualitative (scale: 0–5) histological assessments. A validated, qualitative scoring scale of chronic active inflammation used in ulcerative colitis trials previously adapted and reported for baseline mucosal indices in microbicidal studies [49] was used.

### Isolation of mucosal mononuclear cells and flow cytometry

MMCs were isolated from intestinal biopsies obtained at both 10 cm and 30 cm levels using enzymatic digestion and staining, as previously published [54]. Biopsies were incubated in 20–25 ml RPMI/7.5% fetal calf serum containing 0.5-mg/ml collagenase type II (Sigma-Aldrich, St. Louis, MO) for 30 minutes in a 37°C water bath, with intermittent shaking. The entire suspension was then passed (up to three separate times) through a sterile plastic strainer (BD Falcon 2350, Bedford MA) to remove free cells and concentrate the remaining tissue fragments in a fresh 50 ml conical tube. Free cells were immediately washed twice in medium to remove excess collagenase, prior to being resuspended in 500 µl–1000 µl of media and set aside on ice. Monoclonal antibodies used included anti-CD4 and HLA-DR-fluorescein isothiocyanate (FITC), CD38, CXCR4 and CCR5–phycoerythrin (PE), CD4 and CD45-peridin chlorophyll protein (PER-cp), CCR5 and CXCR4-allophycocyanin (APC). All monoclonal antibodies were supplied by BD Immunocytometry Systems (BDIS), Mountain View, CA. Analysis was carried out on a FACSCalibur® flow cytometry (BDIS, Mountain View, CA) with analysis using CellQuestPro® software (BDIS, Mountain View, CA); quadrant settings were determined for well-defined populations, such as T-cell subsets, as previously described [54]. For those populations that were less well defined, such as CD38 and co-receptors, historical quadrant settings from prior studies were used [49]. Percentage values for the stained subsets were recorded.

Mucosal cytokine mRNA. Cytokine mRNA for IFNγ was measured in RNA extracted from 3 pooled endoscopic biopsies, snap-frozen in liquid nitrogen, from both the 10 cm and 30 cm level, using a previously described technique [23], [49]. Briefly, primers optimized for qRT-PCR were designed using the cDNA sequences of the gene of interest from the genebank database in conjunction with Primer Express software (Applied Biosystems, Foster City, CA). All assays were performed in triplicate and a mean calculated from the three measurements obtained. Results are reported as copies of cytokine mRNA standardized per 10^6^ copies of β-actin.

### Colonic explants for HIV-1 infectivity

Endoscopic biopsies from the 10 cm and the 30 cm site were collected in 50 ml RPMI (with 1.125 µg/ml of Fungizone and 50 mg/ml of Zosyn, transported to the laboratory for explant set-up, as previously reported [7], [41], [47], [48]. Explant samples were exposed (within 1 hour of collection) to one of two titers of the R5 HIV_BaL_ strain (10^4^ TCID_50_ or 10^2^ TCID_50_); TCID_50_ was determined by titration using pooled PBMCs. The same viral stock used throughout the study. Explants were incubated with virus for 2 hours and then thoroughly washed and six explants from 10 cm and six explants from 30 cm were placed (one biopsy/raft) on gelfoam rafts in individual wells of a 24 well plate (Costar® #3524, Corning, Inc., Corning, NY). Explants were followed for 14 days with supernatants for ELISA quantification of p24 (ng/ml) collected every 3–4 days and replaced with media (100%). Results are reported here as cumulative p24 detected at day 14 with ‘uninfectible’ defined as less than 100 ng p24/ml (p24 kits from AIDS & Cancer Virus Program, NCI, Bethesda, MD; detection to 78 ng/ml). At baseline (Visit 2), all participants' HIV-exposed explant biopsies had a positive control of added UC781 to demonstrate suppressive potential.

The following samples were collected and assessed at Visit 2, 3 & 5:

- rectal microflora to assess changes with swabs collected at baseline and post 7-day exposure (only Visits 2 and 5)
- sloughing of rectal epithelial cells using endoscope-collected lavage at Visits 2, 3 & 5
- histopathology at 10 cm and 30 cm at Visits 2, 3,& 5
- fecal calprotectin to assess mucosal inflammation from post-enema evacuation at Visits 2, 3 & 5
- mucosal mononuclear cell (MMC) phenotype by flow cytometry at Visits 2, 3 & 5
- secreted mucosal immunoglobulins collected by sponge (total IgG, total IgA) at Visits 2, 3-pre and 3-post & 5
- secreted mucosal cytokine proteins collected by sponge at Visits 2, 3-pre and 3-post product exposure & 5
- mucosal tissue mRNA cytokine profile at Visits 2, 3 & 5
- biopsies for *ex vivo* HIV-1 explant infection Visits 2, 3 & 5.

### Plasma levels of UC781

Bridge Laboratories (Gaithersburg, MD) conducted FDA GCP compliant quantifications of plasma levels of UC781 within concentration ranges of 0.25 ng/ml to 200 ng/ml, using a validated LC-MS/MS system (lower limit of quantitation (LLOQ): 0.25 ng/ml). Samples evaluated included all 9 subjects (placebo and two UC781 concentration exposure groups) randomized to undergo the PK plasma sampling (baseline, 30 minutes, 2 hr, 4 hr, 24 hr and post 7-day exposure). Mucosal biopsies were obtained to quantify tissue levels of UC781 once the method had been optimized. The assay was recently validated and results obtained. These are being analyzed and will be reported in a subsequent paper.

### Statistical Analysis

Evaluations of the different data collections were done as follow: Analysis of baseline variability among the three treatment groups was performed for continuous measure using analysis of variance and Kruskal-Wallis test. This exercise assured investigators that baseline ranges here were consistent with those previously published by this group in HPTN-056 [49]. Paired t–test and other appropriate tests were used to compare results from tissue samples collected at the 10 cm and 30 cm biopsy locations (histopathology, tissue cytokine mRNA, flow cytometry and *ex vivo* explant infections). To analyze the treatment effect of the UC781 gel, changes in mucosal indices from baseline to post-single exposure (V3) or post-7-day exposure (V5) were compared between each of the UC781 treatment groups and the HEC placebo group. A two sample t-test was used for continuous variables (tissue mRNA for cytokine, fecal calprotectin, flow cytometry). For continuous variables with two baseline measurements (rectal fluid cytokine profile, immunoglobulins), a mixed linear model with person-level random effect was used. For categorical variables (epithelial sloughing, histopathology), 95% confidence intervals for the difference in correlated proportions were calculated.

### Statistical Analysis of *Ex Vivo* Biopsy Infectibility

#### Suppressive impact of *in vivo* UC781 on *ex vivo* explant infectibility assays

The aim of this explant study was to determine whether *in vivo* administered topical product could suppress *ex vivo* HIV-1 infection in biopsies based on the ancillary study endpoint of cumulative p24 antigen at day 14 (already shown to tightly correlate with qRT-PCR for HIV) [64]. Comparisons evaluated the difference in tissue infectibility between baseline (Visit 2) and post-single dose exposure (Visit 3) or post-7-day exposure (Visit 5) in each study group using paired t-tests. Assessments of the treatment effect on altered infectibility from baseline (Visit 2) to post exposure single (Visit 3) or post-repeated (Visit 5) were conducted at each combination of biopsy locations (10 cm and 30 cm) and viral titers (10^2^, 10^4^) using two sample t-tests. Evaluation of potential baseline differences in biopsy infectibility (10 cm versus 30 cm) for each viral titer used was conducted by using paired t-tests. To determine whether treatment effect differs between the two biopsy locations, a mixed linear model evaluating differences in cumulative day-14 p24 antigen levels was fitted. This model incorporated person-level random effects to account for correlations among biopsies at 10 cm and 30 cm taken from the same individual. Four separate analyses were done, one for each combination of UC781 product (0.1%, 0.25%) and viral titer (10^2^, 10^4^). The mixed model afforded a number of comparisons, among which are a test for treatment effect (product vs. placebo), a test for biopsy location difference (10 cm vs. 30 cm), and a test for location×treatment interaction (addressing whether treatment varies by biopsy location). All biopsy infection results were independently confirmed by a secondary analysis using a comparison of 4 alternative analyses of explant readouts (day 14 p24, AUC, slope, and soft-endpoint analyses [65].

## Results

### Participant Demographics, High Study Retention and Sample Acquisition

The 36 subjects completed the study in 16 months, each requiring a 7–14 week participation including 5 clinic visits, 3 visits for flexible sigmoidoscopies and sample collection, 2 ACASIs and 1 in-depth phone interview. Of 155 potential participants telephonically pre-screened, 55 were eligible for the screening visit (V1) with 36 participants meeting “enrollment” criteria for baseline sampling at Visit 2. All 36 participants initially enrolled completed the study (100% retention). The median age was 41 (range: 24–64) with 26 males (72%) and 10 females (28%). Participants were 41% African American, 39% Caucasian, 14% Hispanic, 3% American Indian and 3% Pacific Islander. Few differences between the participants randomized to each of the 3 study groups were appreciated (see Demographics Table S1).

### No Serious (Grade 3 or 4) Adverse Events (AEs) Reported

Thirty-six AEs were reported of which 28 (78%) were Grade 1 and 8 (22%) were Grade 2. There were no Grade 3 or 4 AEs nor any procedure-related AEs (108 flexible sigmoidoscopies with just over 3000 biopsies were performed in the 36 participants). Only AEs occurring following product exposure (Visit 3 forward) are reported; all are described in Adverse Events Table S2 by number/percentage of subjects having AEs, whether they were gastrointestinal (GI) related or not and which AEs occurred during which trial stage. Table S2 shows that the number of subjects with AEs of any grade are similar between groups after the single or 7-day exposure.

Nearly all the Grade 1 abnormalities (19/28; 68%) were recorded as *not related to product*. Of the 9/28 (32%) Grade 1 AEs reported as *related to product*, 6 were in the placebo group, 2 in the 0.1% UC781 group and 1 the 0.25% UC781 group. Eight Grade 2 AEs were reported among 5 subjects. Four of these 8 reports were from 1 individual (#401 - placebo group) who reported fever, cramps, flatulence, and diarrhea, considered *possibly related*, after the single exposure (Visit 3). Of note, no Grade 2 AEs were seen in this individual following the 7-day exposure. The other 4 Grade 2 AEs included the following: limited diarrhea in 2 subjects following the 7-day exposure and considered *possibly related* (#414 in the 0.25% UC781 group and #418 in the 0.1% UC781 group); transient thrombocytopenia in 1 subject following the 7-day exposure and considered *not related* (#420 in the 0.25% UC781 group); and a spider bite in 1 subject after single dose and considered *not related* (#432 in the placebo group).

### No Epithelial Sloughing from Rectal Lavage

No significant differences at baseline (Visit 2) were identified among enrolled subjects. No differences in the degree of epithelial sloughing between single exposure (Visit 3) and baseline or 7-day exposure (Visit 5) and baseline were appreciated. No UC781 treatment effect was identified for either low-concentration gel or high-concentration gel relative to placebo.

### No Changes in Histology at 10 cm and 30 cm

Neither the placebo nor either of the two UC781 gel concentrations showed significant differences between either single or 7-day exposure and baseline. No differences were seen when each subject's baseline samples at 10 cm and 30 cm were compared using this index of inflammation.

### No Changes in Rectal Microflora

No significant changes in any of the rectal microflora profiles assessed were seen *within* subjects exposed to UC781 (either low or high dose) or the HEC placebo compared to their baseline culture results. As well, no significant differences were seen *between* study groups after cumulative (single and 7-day) exposures (Table 1). Although the study participant subpopulations were too small for statistical evaluation, no trends toward differences were seen between microflora samples from men compared to women (overall) or with *between-group* analyses of men compared to women, including *Lactobacillus* and *Gardnerella vaginalis*.

### No Changes in Fecal Calprotectin

No significant differences in stool calprotectin, an indirect indicator of mucosal inflammation, were seen among study participants at baseline. No significant differences in calprotectin within subjects or between groups were appreciated following single-dose or after 7-day exposure (Table 2).

**Table 2:** Summary of p-values for comparing changes in mucosal immune parameters compared to baseline following single or 7-Day exposure.

		Changes after single exposure (Visit 3-Visit 2) (p-value)		Changes after 7-day exposure (Visit 5 – Visit 2) (p-value)	
		UC781 0.1% vs. Placebo	UC781 0.25% vs. Placebo	UC781 0.1% vs. Placebo	UC781 0.25% vs. Placebo
**Fecal Calprotectin**		0.417	0.587	0.192	0.881
**Mucosal Immunoglobulins** *					
IgG		0.297	0.777	0.747	0.400
IgA		0.754	0.458	0.384	0.503
**Cytokine** *					
RANTES		**0.033** ***	0.569	0.380	0.356
MIP-1 α		0.696	0.406	0.353	0.343
TNF-α		0.436	0.391	0.146	0.229
IFN-γ		0.296	0.282	**0.059** ***	0.211
IL-12 (p40)		0.488	0.428	0.135	0.365
IL-6		0.410	0.761	0.794	0.382
IL-1b**		0.347	0.896	**0.082** ***	0.116
**Tissue mRNA for cytokine**					
IFN-γ	10 cm	0.407	0.546	0.608	0.259
IFN-γ	30 cm	0.999	0.303	0.321	0.959
**Flow Cytometry: Mucosal Mononuclear Cells (MMCs)**					
CD4 lymphocytes	10 cm	0.561	0.806	0.380	0.497
	30 cm	0.258	0.747	0.500	0.536
CD38+/HLA-DR+ on CD4	10 cm	0.467	0.404	0.392	0.946
	30 cm	0.572	0.971	0.346	0.776
CD38 RFI on CD4	10 cm	0.877	0.815	0.340	0.587
	30 cm	0.269	0.299	0.488	0.526
CD38% on CD4	10 cm	0.311	0.397	0.635	0.477
	30 cm	0.698	0.369	0.376	0.646
HLA-DR% on CD4	10 cm	0.307	0.198	0.119	0.737
	30 cm	0.419	0.749	0.984	0.643
CCR5 RFI on CD4	10 cm	0.401	0.103	0.840	**0.025** ***
	30 cm	0.837	0.151	0.226	**0.025** ***
CCR5% on CD4	10 cm	0.702	0.668	0.438	0.643
	30 cm	0.659	0.232	0.926	**0.099** ***
CXCR4% on CD4	10 cm	0.342	0.116	0.587	**0.095** ***
	30 cm	0.511	0.211	0.533	0.112
CCR5%/CXCR4% on CD4	10 cm	0.546	0.372	0.449	0.302
	30 cm	0.516	0.186	0.781	**0.020** ***

*For Mucosal Immunoglobulins and Cytokine, p-values were calculated based on two baseline measurements (Visit 2 and pre-Visit 3).

**One subject's (ID = 417) baseline measurements were extreme outliers and excluded from analysis.

***Statistically significant at alpha level of 0.10.

### Minimal Changes in Mucosal Immunotoxicity Indices

The following data were studied at baseline and compared within and between groups after single-dose and after 7-day exposure: (i) phenotypic, HIV-1 co-receptor and activation profiles of mucosal CD4+ and CD8+ T lymphocytes at 10 and 30 cm; (ii) rectal fluid IgG and IgA immunoglobulins; (iii) rectal fluid cytokine protein yields; and (iv) tissue cytokine mRNA at 10 cm and 30 cm. These unique, baseline data from mucosal samples of healthy HIV-1 seronegative subjects will be enormously helpful to help power future study designs. Consequently, the detailed resultant means and standard deviations for each set of indices are added in Table S3.

Baseline variability was examined for all indices above. For flow cytometric analyses, no significant result was identified among 18 baseline comparisons performed. Importantly, the findings are consistent with those seen in the earlier HPTN-056 study [49], aimed at defining normative mucosal ranges for immunoinflammatory indices. However, using paired t-tests, baseline differences in lymphocyte surface phenotype and activation markers were seen between 10 cm and 30 cm, as we've reported previously [56], [57]. Significant between-site differences at baseline (Visit 2) were identified at a level of α = 0.05 for % CD4+ T cell lymphocytes, CD38 RFI on CD4+ T cell lymphocytes, CD38% on CD4+ T cell lymphocytes, HLA-DR% on CD4+ T cell lymphocytes, CCR5% on CD4+ T cell lymphocytes and % CCR5/CXCR4 double-positives on CD4+ T cell lymphocytes. These may reflect real differences since the total number of comparisons here was 9 [41].

Following intervention, before-after comparisons (baseline to single exposure or baseline to 7-day exposure) were conducted to evaluate changes in each of the above mucosal immune indices in each study group. From the 180 comparisons performed, 26 were significant at α = 0.1. However, at α = 0.05, 11 were significant (closer to the expected number of 9). There were no consistent patterns seen in the distribution of these 26 results. No evidence of consistent mucosal reaction to UC781 treatments or vehicle was identified following either single or 7-day exposure.

Table 2 presents the group analysis results of the clinician-delivered single-dose and self-administered 7-day exposure UC781 treatment effects compared to placebo on changes in all mucosal immune indices. Given the relatively small sample size and given the safety consideration of these first Phase 1 studies, we felt it important not to overlook any possible altered findings associated with exposure. Hence, those comparisons with p-values .10 and lower (corresponding to an alpha of 10%), are highlighted in Table 2, which gives a higher power for identifying significant differences, at the cost of increased Type I error probabilities. For single-dose UC781 treatment effect compared to placebo, on changes in all mucosal immune indices (Visit 3-Visit 2) where 60 comparisons were performed, the single significant result (RANTES in the 0.1% UC781 vs. placebo comparison) is not unusual. The results support the conclusion that neither of the UC781 gel concentrations had any significant effect on mucosal immune indices compared to placebo. Importantly, when the HEC placebo responses were compared to baseline after single dose exposure (Visit 3 vs. Visit 2), no changes were identified. This is the first such detailed assessment of the HEC placebo on rectal mucosa (data not shown).

Following 7-day exposure (Visit 5-Visit 2), an additional 60 comparisons were compared to baseline values (Table 2). Seven significant results were identified at α = 0.1, again, not an unusual number given the number of comparisons. The 4 findings that may have clinical relevance were the following but need to be kept in context with published trends toward differential expression at baseline [41] :

(i-ii) in the higher concentration UC781 group compared to placebo, a difference in the change of CCR5 expression was seen on mucosal CD4+ T lymphocytes at both the 10 cm and 30 cm level (reduced)(P = 0.025 for both)

(iii) for co-expressed CCR5/CXCR4 on CD4+ T lymphocytes at only 30 cm (reduced) (p = 0.020)

(iv) for CXCR4 alone on CD4+ T lymphocytes only at 10 cm (reduced) (p = 0.095).

It should remain clear that the selection of this broad profile of immunoinflammatory indices of mucosal injury represents a best effort to assess any changes of significance related to product (or placebo) in this vulnerable compartment with few standardized measures predicative of clinical toxicity. The overarching absence of changes or differences is relevant.

### Undetectable Plasma Levels of UC781

No detectable levels of UC781 were seen by LC-MS/MS analysis in any samples (LLOQ in plasma: 0.25 ng/ml). As no product was identified in the 7-day exposed participants, the only confirmation of adherence to home use of the product was by participant-completed diary.

### Biopsy Infection studies: Successful Suppression of *ex vivo* HIV-1 Infection of Colorectal Biopsies Exposed *in vivo* to UC781

#### Characteristics of biopsy infections at baseline (Visit 2)

All 36 participants had baseline colonic biopsies obtained from 10 cm and 30 cm immediately set up for *ex vivo* HIV-1 infection with R5 HIV_BaL_ at two titers (10^4^ TCID_50_ and 10^2^ TCID_50_). All but one participants' biopsies were infectible at baseline with the higher 10^4^ TCID_50_ viral titer. Only ∼60% of participants' biopsies were infectible at baseline with the lower 10^2^ TCID_50_ titer. The single individual with biopsies not infectible at baseline with either titer (subject #439) demonstrated normal ranges of surface CCR5 expression on mucosal CD4 T lymphocytes by flow cytometry.

The ∼40% of participants with uninfectible biopsies at baseline with the lower TCID_50_ 10^2^ titer were not the same 40% with uninfectible biopsies at subsequent visits. Over the course of the trial, evaluating only the placebo group (n = 12), as at baseline, all were infectible at all visits with the higher 10^4^ TCID_50_ titer. With the lower 10^2^ viral titer, the 33.3% (subjects #401, 407, 432, 445) not infectible at baseline (Visit 2) was a different 33.3% subset than those not infectible at Visit 3 (subjects #426, 432, 442, 455). At Visit 5, 66.7% of placebo-treated explants were not infectible with the lower viral titer (subjects #407, 416, 417, 426, 432, 442, 445, 455). With the lower viral titer, a participant's biopsies being uninfectible at one visit did not predict uninfectibility at later visits.

To determine whether biopsy infectibility *ex vivo* was impacted by location (10 cm versus 30 cm), baseline p24 infection levels for each titer of virus was compared, using paired t-tests. No evidence for differences in explant infectibility *ex vivo* was seen between two biopsy locations of 10 and 30 cm. These data support using a single site in future similarly designed trials.

#### Single-Dose Exposure: *Ex vivo* infections were suppressible following single-dose in vivo product exposure

**Figure 2:**
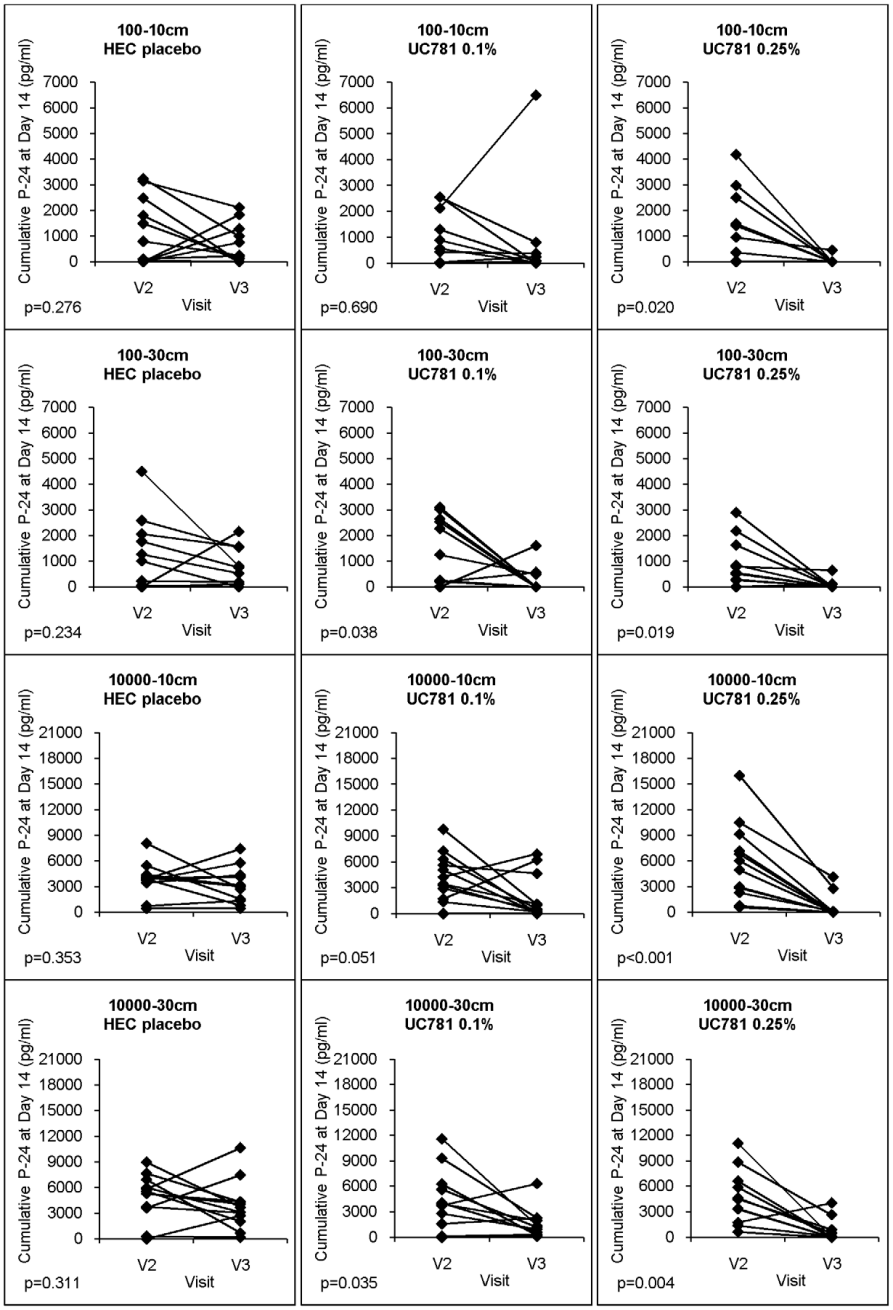
Changes in *ex vivo* infectibility of rectal biopsies following single exposure. The graphs document the impact of the *in vivo* delivered HEC placebo gel, 0.1% UC781 gel or the 0.251% UC781 gel on suppressing *ex vivo* HIV-1 tissue infection. Changes in cumulative p24 antigen at day 14 from biopsies at Visit 2 and Visit 3 are reflective of relative degrees of replicative activity of either low (10^2^ TCID_50_) or high (10^4^ TCID_50_) titer HIV-1_BaL_ in biopsies from either 10cm or 30cm (p values in lower left corner of each figure) after a confirmed 30 minutes of *in vivo* exposure to the defined product.

Data are shown in Figure 2 for the *within-group* suppression of cumulative p24 following *ex vivo* infection of participants' biopsies when exposed *in vivo* to the single dose of the topical product (Visit 3 compared to Visit 2). These products were directly applied by study clinicians. In the 0.25% UC781 treatment group, the sharp decreases in viral infections from Visit 2 to Visit 3 are striking. This decrease is consistent across both virus titer levels and both biopsy locations.

The viral suppression shown in Figure 2 (*right column*) using the 0.25% UC781 formulation are significant at α = 0.05, based on two-sided paired t-tests for the four separate before-after comparisons (10^2^ virus at 10 cm, 10^2^ virus at 30 cm, 10^4^ virus at 10 cm, 10^4^ virus at 30 cm) (*p<0.02*). The same paired before-after comparisons for the 0.1% UC781 formulation demonstrate a trend toward suppression but with marginally significant or non-significant differences (10^2^ virus: p = 0.69 at 10 cm, p = 0.038 at 30 cm; for 10^4^ virus: p = 0.051 at 10 cm, p = 0.035 at 30 cm). None of the placebo comparisons showed a significant change between Visit 2 and Visit 3.

#### Single exposure: Between group differences in biopsy viral suppression

The effect of 0.25% UC781 formulation relative to placebo was significant for 10^4^ TCID_50_ at 10 cm (p = 0.002) and close to significant for 10^4^ TCID_50_ at 30 cm (p = 0.059) at the significance level of α = 0.05.

#### Seven-Day Exposure: Biopsies from 7-day, *in vivo* exposed participants did not demonstrate durable suppression of *ex vivo* infection

**Figure 3:**
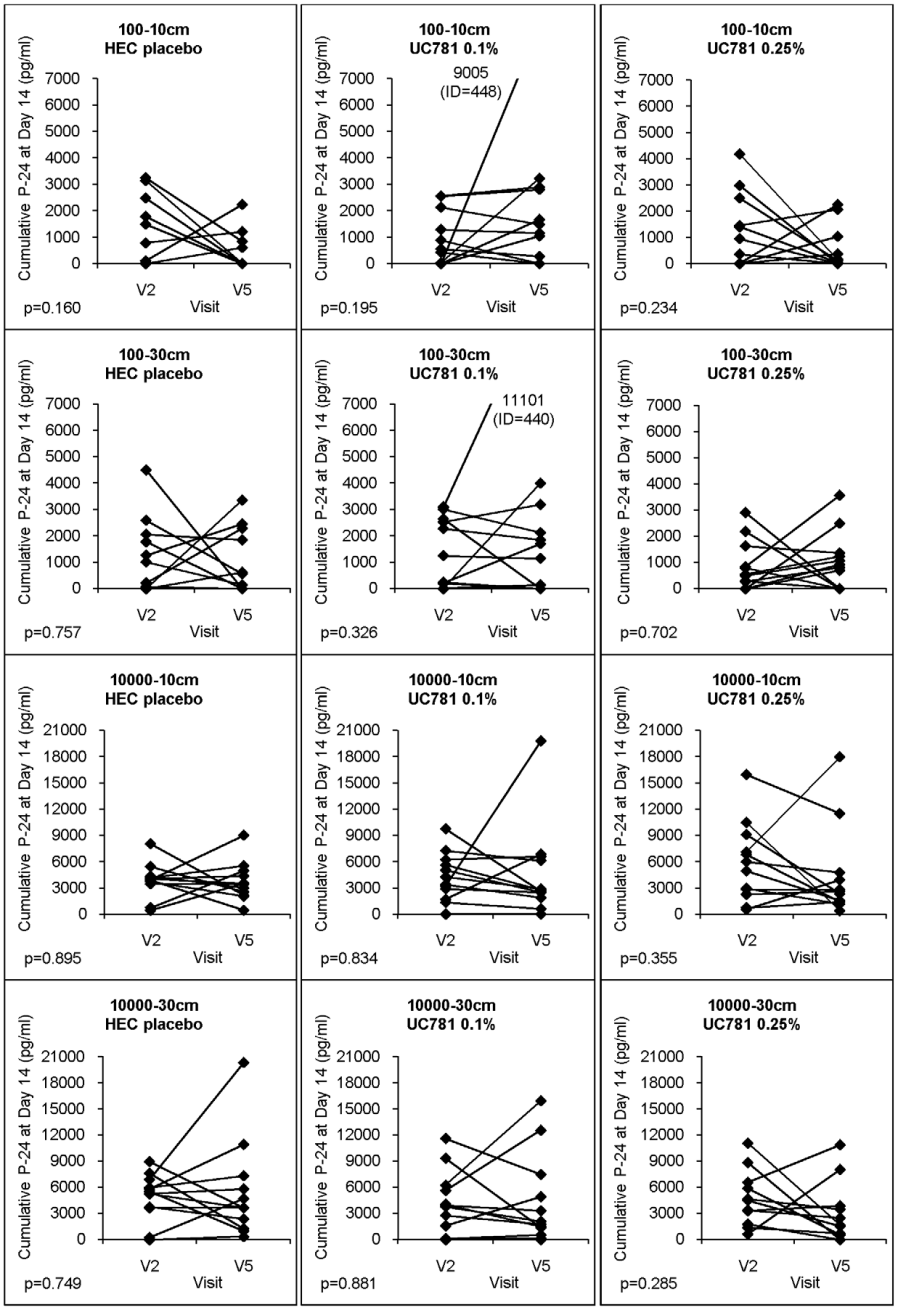
Changes in *ex vivo* infectibility of rectal biopsies following 7-day exposure. The graphs document the impact of the *in vivo* delivered HEC placebo gel, 0.1% UC781 gel or the 0.251% UC781 gel on suppressing *ex vivo* HIV-1 tissue infection. Changes in cumulative p24 antigen at day 14 from biopsies at Visit 2 and Visit 5 are reflective of relative degrees of replicative activity of either low (10^2^ TCID_50_) or high (10^4^ TCID_50_) titer HIV-1_BaL_ in biopsies from either 10 cm or 30 cm (p values in lower left corner of each figure) after a (presumed) week of *in vivo* daily exposure to the defined product.

Figure 3 shows the cumulative p24 effect following *ex vivo* infection of participants' biopsies after the 7-day, at home, self-delivered exposure of the topical product (Visit 5) compared to baseline (Visit 2). None of the paired before-after comparisons in either study group (0.25% UC781, 0.1% UC781, placebo) demonstrated a significant change between Visit 2 and Visit 5 under any combination of virus titers and biopsy locations. *7-day exposure: Between group differences in explant viral suppression*. Between-group reductions were also not seen following the 7-day exposure group comparisons.

#### Mixed model analyses showed “treatment,” not biopsy location, was responsible for the observed significant effects

This approach addressed whether differences in the cumulative p24 between baseline (Visit 2) and post-single exposure (Visit 3) were primarily related to treatment and/or biopsy location. The treatment effect was highly significant for the 0.25% UC781 *in vivo* treated biopsies exposed *ex vivo* to 10^4^ TCID_50_ virus (*p<0.001*). The tests for biopsy location as well as location×treatment interaction were not significant (p = 0.65 and p = 0.40, respectively). The other three analyses (0.1% formulation with 10^2^ TCID_50_, 0.1% formulation with 10^4^ TCID_50_, 0.25% formulation with 10^2^ TCID_50_) also did not show any significant treatment, location or treatment×location effects.

## Discussion

There are five main findings in this first-in-field, Phase 1, randomized, double-blind, placebo-controlled trial of a vaginally formulated HIV-1 microbicide used rectally in 36 men and women: (i) The results showed both doses of the vaginally-formulated UC781 gel used rectally are safe by every index used and are highly acceptable to participants [50]; (ii) The design of the trial was innovative by including two separate exposures (single and 7-day) as independent but linked components to expedite safety assessments; (iii) A novel profile of diverse mucosal “immunotoxicity” indices was included to evaluate potential changes in colorectal mucosa following product exposure, some of which might later point to increased risk of HIV infection; (iv) The immunotoxicity results here correlated well with previously published data from HPTN 056 [49], designed specifically to identify mucosal markers of interest and their stability over 6 weeks; (v) The novel Phase 1 inclusion of *ex vivo* biopsy infectibility studies based on *in vivo* exposed tissue samples was demonstrated as a robust, clinically-relevant, potential early bio-indicator of efficacy. After this trial was completed, it was decided that further development of UC781 would not be pursued because of problems with solubility, stability and other reasons unrelated to this trial. However, findings (ii) through (v) above represent significant advances in the field of microbicide development independent of the product studied.

Colorectal mucosa is composed of a single layer of columnar epithelial cells, extremely receptive to injury but capable of rapid repair. It is highly vulnerable to HIV-1 infection [7], [22], related to the subjacent lamina propria which, in health, is densely populated with activated memory T-cells expressing both CD4 and both HIV-1 co-receptors CCR5 and CXCR4, as well as dendritic cells (DCs) and macrophages [6], [7], [22], [66], [67]. The physiologically inflamed tissue is more infectible per sex act (possibly by 20 to 2000-fold depending on co-STI infections, inflammation, etc) than is the vaginal mucosa [68]–[70]. These differences may also increase rectal compared to vaginal susceptibility to microbicide-induced toxicity, potentially favoring HIV infection [16] as seen with other sexually transmitted infections [31], [33], [34]. The rapidly reactive rectal tissue responses make selecting sample study points difficult, as evidenced by the differing reports and clinical implications seen with N9. Tabet et al. in evaluating an approved vaginal formulation of N9 in MSM [16], described mild to no rectal histological changes in participants receiving up to 6 weeks of daily N9 (or placebo gel) with samples collected up to 12 hours after N9 exposure [15]. In contrast, Phillips et al. using rectal lavage and histology endpoints, saw marked rectal epithelial exfoliation 15 minutes after a single N9 topical exposure in men which disappeared after 2 hours [14].

In this Phase 1 trial, the rectally-applied, vaginally-formulated UC781 0.1% and 0.25% gel and the HEC placebo were safe. There were no Grade 3 or higher AEs reported nor any procedure-related AEs despite over 3000 biopsies having been performed. Retention was 100% post-enrollment, emphasizing participant's understanding and willingness to undergo these procedures repeatedly.

Acceptability (using web-based surveys and an in-depth interview at study's end) was high, especially when focusing on “likelihood” to use the product in the future [50]. The importance of determining acceptability in the early phases of microbicide development is critical, enabling earlier, less costly product changes and raising the likelihood of real-life use.

The “immunotoxicity assays” as a whole, showed no significant changes from baseline for each product or any significant differences between study groups. This was true for both the 10 cm and 30 cm assessments following the single-dose and the 7-day dose exposures. It is reassuring that the baseline results here were generally within the normative ranges reported in HPTN 056 [49]. This confirmation is important to the field.

One of the most impactful endpoints in this Phase 1 trial was the first-time attempt to infect *ex vivo* tissue biopsies that had been exposed to drug only *in vivo*. Given limits on the number of biopsies obtainable, only the laboratory strain of R5 HIV_BaL_ was used at two titers (TCID_50_ of 10^4^ and 10^2^ using the same lot of virus). These titers are thought to be far in excess of ejaculate concentrations [43]–[45]. Colorectal explant techniques have been increasingly used for HIV-1 infectibility readouts from a variety of tissue types [64], [71], [72] and evaluated in a multi-center standardization process conducted within the NIH sponsored Microbicide Quality Assurance Program [65]. Currently, there are two freshly-acquired, colorectal explant models: the “sealed edge” model optimizing polarization and orientation for virus exposure [73] and the “exposed edge” model [41] which simply places the explant on a gelfoam raft, with virus access to all surfaces. This trial utilized the second model as we felt it more accurately reflected clinically relevant situations where preparatory enemas and rectal intercourse are often traumatic to the epithelia, potentially providing HIV with direct access to sub-epithelial target cells.

The higher viral titer reproducibly infected nearly all subjects' colorectal biopsies at baseline while the lower viral titer infected only 60%. Notably, the 40% not infectible with this lower titer at baseline were not always the same 40% uninfectible at later time points (placebo arm). This supports an interpretation that infection at this titer (closer to clinically relevant titers) could be a statistically random event or reflect *intra*-subject biological variables, such as innate defenses, at the mucosal surface. For use in smaller, exploratory and Phase 1 trials, if baseline infectibility is needed to optimize power calculations and smaller enrollment numbers, it appears a higher viral titer would be preferred to insure baseline infectibility.

### Evaluating the data from the directly-applied, single topical gel exposure

Statistically significant suppression of *ex vivo* biopsy infections was seen in the high-concentration gel group (0.25%) after a single exposure compared to baseline. This was seen with *both* the high and low titered virus. Between-group comparisons (UC781 gel compared to placebo) demonstrated that the treatment effect was significant, even with these small subject numbers. This was seen only with the higher viral titer. The data are compelling when evaluating the 30 minute topical exposure of the higher UC781 concentration (0.25% gel) at 10 cm (area most likely drug exposed) using the *higher* viral titer (assuring baseline infectibility). UC781 is a potent inhibitor of viral RT. For this to be the main mediator of suppression in these heavily washed biopsies, other mechanisms may need to be invoked and tested, including an intracellular activity and/or retained drug in tissue/membranes due to the lipophilic nature of UC781 inhibiting further replication. This latter observation is the known (but poorly understood) “memory” effect of UC781. A clinically relevant advantage may be prolonged resistance to transmission due to this feature of UC781, possibly allowing for coitally-dissociated administration.

These results showing suppression of *ex vivo* biopsy infection were consistent and reproducible, within groups and between groups. Given that these are clinical samples with expected inter-subject variability and washed frequently by shaking in media and during transport from the endoscopy unit to the laboratory, it is remarkable that any *ex vivo* impact was seen with only 12 participants in each study group. Additional confounders (that might have taken years to segregate out) include *in vivo* variables such as the biological activity of the colorectal area (continuous mucus secretion, peristaltic activity, frequent stool passage) and the absence of an independent, visual identifier to guide biopsy acquisition relative to where the investigator-administered topical drug might be located 30 minutes after exposure.

### Evaluating the 7-day (uncontrolled) exposure

As no independent, reliable biological indicator of home delivery/compliance was used (a great need in this field), there are many contributory explanations for the apparent absence of *ex vivo* suppression of biopsy infections. Maintained suppression would be dependent on the subjects using the product daily, as instructed, and that there be some tissue penetration/retention, given the active GI elimination processes. In addition, some of the biochemical, lipophilic features of UC781 as well as its tissue half-life *in vivo* remain undefined. When coupled with complex mucosal factors of mucus, microflora and dynamic epithelial turnover, the absence of 7-day exposed *ex vivo* suppression is more understandable and points out challenges to address in future trials.

At this point, the *ex vivo* biopsy infection suppression assays may be viewed as a possible bioindicator of pre-clinical efficacy. Importantly for future trials: (i) no differences were seen using the 30 cm or the 10 cm biopsies, supporting the selection of a single anatomical site in similarly designed trials (benefiting participant safety as well) and (ii) using a sufficiently high titer of standardized virus minimizes data loss due to uninfectible baseline samples.

### Conclusions

The microbicide field has met with significant difficulties in attempting to move a product from design through development to demonstrating efficacy. The first hopeful signs were the dramatic results from CAPRISA 004 [10]. While not discussed much 10 years ago, awareness of the high prevalence of receptive anal intercourse (RAI) in heterosexual partnering [24]–[30], [74]–[80] as well as MSM, combined with the enhanced infectibility per sex act of this compartment has driven the microbicide field to actively include development, assessment and acceptability of rectal microbicides into the main portfolio of scientific and public health goals.

Many advances in technology, immunotoxicity assessments, PK and pharmacodynamic (PD) study design, formulations and acceptability measures have enabled the development of more strategic and focused efforts with rectal microbicides [41], [46], [47], [49], [54], [63], [64], [81]–[85], supporting a rapid advance to clinical trials. While development of a combination microbicide useable in both sexual compartments is a long-term desired goal, the current need for a safe and effective rectal microbicide is evident. The inclusion of a novel *ex vivo* biomarker of efficacy in a Phase 1 trial is a boon to the field and may turn out to be an exciting addition as a “nonclinical surrogate of bio-efficacy,” helping guide decisions about which agents at which concentrations should be advanced in human trials. These intensive efforts and mucosal indices benefit the microbicide field as well as the growing focus on mucosal vaccines, enabling a more rational and cost-effective product development pipeline.

## Supporting Information

Table S1 Demographics.
(PDF)

Table S2 Adverse Events.
(PDF)

Table S3 Means and Standard Deviations.
(PDF)

Protocol S1 Trial Protocol.
(PDF)

Checklist S1 CONSORT Checklist.
(DOC)
