# Supplementary material for: First Phase 1 Double-Blind, Placebo-Controlled, Randomized Rectal Microbicide Trial Using UC781 Gel with a Novel Index of Ex Vivo Efficacy
Source: PLoS One. 2011 Sep 28;6(9):e23243. doi: 10.1371/journal.pone.0023243 (PMC3182160; doi:10.1371/journal.pone.0023243)
Supplement: Table S1 — Demographics. (PDF) [file pone.0023243.s001.pdf]

Demographics Table S1

Characteristics	TOTAL(n=36)		PLACEBO (n=12)		UC781 0.1% (n=12)		UC781 0.25% (n=12)	
	Male	Female	Male	Female	Male	Female	Male	Female
	n=26 (72%)	n=10 (28%)	n=10 (83%)	n=2 (17%)	n=7 (58%)	n=5 (42%)	n=9 (75%)	n=3 (25%)
**Age, years (median ± range)**	**42** (24-63)	**41.5** (25-64)	**43** (24-52)	**46** (25-64)	**49** (29-63)	**41** (25-51)	**35** (26-53)	**44** (29-44)
**Weight, lbs (median ± range)**	**183.7** (116-312)	**166.1*** (117-379)	**190.2** (125-312)	**212** (166-258)	**167.2** (116-203)	**192.9*** (148-238)	**187** (132-260)	**136.5** (126-250)
**Race**								
African-American	**11** (42%)	**4** (40%)	**3** (30%)	**0**	**2** (28%)	**4** (80%)	**6** (66.6%)	**0**
Asian or Pacific Islander	**1** (4%)	**0**	**0**	**0**	**1** (14%)	**0**	**0**	**0**
Native American	**1** (4%)	**1** (10%)	**0**	**0**	**0**	**0**	**1** (11%)	**1** (33.3%)
White or European American	**13** (50%)	**5** (50%)	**7** (70%)	**2** (100%)	**4** (57%)	**1** (20%)	**2** (22%)	**2** (66.6%)
**Ethnicity**								
Hispanic or Latino	**4** (15%)	**1** (10%)	**1** (10%)	**0**	**2** (29%)	**0**	**1** (11%)	**1** (33.3%)
Not Hispanic or Latino	**21** (81%)	**8** (80%)	**8** (80%)	**2** (100%)	**5** (71%)	**4** (80%)	**8** (89%)	**2** (66.6%)
Prefer not to answer	**1** (4%)	**1** (10%)	**1** (10%)	**0**	**0**	**1** (20%)	**0**	**0**
**Education, years**								
Less than 12	**4** (15%)	**3** (30%)	**1** (10%)	**0**	**1** (14%)	**2** (40%)	**2** (22%)	**1**(33.3%)
12 - 16	**18** (69%)	**5** (50%)	**7** (70%)	**1** (50%)	**4** (57%)	**2** (40%)	**7** (78%)	**2** (66.6%)
More than 16	**4** (15%)	**1** (10%)	**2** (20%)	**1** (50%)	**2** (28%)	**0**	**0**	**0**
Prefer not to answer	**0**	**1** (10%)	**2** (20%)	**0**	**0**	**1** (20%)	**0**	**0**
**Income (from previous year)**								
$10,000 or less	**8** (31%)	**4** (40%)	**3** (30%)	**0**	**2** (28%)	**3** (60%)	**3** (33.3%)	**1** (33.3%)
$10,001 to $20,000	**8** (31%)	**2** (20%)	**2** (20%)	**0**	**4** (57%)	**1** (20%)	**2** (22%)	**1** (33.3%)
$20,001 to $40,000	**3** (11%)	**1** (10%)	**1** (10%)	**1** (50%)	**0**	**0**	**2** (22%)	**0**
$40,001 to $60,000	**3** (11%)	**0**	**2** (20%)	**0**	**1** (14%)	**0**	**0**	**0**
Over $80,000	**1** (4%)	**0**	**1** (10%)	**0**	**0**	**0**	**0**	**0**
Don't Know	**1** (4%)	**1** (10%)	**0**	**1** (50%)	**0**	**0**	**1** (11%)	**0**
Prefer not to Answer	**2** (8%)	**2** (20%)	**1** (10%)	**0**	**0**	**1** (20%)	**1** (11%)	**1** (33.3%)

* one female participant was not weighed at baseline
