# Supplementary material for: First Phase 1 Double-Blind, Placebo-Controlled, Randomized Rectal Microbicide Trial Using UC781 Gel with a Novel Index of Ex Vivo Efficacy
Source: PLoS One. 2011 Sep 28;6(9):e23243. doi: 10.1371/journal.pone.0023243 (PMC3182160; doi:10.1371/journal.pone.0023243)
Supplement: Table S2 — Adverse Events. (PDF) [file pone.0023243.s002.pdf]

Table S2: Gastrointestinal-Related Adverse Events (AE) in Enrolled Participants per Subject Number, Group and Trial Stage

Adverse Events (AEs) (n=36)	Subjects (total)	AEs (total)	Single Dose Exposure						7-Day Exposure					
			Placebo		UC781: 0.1%		UC781: 0.25%		Placebo		UC781: 0.1%		UC781: 0.25%	
	(n=36) (%)	N / Total AEs (n=# of subjects)	# of subjects (n=12)(%)	# of AEs (# of subjects)	# of subjects (n=12)(%)	# of AEs (# of subjects)	# of subjects (n=12)(%)	# of AEs (# of subjects)	# of subjects (n=12)(%)	# of AEs (# of subjects)	# of subjects (n=12)(%)	# of AEs (# of subjects)	# of subjects (n=12)(%)	# of AEs (# of subjects)
Subjects with No AEs Reported	**7/36 (19%)**	***NA***												
**AE: Grade 1**	**15/36** (42%)	**28** (15 subjects)	**2** (17%)	**9** (2 subjects)	**4**(33%)	**5** (4 subjects)	**4** (33%)	**5** (4 subjects)	**2** (17%)	**4** (2 subjects)	**1** (8%)	**1** (1 subject)	**4** (33%)	**4** (4 subjects)
⇨Related to Product	**1/36** (3%)	**2** (1 subject)	**1** (8%)	**2**(1 subject)	**0**	**0**	**0**	**0**	**0**	**0**	**0**	**0**	**0**	**0**
**→GI**	**1/36** (3%)	**2** (1 subject)	**1**(8%)	**2** (1 subject)	**0**	**0**	**0**	**0**	**0**	**0**	**0**	**0**	**0**	**0**
Hematochezia	1/36 (3%)	1 (1 subect)	1 (8%)	1 (1 subect)	0	0	0	0	0	0	0	0	0	0
Tenderness	1/36 (3%)	1 (1 subect)	1 (8%)	1 (1 subect)	0	0	0	0	0	0	0	0	0	0
**→non-GI**	0	0	0	0	0	0	0	0	0	0	0	0	0	0
⇨Possibly Related to Product	**3/36** (8%)	**6** (3 subjects)	**1** (8%)	**3** (1 subject)	**1** (8%)	**1** (1 subject)	**0**	**0**	**1** (8%)	**1** (1 subject)	**0**	**0**	**1**(8%)	**1** (1 subject)
**→GI**	**2/36** (6%)	**2** (2 subjects)	**1** (8%)	**1** (1 subect)	**1** (8%)	1 (1 subject)	**0**	**0**	**0**	**0**	**0**	**0**	**0**	**0**
Pain w/defecation	1/36 (3%)	1 (1 subect)	1 (8%)	1 (1 subect)	0	0	0	0	0	0	0	0	0	0
Throbbing - rectum	1/36 (3%)	1 (1 subect)	0	0	1 (8%)	1 (1 subject)	0	0	0	0	0	0	0	0
**→non-GI**	**1/36** (3%)	**4** (2 subjects)	**1** (8%)	**2** (1 subject)	**0**	**0**	**0**	**0**	**1** (8%)	**1** (1 subject)	**0**	**0**	**1** (8%)	**1** (1 subject)
⇨Probably Related to Product	**1/36** (3%)	**1** (1 subect)	**0**	**0**	**1** (8%)	**1** (1 subject)	**0**	**0**	**0**	**0**	**0**	**0**	**0**	**0**
**→GI**	**1/36 (**3%)	**1** (1 subect)	**0**	**0**	**1** (8%)	**1** (1 subject)	**0**	**0**	**0**	**0**	**0**	**0**	**0**	**0**
Bloating	1/36 (3%)	1 (1 subect)	0	0	1(8%)	1 (1 subject)	0	0	0	0	0	0	0	0
**→non-GI**	**0**	**0**	**0**	**0**	**0**	**0**	**0**	**0**	**0**	**0**	**0**	**0**	**0**	**0**
⇨Not related to Product	**13/36** (36%)	**19** (13 subjects)	**2** (17%)	**4** (2 subjects)	**3** (25%)	**3** (3 subjects)	**4** (33%)	**5** (4 subjects)	**2** (17%)	**3** (2 subjects)	**1** (8%)	**1** (1 subject)	**3** (25%)	**3** (3 subjects)
**→GI**	**3/36** (8%)	**5** (3 subjects)	**1** (8%)	**2** (1 subject)	**0**	**0**	**1** (8%)	**2** (1 subject)	**0**	**0**	**1** (8%)	**1** (1 subject)	**0**	**0**
Acid reflux	1/36 (3%)	1 (1 subject)	1 (8%)	1 (1 subject)	0	0	0	0	0	0	0	0	0	0
Cramping	1/36 (3%)	1 (1 subject)	0	0	0	0	1(8%)	1 (1 subject)	0	0	0	0	0	0
Diarrhea	1/36 (3%)	1 (1 subject)	0	0	0	0	1(8%)	1 (1 subject)	0	0	0	0	0	0
Eructation	1/36 (3%)	1 (1 subject)	1 (8%)	1 (1 subject)	0	0	0	0	0	0	0	0	0	0
Polyps - Colon	1/36 (3%)	1 (1 subject)	0	0	0	0	0	0	0	0	1(8%)	1 (1 subject)	0	0
**→non-GI**	**12/36** (33%)	**14** (12 subjects)	**2** (17%)	**2** (1 subject)	**3** (25%)	**3** (3 subjects)	**3** (25%)	**3** (3 subjects)	**2** (17%)	**3** (2 subjects)	**0**	**0**	**3** (25%)	**3** (3 subjects)
**AE: Grade 2**	**5/36** (14%)	**8** (5 subjects)	**1** (8%)	**4** (1 subject)	**0**	**0**	**1** (8%)	**1** (1 subject)	**1** (8%)	**1** (1 subject)	**1** (8%)	**1** (1 subject)	**1** (8%)	**1** (1 subject)
⇨Possibly Related to Product	**3/36** (8%)	**6** (3 subjects)	**1** (8%)	**4** (1 subject)	**0**	**0**	**1** (8%)	**1** (1 subject)	**0**	**0**	**1** (8%)	**1** (1 subject)	**0**	**0**
**→GI**	**3/36** (8%)	**5** (3 subjects)	**1** (8%)	**3** (1 subject)	**0**	**0**	**1** (8%)	**1** (1 subject)	**0**	**0**	**1** (8%)	**1** (1 subject)	**0**	**0**
Cramping	1/36 (3%)	1 (1 subect)	1(8%)	1 (1 subject)	0	0	0	0	0	0	0	0	0	0
Diarrhea	3/36 (8%)	3 (3 subjects)	1 (8%)	1 (1 subject)	0	0	1(8%)	1 (1 subject)	0	0	1 (8%)	1 (1 subject)	0	0`
**Flatulence**	1/36 (3%)	1 (1 subject)	1 (8%)	1 (1 subject)	0	0	0	0	0	0	0	0	0	0
**→non-GI**	**1/36** (3%)	**1** (1 subject)	**1** (8%)	**1** (1 subject)	**0**	**0**	**0**	**0**	**0**	**0**	**0**	**0**	**0**	**0**
⇨Not related to Product	**2/36** (6%)	**2** (2 subjects)	**0**	**0**	**0**	**0**	**0**	**0**	**1** (8%)	**1** (1 subject)	**0**	**0**	**1** (8%)	**1** (1 subject)
**→GI**	**0**	**0**	**0**	**0**	**0**	**0**	**0**	**0**	**0**	**0**	**0**	**0**	**0**	**0**
**→non-GI**	**2/36** (6%)	**2** (2 subjects)	**0**	**0**	**0**	**0**	**0**	**0**	**1** (8%)	**1** (1 subject)	**0**	**0**	**1** (8%)	**1** (1 subject)
**AE: Grade 3**	**0**	**0**	**0**	**0**	**0**	**0**	**0**	**0**	**0**	**0**	**0**	**0**	**0**	**0**
None reported	0	0	0	0	0	0	0	0	0	0	0	0	0	0
**AE: Grade 4**	**0**	**0**	**0**	**0**	**0**	**0**	**0**	**0**	**0**	**0**	**0**	**0**	**0**	**0**
None reported	0	0	0	0	0	0	0	0	0	0	0	0	0	0

* gastointestinal
