# Supplementary material for: First Phase 1 Double-Blind, Placebo-Controlled, Randomized Rectal Microbicide Trial Using UC781 Gel with a Novel Index of Ex Vivo Efficacy
Source: PLoS One. 2011 Sep 28;6(9):e23243. doi: 10.1371/journal.pone.0023243 (PMC3182160; doi:10.1371/journal.pone.0023243)
Supplement: Table S3 — Means and Standard Deviations. (PDF) [file pone.0023243.s003.pdf]

Table 3S. Means and Standard Deviations of All Mucosal Immune Parameters Studied.

Each section below shows absolute numbers for Placebo (n=12), low dose 0.1% UC781 (n=12), and high-dose 0.25% UC781 (n=12). The left column ("All; Baseline (Visit 2)") reports the baseline mean ± standard deviation of all 36 participants. The Baseline (Visit 2) under the treatment headings reports the mean ± standard deviation for only the 12 participants randomized to that treatment group.

		All	Placebo		
		Baseline (Visit 2)	Baseline (Visit 2)	Single dose (Visit 3)	7-day (Visit 5)
**Fecal Calprotectin (μg)**		28.31 ± 37.98	14.64 ± 10.58	47. 55 ± 50.29	19.68 ± 18.77
**Mucosal Immunoglobulins (ng/ml)***					
IgG		10495.31 ± 11244.64	10941.53 ± 14078.75	20897.92 ± 18331.14	8639.33 ± 7383.88
IgA		52630.70 ± 50376.76	66453.47 ± 65092.63	34430.06 ± 27544.98	64633.53 ± 70417.68
**Cytokine (pg/ml)***					
RANTES		78.51 ± 90.55	107.84 ± 128.85	71.79 ± 54.38	50.49 ± 42.02
MIP-1 α		63.07 ± 125.77	88.24 ± 182.64	64.65 ± 187.98	39.69 ± 132.29
TNF-α		11.85 ± 14.65	18.35 ± 21.07	25.95 ± 48.29	8.29 ± 9.21
IFN-γ		36.24 ± 53.73	53.64 ± 79.47	38.58 ± 75.39	25.68 ± 32.15
IL-12 (p40)		16.70 ± 44.06	31.44 ± 75.59	121.51 ± 391.05	7.50 ± 4.46
IL-6		15.05 ± 31.14	20.90 ± 50.79	38.69 ± 79.64	13.52 ± 17.81
IL-1b**		12.85 ± 8.22	12.44 ± 8.79	11.84 ± 10.72	11.64 ± 9.79
**Tissue mRNA for cytokine (copies/10^6actin)**					
IFN-γ	10cm	8715.30 ± 7855.91	7770.23 ± 7541.87	6618.19 ± 5282.25	4877.41 ± 3085.39
IFN-γ	30cm	5962.46 ± 6433.41	5184.60 ± 4005.53	4245.10 ± 2991.43	6258.32 ± 6227.90
**Flow Cytometry: Mucosal Mononuclear Cells (MMCs)**					
CD4 lymphocytes	10cm	43.07 ± 9.16	44.47 ± 9.44	42.98 ± 6.82	40.58 ± 10.85
	30cm	47.23 ± 9.37	49.28 ± 9.57	48.59 ± 7.94	47.66 ± 8.98
CD38+/HLA-DR+ on CD4	10cm	2.35 ± 2.35	1.38 ± 1.25	1.84 ± 0.73	1.83 ± 1.04
	30cm	2.44 ± 2.54	1.61 ± 1.24	1.91 ± 0.94	2.05 ± 1.26
CD38 RFI on CD4	10cm	109.13 ± 16.46	104.22 ± 6.50	105.06 ± 6.55	109.49 ± 8.81
	30cm	114.33 ± 21.23	109.22 ± 13.74	101.34 ± 29.45	110.61 ± 8.36
CD38% on CD4	10cm	11.86 ± 8.83	8.30 ± 5.76	11.06 ± 4.50	11.68 ± 6.12
	30cm	9.98 ± 6.13	7.42 ± 4.06	8.72 ± 3.63	9.29 ± 5.22
HLA-DR% on CD4	10cm	16.95 ± 7.88	13.34 ± 8.29	13.58 ± 4.97	13.87 ± 6.34
	30cm	20.41 ± 6.79	18.67 ± 6.23	18.16 ± 7.26	18.01 ± 5.87
CCR5 RFI on CD4	10cm	28.58 ± 8.84	27.57 ± 8.67	31.85 ± 6.67	33.37 ± 11.69
	30cm	28.93 ± 8.37	28.74 ± 8.17	30.43 ± 6.75	31.15 ± 9.07
CCR5% on CD4	10cm	69.38 ± 13.08	71.57 ± 15.28	72.68 ± 15.48	70.42 ± 10.56
	30cm	75.45 ± 9.59	75.89 ± 11.20	78.60 ± 9.86	75.60 ± 11.08
CXCR4% on CD4	10cm	82.69 ± 11.82	82.11 ± 17.33	86.39 ± 6.83	84.35 ± 11.82
	30cm	82.52 ± 12.17	82.89 ± 17.31	85.89 ± 5.93	84.45 ± 11.61
CCR5%/CXCR4% on CD4	10cm	58.07 ± 15.02	60.13 ± 19.88	62.33 ± 11.87	60.45 ± 13.84
	30cm	62.91 ± 12.62	63.85 ± 16.83	67.44 ± 8.08	65.31 ± 14.27
			**UC781 0.1% (Low Dose)**		
**Fecal Calprotectin (μg)**		28.31 ± 37.98	19.59 ± 20.11	26.50 ± 22.14	42.68 ± 44.70
**Mucosal Immunoglobulins (ng/ml)***					
IgG		10495.31 ± 11244.64	12853.83 ± 12904.91	16406.25 ± 14380.43	10262.44 ± 7879.26
IgA		52630.70 ± 50376.76	48420.69 ± 36221.82	29201.14 ± 23296.60	68105.58 ± 80150.75
**Cytokine (pg/ml)***					
RANTES		78.51 ± 90.55	79.09 ± 71.45	31.06 ± 17.21	109.57 ± 162.56
MIP-1 α		63.07 ± 125.77	52.20 ± 75.56	37.44 ± 53.80	36.84 ± 51.11

TNF-α		11.85 ± 14.65	6.96 ± 7.37	11.52 ± 13.01	9.78 ± 9.52
IFN-γ		36.24 ± 53.73	22.20 ± 22.06	19.86 ± 13.89	36.79 ± 41.73
IL-12 (p40)		16.70 ± 44.06	11.15 ± 9.25	8.38 ± 3.66	9.95 ± 4.25
IL-6		15.05 ± 31.14	12.79 ± 15.80	40.77 ± 95.73	13.89 ± 18.27
IL-1b**		12.85 ± 8.22	12.23 ± 7.23	11.60 ± 6.13	15.80 ± 11.99
**Tissue mRNA for cytokine (copies/10^{6}actin)**					
IFN-γ	10cm	8715.30 ± 7855.91	12988.17 ± 10018.13	9417.80 ± 7300.04	11952.62 ± 11025.03
IFN-γ	30cm	5962.46 ± 6433.41	9253.69 ± 9308.77	8309.88 ± 8668.13	7208.68 ± 6114.18
**Flow Cytometry: Mucosal Mononuclear Cells (MMCs)**					
CD4 lymphocytes	10cm	43.07 ± 9.16	40.40 ± 7.42	40.60 ± 6.75	39.06 ± 7.02
	30cm	47.23 ± 9.37	43.35 ± 8.06	45.49 ± 9.04	44.02 ± 10.13
CD38+/HLA-DR+ on CD4	10cm	2.35 ± 2.35	3.45 ± 3.40	3.01 ± 1.91	2.41 ± 1.05
	30cm	2.44 ± 2.54	3.51 ± 3.93	4.14 ± 3.36	3.34 ± 1.94
CD38 RFI on CD4	10cm	109.13 ± 16.46	116.25 ± 22.36	115.92 ± 15.85	133.67 ± 42.40
	30cm	114.33 ± 21.23	124.61 ± 30.69	129.23 ± 24.50	128.61 ± 26.03
CD38% on CD4	10cm	11.86 ± 8.83	13.89 ± 9.13	13.19 ± 5.44	14.54 ± 4.66
	30cm	9.98 ± 6.13	11.77 ± 7.74	13.10 ± 6.95	13.58 ± 8.22
HLA-DR% on CD4	10cm	16.95 ± 7.88	20.25 ± 8.83	16.48 ± 6.53	14.77 ± 5.35
	30cm	20.41 ± 6.79	22.66 ± 8.31	24.18 ± 8.43	20.99 ± 6.36
CCR5 RFI on CD4	10cm	28.58 ± 8.84	28.63 ± 10.47	29.88 ± 10.05	36.56 ± 9.57
	30cm	28.93 ± 8.37	28.06 ± 9.70	29.16 ± 8.07	38.07 ± 20.22
CCR5% on CD4	10cm	69.38 ± 13.08	68.01 ± 13.71	66.82 ± 10.58	68.87 ± 8.21
	30cm	75.45 ± 9.59	75.40 ± 10.55	75.21 ± 9.86	75.56 ± 8.22
CXCR4% on CD4	10cm	82.69 ± 11.82	83.11 ± 9.10	82.12 ± 6.07	86.79 ± 3.74
	30cm	82.52 ± 12.17	82.85 ± 9.78	81.70 ± 5.99	86.97 ± 5.53
CCR5%/CXCR4% on CD4	10cm	58.07 ± 15.02	57.15 ± 14.34	54.99 ± 10.37	59.75 ± 8.93
	30cm	62.91 ± 12.62	63.07 ± 11.70	61.49 ± 10.10	65.98 ± 8.96
		**UC781 0.25% (High Dose)**			
**Fecal Calprotectin (μg)**		28.31 ± 37.98	55.67 ± 59.68	78.45 ± 93.67	58.55 ± 85.65
**Mucosal Immunoglobulins (ng/ml)***					
IgG		10495.31 ± 11244.64	7690.56 ± 4764.43	25137.50 ± 30041.19	14222.22 ± 10088.32
IgA		52630.70 ± 50376.76	43017.94 ± 46874.56	43142.69 ± 23748.71	38075.17 ± 32835.18
**Cytokine (pg/ml)***					
RANTES		78.51 ± 90.55	48.59 ± 49.66	109.37 ± 105.42	98.84 ± 95.65
MIP-1 α		63.07 ± 125.77	48.78 ± 101.09	19.85 ± 53.57	46.32 ± 85.42
TNF-α		11.85 ± 14.65	10.23 ± 10.48	11.31 ± 9.83	18.23 ± 28.81
IFN-γ		36.24 ± 53.73	32.58 ± 41.48	34.19 ± 52.24	62.72 ± 91.94
IL-12 (p40)		16.70 ± 44.06	7.50 ± 3.65	8.27 ± 4.53	8.12 ± 5.22
IL-6		15.05 ± 31.14	11.45 ± 14.12	17.16 ± 11.96	66.41 ± 188.53
IL-1b**		12.85 ± 8.22	13.85 ± 9.21	12.00 ± 7.00	20.53 ± 21.96
**Tissue mRNA for cytokine (copies/10^{6}actin)**					
IFN-γ	10cm	8715.30 ± 7855.91	5387.51 ± 2480.25	5288.72 ± 3741.45	4792.16 ± 3113.99
IFN-γ	30cm	5962.46 ± 6433.41	3449.09 ± 3102.53	4266.63 ± 2993.29	4416.31 ± 3248.06
**Flow Cytometry: Mucosal Mononuclear Cells (MMCs)**					
CD4 lymphocytes	10cm	43.07 ± 9.16	44.34 ± 10.53	43.56 ± 9.66	42.57 ± 5.80
	30cm	47.23 ± 9.37	49.05 ± 9.91	47.72 ± 9.03	45.37 ± 9.05
CD38+/HLA-DR+ on CD4	10cm	2.35 ± 2.35	2.31 ± 1.55	2.27 ± 1.07	2.69 ± 1.04
	30cm	2.44 ± 2.54	2.27 ± 1.41	2.49 ± 1.26	2.73 ± 1.35

CD38 RFI on CD4	10cm	109.13 ± 16.46	107.36 ± 16.11	108.27 ± 10.60	116.51 ± 13.65
	30cm	114.33 ± 21.23	109.62 ± 12.85	111.64 ± 9.83	115.83 ± 18.45
CD38% on CD4	10cm	11.86 ± 8.83	13.70 ± 10.68	14.33 ± 6.54	15.38 ± 4.96
	30cm	9.98 ± 6.13	11.00 ± 5.79	13.97 ± 7.00	13.60 ± 4.07
HLA-DR% on CD4	10cm	16.95 ± 7.88	17.59 ± 4.84	14.25 ± 3.18	16.50 ± 5.20
	30cm	20.41 ± 6.79	20.08 ± 5.58	18.14 ± 4.67	19.89 ± 6.46
CCR5 RFI on CD4	10cm	28.58 ± 8.84	29.63 ± 7.74	29.40 ± 6.87	29.97 ± 7.86
	30cm	28.93 ± 8.37	30.10 ± 7.68	27.65 ± 6.74	29.01 ± 7.15
CCR5% on CD4	10cm	69.38 ± 13.08	68.49 ± 10.46	66.60 ± 7.69	65.44 ± 9.88
	30cm	75.45 ± 9.59	75.04 ± 7.18	70.35 ± 8.03	69.83 ± 11.84
CXCR4% on CD4	10cm	82.69 ± 11.82	82.87 ± 7.31	77.52 ± 14.54	79.36 ± 13.78
	30cm	82.52 ± 12.17	81.75 ± 8.22	76.64 ± 14.89	76.31 ± 16.33
CCR5%/CXCR4% on CD4	10cm	58.07 ± 15.02	56.83 ± 9.92	52.11 ± 12.34	52.39 ± 11.47
	30cm	62.91 ± 12.62	61.69 ± 8.75	54.99 ± 13.60	53.44 ± 13.79

*The means and standard deviations reported in Table 2b are for specified visits only.
**One subject's (ID=417) baseline IL-1b measurements were extreme outliers and excluded from analysis.
