# Supplementary material for: First Phase 1 Double-Blind, Placebo-Controlled, Randomized Rectal Microbicide Trial Using UC781 Gel with a Novel Index of Ex Vivo Efficacy
Source: PLoS One. 2011 Sep 28;6(9):e23243. doi: 10.1371/journal.pone.0023243 (PMC3182160; doi:10.1371/journal.pone.0023243)
Supplement: Protocol S1 — Trial Protocol. (PDF) [file pone.0023243.s004.pdf]

U-19 Microbicide Development Project

Project 4

A Phase I Randomized, Blinded, Placebo-Controlled Safety and Acceptability Study of the UC-781 Vaginal Microbicide Gel Formulation Applied Rectally in HIV-1 Seronegative Adults

Amendment #1

Sponsored by:

CONRAD
Arlington, VA

In Partnership with:

Division of AIDS, US National Institute of Allergy and Infectious Diseases
US National Institutes of Health

Principal Investigator:
Peter Anton, MD
University of California, Los Angeles
Los Angeles, CA USA

Version 7.0
05 April 2007

Clarification Memo – January 13, 2008
Table of Contents updates – January 13, 2008
Protocol Team Roster update – January 22, 2008

MDP Project 4

A Phase I Randomized, Blinded, Placebo-Controlled Safety and Acceptability Study of the UC-781 Vaginal Microbicide Gel Formulation Applied Rectally in HIV-1 Seronegative Adults

TABLE OF CONTENTS

LIST OF ABBREVIATIONS AND ACRONYMS iv

PROTOCOL TEAM ROSTER v

SCHEMA viii

1.0 INTRODUCTION 1

1.1 Background of Microbicide Research 1
1.2 Background of UC-781 3
1.2.1 *In vitro* and *Ex vivo* Research 3
1.2.2 Animal Studies 4
1.2.2.1 Bioavailability Study Data 4
1.2.2.2 Reproduction and Teratology 4
1.2.2.3 Acute Toxicity Study Data 4
1.2.2.4 Repeated Dose Toxicity Study Data 5
1.2.2.5 Irritation Study Data 5
1.2.2.6 Safety Data in 0.1% and 1.0% UC 781 in Pigtailed Macaque 6
1.2.3 Clinical Research 8
1.3 Rationale 11

2.0 STUDY OBJECTIVES AND DESIGN 11

2.1 Objectives 11
2.1.1 Primary Objective 11
2.1.2 Secondary Objective 13
2.1.3 Tertiary Objective 16
2.2 Study Design 17

3.0 STUDY POPULATION 18

3.1 Inclusion Criteria 18
3.2 Exclusion Criteria 19
3.3 Recruitment Process 20
3.4 Participant Retention 20
3.5 Participant Withdrawal 21

4.0 STUDY PRODUCT CONSIDERATIONS 21

4.1 Drug Characteristics 21

4.2 Study Product 21
4.3 Product Supply and Distribution 22
4.4 Dose Regimen 22
4.5 Product Accountability 22
4.6 Adherence Assessment 22
4.6.1 Definition and Management of Non-Adherence 23
4.7 Concomitant Medications 23

5.0 STUDY PROCEDURES 23

5.1 Visit 1 – Screening 23
5.1.1 Non-Clinical Procedures 24
5.1.2 Clinical Procedures 24
5.1.3 Laboratory Procedures 24
5.2 Visit 2 - Enrollment 25
5.2.1 Non-Clinical Procedures 25
5.2.2 Clinical Procedures 25
5.2.3 Laboratory Procedures 26
5.3 Visit 3 - Single Dose Administration 26
5.3.1 Non-Clinical Procedures 26
5.3.2 Clinical Procedures 26
5.3.3 Laboratory Procedures 27
5.4 Treatment Stage 1 - Follow-up Period 27
5.5 Visit 4 – Treatment Stage 2 Safety Clearance 27
5.5.1 Non-Clinical Procedures 27
5.5.2 Clinical Procedures 28
5.5.3 Laboratory Procedures 28
5.6 Treatment Stage 2 – Outpatient 28
5.7 Visit 5 - Treatment Stage 2 Follow-Up Evaluation 28
5.7.1 Non-Clinical Procedures 29
5.7.2 Clinical Procedures 29
5.7.3 Laboratory Procedures 29
5.8 Treatment Stage 2 - Follow-Up Period 29
5.9 Visit 6 - In-Depth Interview 30

6.0 SAFETY MONITORING AND ADVERSE EVENT REPORTING 30

6.1 Safety Monitoring 30
6.2 Adverse Event Reporting Requirements 30
6.3 Adverse Experience Reporting Requirements 32
6.4 Expedited Adverse Event (EAE) Reporting to DAIDS 33
6.4.1 EAE Reporting Requirements for this Study 33
6.4.2 Timetable for Reporting Expedited Adverse Events 34

7.0 STATISTICAL CONSIDERATIONS 34

7.1 Review of Study Design 34
7.2 Randomization of Treatment Assignments 35
7.3 Unblinding 35
7.3.1 Emergency Unblinding Procedures 35
7.4 Statistical Procedures for Analysis of Safety Data 35
7.5 Statistical Procedures for Analysis of Acceptability Data 35
7.6 Statistical Procedures for Analysis of Mucosal Damage Parameters 36

7.7	Statistical Procedures for Rectal Microflora Studies	38
7.8	Statistical Procedures for Explant Studies	38
7.9	Statistical Procedures for Analysis of Pharmacokinetic Data	38
7.10	Statistical Procedures for Missing Data	38

8.0 HUMAN SUBJECTS CONSIDERATIONS 39

8.1	Ethical Review	39
8.2	Informed Consent	39
8.3	Risks	40
8.4	Benefits	42
8.5	Incentives	42
8.6	Confidentiality	42
8.7	Communicable Disease Reporting Requirements	42
8.8	Study Discontinuation	42

9.0 LABORATORY SPECIMENS AND BIOHAZARD CONTAINMENT 43

9.1	Local Laboratory Specimens	43
9.2	Outside Laboratory Specimens	43
9.3	Specimen Storage	43
9.4	Biohazard Containment	44

10.0 ADMINISTRATIVE PROCEDURES 44

10.1	Study Activation	44
10.2	Study Coordination	44
10.3	Study Monitoring	44
10.4	Protocol Compliance	45
10.5	Investigator's Records	45
10.6	Use of Information and Publications	45

11.0 INVESTIGATOR AGREEMENT 46

12.0 REFERENCES 47

APPENDICES

**Appendix I:**	Schedule of Study Visits and Procedures
**Appendix II:**	HIV Testing Algorithm
**Appendix III:**	Histopathology Scoring System
**Appendix IV:**	DAIDS Rectal Toxicity Table
**Appendix V:**	DAIDS Toxicity Tables
**Appendix VIa:**	Product Acceptability Assessment- Female Participants
**Appendix VIb:**	Product Acceptability Assessment- Male Participants
**Appendix VII:**	Sample Informed Consent
**Appendix VIII:**	Sample Participant Instructions
**Appendix IX:**	Sample Product Use Log
**Appendix X:**	CDC STD Treatment Guidelines
**Appendix XI:**	Normosol-R pH7.4 Package Insert

MDP Project 4

A Phase I Randomized, Blinded, Placebo-Controlled Safety and Acceptability Study of the UC-781 Vaginal Microbicide Gel Formulation Applied Rectally in HIV-1 Seronegative Adults

LIST OF ABBREVIATIONS AND ACRONYMS

AE	Adverse experience/event
AIDS	Acquired Immunodeficiency Syndrome
BBQ	Baseline Behavioral Questionnaire
BID	Twice daily (i.e. morning and night)
CASI	Computer Assisted Self-Interview
CCR5	Chemokine receptor 5
CDC	Centers for Disease Control and Prevention
CLIA	Clinical Laboratory Improvement Amendments
CORE	Coordinating and Operations Center
CPR	UCLA Center for Prevention Research
CRF	Case report form
CXCR4	CXC chemokine receptor 4
DAIDS	Division of AIDS
EC	Ethics Committee
ELISA	Enzyme-Linked Immunosorbent Assay
EAE	Expedited Adverse Event
FDA	(United States) Food and Drug Administration
GALT	Gut-associated lymphoid tissue
GCP	Good Clinical Practices
HIV	Human Immunodeficiency Virus
IRB	Institutional Review Board
LL	Local Laboratory
MMC	Mucosal Mononuclear Cells
MSM	Men who have Sex with Men
N-9	Nonoxynol-9
NIAID	(United States) National Institute of Allergy and Infectious Diseases
NIH	(United States) National Institutes of Health
PAQ	Product Acceptability Questionnaire
PBMC	Peripheral blood mononuclear cells
PCR	Polymerase Chain Reaction
PID	Participant Identification Number
PRN	As needed
QD	Daily
RANTES	Regulated on activation normal T cell expressed and secreted
RCC	Regulatory Compliance Center
RAI	Receptive Anal Intercourse (refers to coitus only, does not include manual stimulation or the use of sex toys or purgatives)
RT	Reverse transcriptase
SAE	Serious Adverse Experience
SADR	Suspected Adverse Drug Reaction
SIV	Simian Immunodeficiency Virus
SMC	Safety Monitoring Committee
SSP	Study-specific procedures
STD/STI	Sexually Transmitted Disease/Infection
UAI	Unprotected Anal Intercourse
UCLA	University of California, Los Angeles

MDP Project 4

A Phase I Randomized, Blinded, Placebo-Controlled Safety and Acceptability Study of the UC-781 Vaginal Microbicide Gel Formulation Applied Rectally in HIV-1 Seronegative Adults

PROTOCOL TEAM ROSTER

ALPHABETICAL ORDER

Peter Anton, MD
MDP Program Director
Principal Investigator
UCLA Center for Prevention Research
10940 Wilshire Blvd., Suite 1250
Los Angeles, CA 90024 US

Telephone: 310-206-5797
Fax: 310-206-8824
Email: PAnton@mednet.ucla.edu

Marianne Morse Callahan
Deputy Director/Clinical Director
CONRAD
611 North Kent Street, Suite 806
Arlington, VA 22209

Telephone: 703-276-3915
Fax: 703-524-4770
Email: mcallahan@conrad.org

Alex Carballo-Dieguez, PhD
Consultant
749 West End Ave
Suite 12-W
New York, New York 10025

Telephone: (212) 666-6686
Email: carballodieguez@aol.com

Daniel Cho, MD
Sub-Investigator
UCLA Center for Prevention Research
675 Charles E. Young Drive South, MRL 1240
Los Angeles, California, 90095 US

Telephone: 310-794-7195
Fax: 310-206-0279
Email: dcho@mednet.ucla.edu

Grace Chow, MS
Biologist
Prevention Research Branch
Division of AIDS
6700 B Rockledge Drive, Room 5119
Bethesda, MD 20892-7628

Tel: 301.435.3747
Fax: 301.402.3684
E-Mail: GChow@niaid.nih.gov

Elena Khanukhova
Clinical Research Manager
MDP Regulatory Core
UCLA Center for Prevention Research
10940 Wilshire Blvd., Suite 1250
Los Angeles, CA 90024 US

Telephone: 310-206-3633
Fax: 310-794-2829
Email: ekhanukhova@mednet.ucla.edu

Christine Mauck, MD, MPH
Sr. Medical Advisor
CONRAD
611 North Kent Street, Suite 806
Arlington, VA 22209

Telephone: 703-276-3912
Fax: 703-524-4770
Email: cmauck@conrad.org

Ian McGowan, MD, PhD
Director MDP Regulatory Core
Consultant
University of Pittsburgh School of Medicine
Magee-Women's Research Institute
204 Craft Avenue
Room B505
Pittsburgh, PA 15213

Telephone: 412-641-4710
Fax: 412-641-6170
Email: mcgowanim@mail.magee.edu

Jeanna Piper, MD
DAIDS Medical Officer
Microbicide Research Branch
Division of AIDS
6700 B Rockledge Drive, Room 5248
Bethesda, MD 20892

Telephone: 301-451-2778
Fax: 301-402-3684
Email: piperj@niaid.nih.gov

Jim A. Turpin, PhD
DAIDS Representative
Microbicide Research Branch
Division of AIDS
6700 B Rockledge Drive, Room 4115
Bethesda, MD 20892

Telephone: 301-451-2732
Fax: 301-496-8530
Email: JTurpin@niaid.nih.gov

Ana Vetuneac, PhD Candidate
Consultant
8715 81st Avenue
Glendale, NY 11385

Telephone: 718-805-2861
Email: amvccny@aol.com

Tape Transcription Center
129 Tremont Street
Boston, MA 02108

Telephone: 617-423-2151
Fax: 617-695-0631
Email: contact@ttctranscriptions.com
Website: www.TTCTranscriptions.com

MDP Project 4

A Phase I Randomized, Blinded, Placebo-Controlled Safety and Acceptability Study of the UC-781 Vaginal Microbicide Gel Formulation Applied Rectally in HIV-1 Seronegative Adults

SCHEMA

Purpose: The purpose of this study is to obtain preliminary data on the safety and acceptability of UC-781 vaginal microbicide gel (0.1% and 0.25%) versus placebo when rectally administered in HIV-1 seronegative adults.

Design:

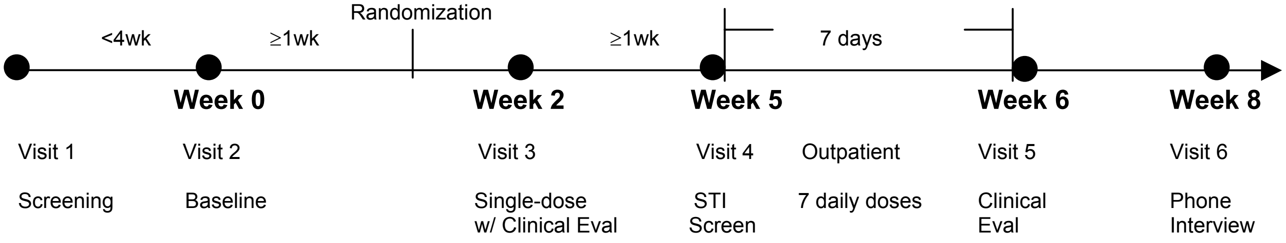

Participants will enter a screening period, which includes Visit 1 and if necessary a medication washout period.

Subjects who are deemed eligible after this screening period will return for a baseline evaluation (Visit 2), including clinical evaluation and mucosal specimen collection, as well as the Baseline Behavioral Questionnaire (BBQ). The BBQ will be administered by Computer Assisted Self-Interview (CASI). Subjects may complete the web-based CASI in the privacy of their own home. If a subject does not have access to the Internet, prefers to complete the BBQ at the study site, or requires assistance in using the CASI, he or she will be accommodated at the study site.

After completing Visits 1 and 2, eligible subjects will be randomized to one of three groups: 0.1% UC-781 gel, 0.25% UC-781 gel, or a placebo gel (12 per group). A subset of 9 subjects (3 from each group) will also participate in a pK sub-study, which will include 6 timepoints (Visit 3-pre, 0.25 hr, 2 hr, 4 hr, Visit 3A- 24 hr, and Visit 5- 1 day post-QD dose). During randomization, study staff will be notified if the participant is randomized to the pK substudy.

This study will involve 2 stages of treatment. **Each treatment stage is independent of the other. This study aims to examine the effects of 2 different dosing regimens, NOT cumulative safety over the both stages.**

Once randomized, subjects will return to the clinic (Visit 3), where a single dose of the study gel (Treatment Stage 1) will be administered after a preparatory Normosol-R pH7.4 enema. Within approximately 30 minutes, lavage, stool, sponge, and colorectal biopsy specimens will be obtained. Subjects will receive a safety follow-up phone call.

Subjects will return to the clinic for Treatment Stage 2 Clearance (Visit 4), in the week prior to beginning Treatment Stage 2. If given clearance, the subject may begin Treatment Stage 2.

In Treatment Stage 2, subjects will begin to self-administer once daily outpatient doses of the study gel for 7 days. Subjects will receive a follow-up safety phone call mid-way through

Stage 2. After completion of Stage 2 the subject will complete a Product Acceptability Questionnaire (PAQ) by CASI and return to clinic for evaluation and specimen collection (Visit 5). Subjects will receive a safety follow-up phone call.

After completion of Visit 5, subjects will be contacted by an interviewer to complete an in-depth phone interview regarding product acceptability. [The complete results of these interviews will not be shared with the rest of the research staff until the blind has been broken, as subjects may unwittingly reveal information that would break the blind. Only preliminary data that does not compromise the blind may be shared prior to data lockdown and unblinding.]

PRN visits

- Any complications that arise may require further clinical evaluation.

Study Population: HIV negative men and women with a history of RAI *
**Refers to coitus only, does not include manual stimulation or the use of sex toys or purgatives.*

Study Size: A total of 36 participants (12 per treatment group).
A subset of 9 participants will be assigned to the pK study.

**Treatment Regimen:**	Treatment Stage 1- Study gel administered once	Clinic
	Full 7-day recovery period	
	**Up to 3 weeks later:**	
	Treatment Stage 2- Study gel QD x 7 days	Outpatient

Study Duration: Participant accrual will take approximately 9-12 months and each participant will be on study for approximately 7-14 weeks. The total duration of the study will be approximately 18 months. Specimen and data analysis will require an additional four months.

Primary Objective: To evaluate the safety and acceptability of 0.1% and 0.25% UC-781 vaginal microbicide gel versus placebo when applied rectally.

Parameters to be studied include:
- Frequency of ≥ Grade 2 adverse events
- Acceptability assessments

Secondary Objective: To determine whether use of study product is associated with rectal mucosal damage characterized by the following:

Parameters to be studied include:
- Epithelial sloughing
- Histopathology
- Microflora
- Mucosal mononuclear cell phenotype
- Mucosal cytokine profile
- Mucosal immunoglobulins
- Fecal calprotectin
- Explants- Mucosal cytokine profile and susceptibility to HIV infection

Tertiary Objective: To determine the pharmacokinetics of UC-781 vaginal microbicide gel administered rectally in a subset of participants.

Parameters to be studied include:
UC-781 blood levels to determine absorption from the GI tract

1.0 INTRODUCTION

This study (Project 4) is one of three projects comprising the NIH/NIAID U-19 Microbicide Development Program (MDP), which seeks to advance the microbicide field by partnering with industry to create a research pipeline for candidate microbicides. Whereas the other MDP projects relate to the pre-clinical pipeline and behavioral components, Project 4 moves the MDP in to the clinical pipeline. Specifically, Project 4 seeks to achieve the aims of the MDP, by establishing the rectal safety and acceptability profile for UC-781 (CONRAD).

UC-781 is currently being evaluated in a number of clinical trials for the vaginal compartment and is a lead candidate for a successful microbicide. As cited below, often products approved for vaginal use are used off-label in the rectal compartment. As such, the responsible course of action is to, in parallel with vaginal trials, evaluate the safety of UC-781 for a rectal indication.

1.1 Background of Microbicide Research

Context of Receptive Anal Intercourse
The prevalence of receptive anal intercourse (RAI) in both men who have sex with men (MSM) and heterosexuals has only recently been elucidated. The practice of RAI may be used as a method of contraception, preserving virginity, or for pleasure. Pleasure can be explained by the close association of gut signaling pathways with genitourinary signaling pathways[6,7,8,9,10]. Several large surveys of sexual behavior indicate that RAI is part of the heterosexual repertoire and, alarmingly, much of heterosexual RAI is unprotected[11,12,13].

Population	N	Prevalence of AI	Reference
MSM in EXPLORE study	4295	48 – 54%	Koblin et al. 2003[5]
High risk women	1268	32%	Gross M et al. 2000[3]
College students	210	20%	Civic D 2000[2]
US Survey 15 – 44 years	12,571	35-40%	CDC 2005[1]
Californian residents	3545	6-8%	Erickson PI et al. 1995[4]

In 1992, the National Health and Social Life Survey (NHSLS)[11] approximately 20% of heterosexual women stated that they had a least one lifetime experience of RAI, 8% in the last year. Four years later, the National Sexual Health Survey (NSHS)[12] found similar rates and of those practicing RAI, 80% did not use condoms. This is in contrast to the Gay Urban Men's Health Study (GUMS)[13], which found that 50% of MSM reported a history of anal intercourse in the past year, with higher rates of condoms use (approximately two-thirds). All three of these studies may grossly underestimate rates of RAI. Each study was conducted by interview. Other studies have shown that subjects are more likely to report engaging in RAI when taking a computer-assisted self-interview (CASI) than when asked by an interviewer[14,15,16,17,18]. This is not unexpected given the sensitive nature of the topic.

Even less is known about the prevalence of RAI in the global community. However, in one multi-national trial of female sex workers in 4 developing countries in Asia and Africa, 14% of the women reported a lifetime history of RAI with a median frequency of 2 times a week[19].

Physiology of Risk

Receptive anal intercourse (RAI) confers a substantially higher risk (2-3 fold) of acquiring HIV than vaginal intercourse [20]. The gastrointestinal tract is the body's largest lymphoid organ, containing significant numbers of activated, memory T lymphocytes making it a prime site for HIV infection and amplification[21,22].

The rectal and vaginal epithelia differ significantly in their capacity to resist the traumatic friction accompanying sexual intercourse and in the immune parameters activated to provide protection against invasive pathogens. The rectum is lined by a single cell columnar epithelium scattered with activated intra-epithelial lymphocytes. The lamina propria is immediately subjacent and separated from the lumen by a single cell layer attached to the basement membrane by reticular fibers, increasing the likelihood of systemic exposure. The vaginal epithelium, in contrast, is a stratified epithelium with a low density of intraepithelial lymphocytes. The endo-cervical epithelium is more akin to that of the rectum with significant numbers of activated lymphocytes whose density fluctuates with hormonal cycles[23].

It remains unclear which factors may augment or reduce the risk of HIV transmission via the rectal route. The vulnerability of the rectal epithelium to the trauma of sexual intercourse suggests that the sub-epithelial mucosa may frequently be exposed to infected luminal ejaculate and, therefore, to the risk of systemic infection. The implications are that one cannot rely simply on epithelial integrity and focus on strategies associated with luminal absorption of infectious agents. Efforts must also be directed towards containment of potentially inflammatory sequelae that may favor HIV transmission.

Developing Safety Standards for Rectally-Administered Microbicides

Methods of assessing mucosal parameters characterizing any insult to the rectal mucosa that might guide the development of future rectal microbicides have not been established. Many safety risks may be subtle and require alternate modes of detection (PCR, flow cytometry, immunohistochemistry, etc). For this reason, the HIV Prevention Trial Network sponsored the HPTN 056 study "Characterization of Baseline Mucosal Indices of Injury and Inflammation in Men For Use in Rectal Microbicide Trials" conducted at UCLA. The lessons learned from the HPTN 056 trial have guided the selection of parameters to be included in Project 4. As these are assays in development and clinical relevance remains to be defined, these will not be safety indices but secondary endpoints. The rationale for selecting each of these endpoints is further described in Section 2.1.2.

Developing Standards of Acceptability for Rectally-Administered Microbicides

Prevention tools are effective only if used. The limited use of condoms by many at-risk individuals illustrates the importance of a product's acceptability, i.e., the willingness of the users of the product to employ it correctly and consistently. This study will explore UC-781 acceptability for rectal use by means of an assessment that uses both quantitative and qualitative methods. These assessments will evaluate not only acceptability of product, applicator, and application method characteristics but also the contextual circumstances in which the product is used by each participant and how such circumstances may affect sustained use in the future. The acceptability assessment (included in the appendix) consists of three elements: 1) Baseline Behavioral Questionnaire, 2) Product Acceptability Questionnaire, and 3) In-depth Acceptability Interview. The questionnaires (see Appendices VIa and VIb) were developed based on in-depth qualitative interviews of 20 participants in the first phase of R01 HD046060 "Topical Microbicide Acceptability," (Carballo-Diéguez, PI), a study currently underway that focuses on acceptability of rectal microbicides among men and women. At the time of this writing, the questionnaires have been administered to 100 MSM in Boston and New York, there being no comprehension problems or other difficulties with them. There are additional advantages to using the same product-acceptability instrument across studies, since this allows more valid post-hoc comparisons. The

questionnaire used in the R01 study has been adapted for female participants in this study (see Appendix VIa). Information gathered from female participants in this study will be used to design future versions of microbicide acceptability questionnaires.

1.2 Background of UC-781

The primary purpose of microbicides is to prevent transmission of sexually transmitted infections (STIs), including HIV, from an infected subject to an uninfected subject. The microbicide, UC-781[24], contains reverse transcriptase (RT) inhibitor antiviral capabilities that may reduce HIV infections.

Traditionally, RT inhibitors have been administered orally as in combination with antiretroviral regimens in subjects with established HIV infection. However, utilization of RT inhibitor microbicides may prevent local mucosal viral replication and abort HIV infection.

UC-781 (CONRAD) is a non-nucleoside reverse transcriptase inhibitor (NNRTI) of the HIV-1 reverse transcriptase (RT) enzyme. Since RT is essential to the replication of HIV-1, UC-781 prevents production of infectious virus. UC-781 displays nanomolar range EC 50 activity against wild-type HIV-1 virus and little to no cytotoxic effects on mammalian cells in culture. UC-781 functions as a tight-binder to HIV-1 RT[25,26]. This makes it an attractive microbicide candidate.

Pre-clinical development of UC-781 has been undertaken following the principles outlined by Lard-Whiteford et al.[27].

1.2.1 *In vitro* and *Ex vivo* Research

UC-781 is a potent NNRTI against a wide range of HIV-1 isolates. The EC50 values against HIV-1 isolates were less than 0.010uM[28]. Like other NNRTIs, UC-781 is not active against HIV-2 isolates, HIV-1 type O isolates, bacteria, yeast or fungi. The antiviral effects of UC-781 appear to have a long half-life. When uninfected lymphoid cells were pretreated with UC-781 for 18 hours, infection was dramatically reduced[29]. UC-781 also exhibited an inhibitory effect against chronically infected HIV-1 cells when UC-781 maintained inhibitory activity against HIV-1 infection *in vitro* and in an *ex vivo* model utilizing cervical explant tissue[30].

UC-781 suppresses HIV-1 production in cell culture at lower concentrations than other NNRTIs, and UC-781 is active against viruses with resistance to other NNRTIs, including nevirapine and delavirdine. And unlike other NNRTIs which lose activity with a single mutation, UC-781 requires multiple specific mutations for loss of activity[31].

The effects of UC-781 on mammalian cells have been studied *in vitro* and *ex vivo*. Toxicity studies of UC-781 gel formulations at 0.1% and 1% concentrations on epithelial cell lines showed no toxicity over 5 days of exposure. Similar studies in chronically infected HIV-1 T cells and non-infected peripheral blood mononuclear cells showed no effect of UC-781 of cell viability up to the maximum tested dose (1000nM)[32].

1.2.2 Animal Studies

1.2.2.1 Bioavailability study data:

Pharmacokinetic studies in multiple animal species (mice, rat, rabbit, dog and monkey) indicate that UC-781 has generally low oral bioavailability. UC-781 was below the level of detection in rabbit or dog plasma following intravaginal administration at multiples of 13-fold (rabbit) to 41-fold (dog), the maximum anticipated human equivalent dose[32].

1.2.2.2 Reproduction and Teratology

Segment I (fertility and general reproduction) Results: A Segment I study of UC-781 was conducted in rats receiving orally administered UC-781. Prior to mating doses of 0 mg/kg/day, 25 mg/kg/day, 100 mg/kg/day, 400 mg/kg/day were administered to 25 paternal and 25 maternal rats per dosing group. The final report is pending.

Segment II (embryo-fetal development) test results: Both a Segment II oral dosing study in rats and an intravaginal dosing study in rabbits were performed.

In the Segment II rat study the same doses were used (0 mg/kg/day, 25 mg/kg/day, 100 mg/kg/day, 400 mg/kg/day). Twenty-five pregnant rats per group were given daily doses from gestation day 7 to gestation day 17. The percentage of fetuses with any alteration per litter was 0.6%, 2.4%, 1.2% and 1.6% for the four dose groups respectively. Therefore, a no-observable-adverse-effect-level (NOAEL) for general toxicity of UC-781 was determined.

In the Segment II rabbit study, rabbits were dosed with the UC-781 carbomer gel formulation at doses of up to 26 mg/kg/day for 14 days. During the course of this study, 12 of 107 rabbits died sporadically between the 1st and 9th doses. No further mortality was observed after the 9th dose. Signs of toxicity were only observed immediately prior to death. In addition, there were no signs of toxicity after the preceding dose in the cases where repeat doses were administered. Blood drug levels were below the limits of detection after the 1st dose on gestation day 7 and after the last dose on gestation day 20.

In order to follow-up on concerns over the mortality, the Segment II rabbit study was repeated. No deaths were observed during the dosing period in this second study.

No drug related fetal toxic or teratological findings were observed in either rabbit study.

Two consulting toxicologists were asked to review the studies and give expert opinions regarding the rabbit mortality and the difference between the two studies. Both toxicologists concluded that the mortality observed in the first study was most likely due to the handling and dosing procedure and not a direct toxic effect of UC-781. The expert opinions have been filed with the UC-781 IND.

1.2.2.3 Acute toxicity study data

During preclinical animal studies, UC-781 was administered at high multiples (up to 345-fold) of the anticipated human exposure without significant toxicology. There was no evidence of systemic toxicity to UC-781 during single dose pharmacokinetic or dose ranging studies in rats (oral and intravaginal administration) and monkeys (oral, subcutaneous and intravenous administration)[32].

1.2.2.4 Repeated dose toxicity data

In the repeated dose toxicity studies, there was little evidence of systemic toxicity; any incidences of local irritation are also below and in the Investigator's Brochure[32].

Oral dosing

5-day rat-<br>3/ grp, 6 groups<br>(up to 1200 mg/kg/d)	Mild transient elevations in BUN, not associated with elevated creatinine.
28-day rat-<br>10/ grp, 4 groups<br>(up tp 400 mg/kg/d)	Statistically significant difference in liver weight for the 2 highest dose groups (100mg/kg/day and 400mg/kg/day) compared to controls, but these were not associated with microscopic liver changes.

Intravaginal dosing

7-day rat-<br>5/ grp, 4 groups<br>(up to 110 mg/kg/d)	No toxicity
28-day dog-<br>3/ grp, 4 groups<br>(up to 50 mg/kg/d)	No systemic toxicity.<br>Local irritation-<br>Erythema (3/3 in the mid and high dose UC-781 groups)<br>Discharge (all UC-781 groups)<br>Edema, severe (2/3 in the high dose group)<br>Histopathology: atrophy, inflammation were actually observed with less frequency in the mid and high dose UC-781 groups than in low dose UC-781 and control groups.

Rectal dosing

28-day rat-	Results pending
28-day rabbit-<br>4/ grp, 6 groups<br>(up tp 2.5% gel)	No systemic toxicity<br>Final report pending

In addition, UC-781 showed no genotoxicity as determined in three standard test systems (Ames test, *in vitro* Chinese Hamster Ovary cells, and *in vivo* micronucleus test in mice.

1.2.2.5 Irritation study data:

Vaginal[32]

In a 10-day rabbit vaginal irritation study, a 1% UC-781 gel formulation (equivalent to 3.33 mg/kg/day), a vehicle gel, or a 0.9% saline control was applied vaginally. A single observation of external vaginal erythema (scored as "Red" on grading system) was noted in 1/10 rabbits dosed with the 1% UC-781 gel, 1/10 rabbits dosed with the vehicle gel, and none of the rabbits dosed with the saline control. The erythema was not observed at any other timepoints in any of the groups. Histopathology results indicated minimal to moderate mixed inflammatory cell infiltrations, exudates, and edema in both the treated and control groups. The inflammatory response (mixed inflammatory cell infiltrations and exudates) was considered to have been due to repeated manipulation of the vagina since the responses in saline were increased in severity and incidence when compared to all other dose groups.

Penile[32]

In a 7-day penile irritation study in rabbits a 1% UC-781 gel formulation, 3% UC-781 gel formulation, vehicle gel, or a 0.9% saline control was applied topically to the penis. Rabbits were treated twice daily for seven consecutive days. The test article, vehicle, or saline control was applied with a syringe to the mucosal area beneath the sheath and spread evenly over the penis with a glass rod. The two daily doses were separated by five to six hours. At approximately the same times each day, rabbits were collared (for about two hours after each dose) to prevent removal of the test materials via grooming. Results from this study indicated that UC-781 did not produce penile irritation at concentrations up to 3%, a dose level higher than that anticipated to be used during Phase 1 clinical trials.

Sensitization[32]
UC-781 was shown to be a mild sensitizer in a guinea pig sensitization study. The objective of this study was to evaluate the potential of UC-781 to cause or elicit skin sensitization reactions (allergic contact dermatitis) via topical applications. The UC-781 carbomer gel formulations (1%, 3%, and 10%) were administered once during a dose range-finding screen. Based on results from the range-finding screen, the vehicle control gel, a positive control (100%-α-hexylcinnamaldehyde), and 10% UC-781 carbomer gel formulation were administered once weekly for three weeks during the induction phase. Following a two-week washout period, the groups began the challenge phase of the study which consisted of a single dose of test article. The positive control group from the induction phase received a 50%-α-hexylcinnamaldehyde in mineral oil during the challenge phase. Both the vehicle control and UC-781 groups from the induction phase received the 10% UC-781 carbomer gel during the challenge phase. A sensitization response was defined as a reaction in the UC-781 group greater than those seen in the vehicle or positive control gel animals at the challenge sites. The results indicated that 10% UC-781 carbomer gel is a mild sensitizer (2/20 animals had a score greater than 0.5).

1.2.2.6 Safety study data in 0.1% and 1.0% UC 781 in pigtailed macaque:

Vaginal[3233]
Two separate studies were undertaken: 1) 0.1% UC-781 vs placebo and 2) 1.0% UC-781 vs placebo. There were 6 animals in each treatment group. Each animal received 4 daily doses of study product. On day 5, there were no detectable levels of UC-781 in the serum of any of the animals. Colposcopic examinations of all animals were within the normal range. All pH measurements were within the normal range (4.0-8.0) for the vagina of the pigtailed macaque. However, 3/6 animals in the 0.1% UC-781 group had a transient drop in pH 30 minutes after gel application, but remained within the normal range.

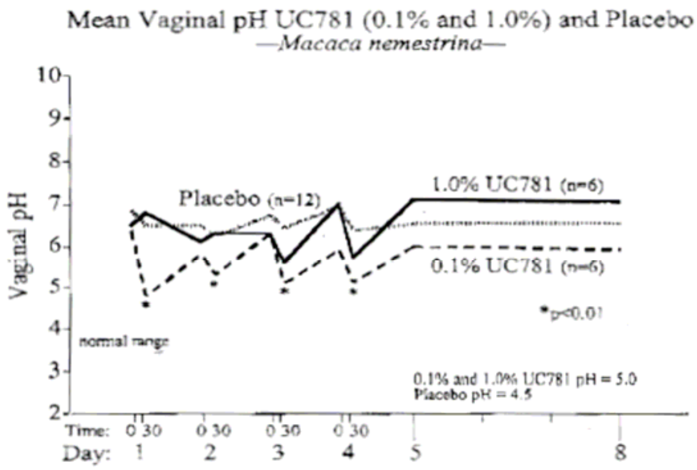

When comparing vaginal microflora among the three treatment groups, there were no significant differences in the prevalence of either H2O2 producing lactobacillus or viridans streptococci in any study arm. Likewise, when comparing prevalence of the H2O2 producing microorganisms within treatment groups, no significant differences occurred. Furthermore the only constituent of vaginal flora shown to be affected by UC-781 was black GNR. The quantity of black GNR grown from vaginal swab specimens decreased on days 3 and 4 ($p < 0.02$) after 0.1% UC-781 use. Following 1.0% UC-781 use, the quantity of black GNR decreased significantly ($p < 0.05$) on days 4 and 5. By day 8, the growth titers had rebounded and were similar to baseline levels in both treatment groups.

<u>Rectal</u>[32,33]

Two separate studies were undertaken: 1) 0.1% UC-781 vs placebo with a cross-over and 2) 1.0% UC-781 versus placebo with a crossover. There were 6 animals in each treatment group. Each animal received 4 daily doses of study product. On day 5, there were no detectable levels of UC-781 in the serum of any of the animals. All pH measurements were within the normal range (5.5-8.5) for the rectum of the pigtailed macaque. There were no significant differences between the two UC-781 test arms and the placebo regarding pre and post application pH. Within each treatment group, there were significant differences between pre and post-application rectal pH. Despite the acidic pH of the product and placebo, rectal pH increased significantly after application on all 4 days in the placebo group and on days 2 and 4 in the 0.1% UC-781 group. After 1.0% UC-781 rectal application a significant increase in pH was noted on day 3 only. Though there were transient pH shifts with product application, there were no significant differences between pH at baseline and follow-up in any group.

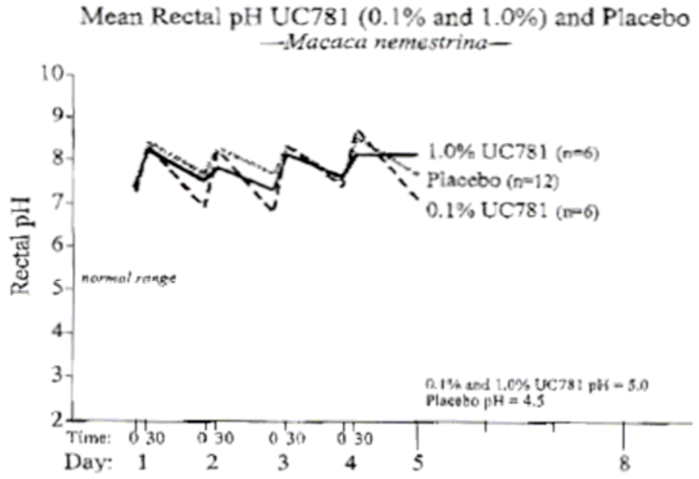

There was no statistically significant difference in the number of epithelial sheets or presence of blood/stroma in the rectal lavage specimens collected 2-4 hours after dosing.Semi-quantitative analysis of the prevalence of rectal microflora showed no significant differences in the prevalence of microflora when assessed daily, or pre and post-application of 0.1% or 1.0% UC781 or placebo. In addition, there were no significant differences in the prevalence of microflora at follow-up compared to baseline in either group. Rectal use of 0.1% or 1.0% UC781 did not significantly alter the presence of H2O2 producing Lactobacillus or viridans streptococci.

1.2.3 Clinical Research

The principles of clinical development drafted by Mauck et al[34] can be seen in the UC-781 clinical program.

A Phase I clinical trial to evaluate the safety and acceptability of UC-781 in healthy, sexually abstinent women was conducted in Virginia[32]. In this randomized, double-blind, placebo-controlled study, a total of 48 volunteers were enrolled at a single site and asked to apply placebo gel (N=12), 0.1% UC-781 (N=12), 0.25% UC-781 (N=12) and 1% UC-781 (N=12) intravaginally once per day for six days.

Subject participation in this first clinical study with UC-781 entitled, “Single and Multiple Exposure Tolerance Study of UC-781” (Protocol No. A02-083) occurred between August 2003 and March 2004. The study was designed to assess the safety and acceptability of three concentrations of UC-781 (0.1%, 0.25%, 1%) after a single dose and five additional consecutive daily doses administered to 12 women in each of the UC-781 dosage and placebo groups, a total of 48 women. Subjects were evaluated by pelvic exam with colposcopy after the first and sixth gel applications.

A total of 37 AEs were experienced by 8 (66.7%) participants in the placebo group, 5 (41.7%) in the 0.1% UC-781 group, 5 (41.7%) in the 0.25% UC-781 group and 4 (33.3%) in the 1.0% UC-781 group (Table 1). Only one of the eight moderate AEs was determined to have any relationship to a study product.

Table 1. Summary of Adverse Events from “Single and Multiple Exposure Tolerance Study of UC-781”

Adverse events * = genitourinary event CT= Concomitant Therapy

Participant #	Product Group	Adverse Event	Severity	CT	Relatedness	Duration	Timing
102	Placebo	Breakthrough bleeding*	Mild	Non – OCPs < 1 month	Unrelated	59 min	4th dose
109	Placebo	Pelvic cramping*	Mild	None	Possibly	5 min	5th dose
112	Placebo	Pelvic pain*	Mild	None	Unrelated	1 day	5th dose
		Eosinophil count ↑	Mild	None	Possibly	--	--
120	Placebo	Headache	Mild	Ibuprofen	Unrelated	1 hour 30 min	--
125	Placebo	Headache	Moderate	None	Unrelated	45 min	--
		Yeast infection*	Moderate	Diflucan	Possibly	ongoing	5th dose
137	Placebo	Peri-anal ulcer*	Moderate	Neosporin	Unrelated	ongoing	4th dose
139	Placebo	Genital pruritus* (vaginal)	Mild	None	Probably	1 hour	1st dose
		Pelvic cramping*	Mild	None	Possibly	30 min	1st dose
		Pelvic pressure*	Mild	None	Possibly	30 min	1st dose
		MCH ↑	Mild	None	Unrelated	--	--
148	Placebo	Genital pain (intermittent pain in cervix)*	Mild	None	Possibly	1 day	1st dose
101	0.1%	Vaginal discharge*	Mild	None	Definitely	1 min	4th dose
110	0.1%	Genital pruritus*	Mild	None	Definitely	2 hours 30 min	4th dose
		Eosinophils ↑	Mild	None	Unrelated	--	--
132	0.1%	Serum Amylase ↑	Mild	None	Unrelated	--	--
140	0.1%	Vaginal moisture*	Mild	None	Possibly	1 hour	6th dose
142	0.1%	Vulvovaginal Dryness*	Mild	None	Possibly	1 hour	6th dose
104	0.25%	Pelvic cramping*	Moderate	None	Unrelated	4 hours	4th dose
		Pelvic cramping*	Moderate	None	Unrelated	2 hours	5th dose
107	0.25%	Introital stinging*	Mild	None	Definitely	4 min	1st dose
		Introital stinging*	Mild	None	Definitely	3 min	2nd dose
		Monocytes ↓	Mild	None	Unrelated	--	--
114	0.25%	Lymphocytes ↑	Mild	None	Possibly	--	--
		Polys ↓	Mild	None	Possibly	--	--
134	0.25%	URI	Moderate	Nyquil	Unrelated	2 days	--
144	0.25%	Headache	Mild	None	Unrelated	3 hours 45 min	--
		Headache	Mild	None	Unrelated	1 hour 20 min	--
106	1.0%	Genital pruritus*	Mild	None	Unrelated	1 hour 20 min	4th dose
		Genital pruritus*	Mild	None	Definitely	1 min	6th dose
		Loss of appetite	Moderate	None	Unrelated	1 day	6th dose
		Nausea	Moderate	None	Unrelated	1 day	6th dose
118	1.0%	Monocytes ↓	Mild	None	Unrelated	--	--
		Headache	Mild	Tylenol	Unrelated	ongoing	--
124	1.0%	Vaginal discharge*	Mild	None	Definitely	ongoing	5th dose
130	1.0%	Headache	Mild	None	Unrelated	8 hours	--

Most of the AEs were graded as mild and none was graded as severe. There were 8 complaints in 5 participants that were graded as moderate.

- One patient in the placebo group complained of a headache of moderate severity and was diagnosed with a yeast infection of moderate severity that began on the 5th day of dosing for which she received diflucan.
- One patient in the placebo group complained of a peri-anal ulcer of moderate severity for which she applied topical Neosporin.
- One patient in the 0.25% group complained of 2 episodes of pelvic cramping of moderate severity as described above.
- One patient in the 0.25% group complained of a cold of moderate severity for which she took Nyquil.
- One patient in the 1.0% group complained of loss of appetite and nausea of moderate severity that lasted for 1 day on the 6th day of dosing.

Of the moderate AEs, only the yeast infection was felt to be probably related to the study product by the site study clinician.

Two subjects discontinued prematurely from the study, both in the 0.25% UC-781 group. One subject had a small cervical lesion determined to be an applicator injury. The other withdrew consent, unrelated to study product. Subjects were evaluated during a pelvic exam by colposcopy after the first and sixth gel applications. Twelve women each had 1 finding on colposcopy: 1 in the placebo group, 3 in the 0.1% group, 5 in the 0.25% group, and 3 in the 1% group. Only 1 woman had more than superficial epithelium changes, a woman in the 0.25% group with a deep epithelium lesion due to applicator injury.

Participants' plasma samples were analyzed for UC-781 at baseline and after 6 applications of placebo gel, 0.1%, 0.25%, or 1% UC-781 gel. The lower limit of quantification (LLOQ) of the LC-MS bioanalytical assay was 2.5 ng/mL. There were no detectable levels of UC-781 in samples after treatment with placebo gel or 0.1% and 0.25% UC-781 gels. After six daily applications, two participants who were randomized to the 1% UC-781 gel group had plasma levels that were detectable but less than the LLOQ.

The key acceptability item was whether or not the subject indicated that she would buy the product if she needed protection against HIV and if the product she used were effective. All of the participants indicated that they would buy the product if proved effective with only one exception in the 0.1% UC-781 group.

Another Phase I study conducted at Emory University was started in October 2005 in order to assess the safety and acceptability of vaginal use of 0.1% and 0.25% UC-781 gel in sexually active HIV-uninfected women, and their male partners, and sexually abstinent HIV-infected women. The population will consist of 36 sexually active HIV-uninfected women and up to 36 of their male partners; in addition to 18 sexually abstinent HIV-infected women

The study will proceed in two stages. In Stage 1, sexually active HIV-uninfected women will be randomized to apply 0.1% UC-781 Gel, 0.25% UC-781 Gel or Control Gel intravaginally twice daily for 14 days. If safety data from stage 1 is acceptable with no evidence of UC-781 systemic absorption, the study will proceed to Stage 2. In Stage 2, sexually abstinent HIV-infected women will be randomized to apply 0.25% UC-781 Gel or Control Gel intravaginally twice daily for 14 days.

This Phase I study will continue the clinical development pathway for UC-781, progressing from the initial 6-day study of multiple concentrations in sexually abstinent women to a 14 day, twice daily application study using 0.1% and 0.25% concentrations in sexually active women. Because, the potential for absorption is higher with the 1% formulation, this 14-day study will involve the 0.1% and 0.25% UC-781 gel formulations only.

Recruitment is anticipated to take 6 months. Currently, there are 3 subjects enrolled into the trial. No serious adverse events have been reported.

Two additional Phase I studies are being developed in Thailand and Botswana. These studies will continue to evaluate the safety, acceptability, and toxicity data necessary to develop UC-781.

1.3 Rationale

Past studies have shown that vaginal products are often used rectally. Developing a safety profile for rectal use of the UC-781 vaginal microbicide is the safest course of action. Successful demonstration of safe and acceptable use of the UC-781 vaginal gel formulation rectally may lead to further clinical development of UC-781 gel for rectal use.

The decision to pursue clinical development of a candidate UC-781 gel formulation depends on multiple measures of safety, pharmacokinetics, and acceptability. The rationale for each endpoint is described in more detail in Section 2.1.

In order to discover the potential effects of UC-781 formulated for vaginal use when administered rectally, the study design involves 2 treatment stages: Treatment Stage 1, single dose rectal application of study gel in the clinic; Treatment Stage 2, outpatient self-administered rectal application of the study gel once daily for 7 days. After each treatment phase, subjects will have intensive clinical assessments.

Healthy volunteers will be studied due to the confounding effect of existing GI pathology with active infections, including HIV. Adults with a history of receptive anal intercourse will be recruited as one important population intended for future use of the UC-781 rectal gel formulation. Past sexual experiences with RAI will put the acceptability assessments in context. This multi-treatment design will establish an ideal dose and dosing regimen to proceed with clinical development.

2.0 STUDY OBJECTIVES AND DESIGN

2.1 Objectives

2.1.1 Primary Objective

To evaluate the safety and acceptability of 0.1% and 0.25% UC-781 vaginal microbicide gel versus placebo when applied rectally.

Parameters to be studied include:

- Frequency of ≥Grade 2 adverse events

Rationale: Frequency of adverse events is always an objective in a Phase I study of an investigational product.

- Acceptability assessments

Rationale: The importance of determining acceptability in the early phases of microbicide development cannot be overstated. Making changes to a product earlier rather than later in development will reduce the total cost of drug development significantly and ultimately achieve the desired outcome, increased use of a product that would decrease the global burden of HIV.

In order to assess acceptability, questionnaires will be posted on a Web page that will be accessible only to study participants by entering a password. Web-based surveys have all the advantages of Computer Assisted Self Interview (CASI) over paper-and-pencil or interviewer-administered assessments, especially for sexual behavior. Advantages of using Web-based or CASI surveys include greater privacy and confidentiality, and increased likelihood that risky behaviors will be reported due to lack of self-presentation bias[14,15,16,17,18]. The experience of colleagues at the HIV Center for Clinical Studies, in New York City, with these technologies has been extremely positive, even with participants who had never used a computer. This

technology also allows data entry directly into an electronic file, thus minimizing coding and entry errors. Core C, the MDP Data Management and Biostatistics Core, will program the questionnaires for secure posting on the Web.

Baseline Behavioral Questionnaire (BBQ). Prior to Visit 3, participants will be asked to respond to the BBQ. The content of the BBQ and the rational for inclusion in the currently proposed study appear in Table 2.

Table 2. Baseline Behavioral Questionnaire (See Appendices VIa and VIb)

Section	Title	Content and rationale for inclusion of this section
A	Demographics	Age, educational level, ethnicity, gender identity, sexual identity, employment status and income will be used for sample description.
B	Sexual Behavior (prior 2 months)	For male participants: 1) habitual sexual behavior with HIV-negative, positive, or unknown status men and women, 2) sexual roles --insertive, receptive, versatile, and 3) frequency of condom use.<br><br>For female participants: 1) habitual sexual behavior with HIV-negative, positive, or unknown status men and women, 2) frequency of condom use.<br><br>This baseline data will allow us to understand the participant's habitual sexual behavior in which microbicide use needs to fit as well as the degree of sexual risk behavior that could potentially be curbed by using a microbicide.
C	Condom Norms	These items will evaluate perceived social norms concerning condom use.
D	Douching (prior 2 months)	Frequency of enema use, purpose, age of first use, enema use prior and after sex, type of douche apparatus used, commercial products, mode of application, location, position, injuries resulting from douching, and side effects. This will allow us to understand degree of similarity between habitual practices and study requirements, and how this may affect adherence to the protocol as well as future use of enemas (and enema types), if recommended.
E	Lubricant Use (prior 2 months)	Types of rectal lubricants (e.g., silicone, oil or water based) participant's use, preferences, amount used, and mode of application. This will allow us to consider possible preexisting preferences and how they may affect attitudes about UC781 gel.
F	Nonoxynol-9	Many individuals have used lubricants containing N-9 before knowing it could be harmful. We want to assess past use of N-9 and its impact on willingness to use microbicides.
G	Substance Use (prior 2 months)	To determine what recreational drugs the individual uses in conjunction with sex and how this may affect microbicide use. Viagra and other sexual enhancing drugs will also be included.
H	HIV Testing	By eligibility criteria, all participants will be HIV-negative. We will assess frequency of HIV testing and latest test occasion prior to enrollment in the study.
I	Rectal Microbicide Intentions	To assess intentions to use a microbicide gel and a preparatory enema before RAI. This will allow us to detect pre-trial attitudes about such products and how they may affect later ratings.
J	Pleasure	Severy, *et al.* [63, 65 2] stated that one of the main reasons people have sex is for pleasure. These items explore the effect of condoms on sexual pleasure. They will serve as baseline to compare pleasure ratings related to the different products used in the trials.

Product Acceptability Questionnaire (PAQ) The PAQ will be completed within the Visit 5 window after the completion of treatment stage 2, once the participant has had the opportunity to familiarize himself/herself with method of application, applicator, and product characteristics. The Product Acceptability Questionnaire includes both close-ended and open-ended questions (see Table 3)

Table 3: Product Acceptability Questionnaire (See Appendices VI a and VI b)

Section	Title:	Content
A	PRODUCT ACCEPTABILITY	Likes and dislikes of physical characteristics of the product and sensations
B	APPLICATION PROCESS	Ease of application, position, timing
C	APPLICATOR	Likes and dislikes about applicator, its tip, and its portability
D	CHANGES IN VOLUME USED	Any changes introduced by the user in the volume of product applied
E	EXPERIENCES USING THE PRODUCT	Leakage, soiling, or other product side effects, and how much the participant was bothered by them; feeling that the product was absorbed in the intestine
J	INTENTION TO USE PRODUCT IN THE FUTURE	Willingness to use a product that contributed to provide some protection against HIV; willingness to use it when condoms are not used, if wait period were required, and according to its cost.
K	WILLINGNESS TO USE HIGHER VOLUME	Attitudes about different product volumes
L	POSSIBILITY OF COVERT USE	Possibility of non-disclosure of product use to different types of partners
M	RECOMMENDATIONS	Suggested changes to the product

Once participants complete the PAQ, they will be asked to indicate a phone number and some convenient times to reach them for an in-depth phone interview.

In-depth Acceptability Interview. This is probably the richest part of the acceptability assessment. It will be conducted by one of two highly trained interviewers (either a male interviewer for a male participant, or a female interviewer for a female participant) with experience in rectal microbicide research. After carefully reviewing the BBQ and the PAQ, the interviewer will elicit a detailed narrative of the participants' experience using the product, especially the association (or lack of) between habitual hygiene, lubricant use, and sexual behaviors reported at baseline and those required or proscribed by the study protocol. The purpose of this interview is to capture the specific circumstances of each individual's use of the product, in the context of a relationship or lack of it, in different places, times, sexual scenarios, sexual partnerships, and within specific cultural frames. The goal is to understand the learning process that occurs in product use and the difficulties that arise, as well as to record nuances that are not captured in the structured assessments. The interview will be audio-taped and transcribed. A guide for in-depth questions is included at the end of the Acceptability Assessment included in the Appendix.

2.1.2 Secondary Objective

To determine whether use of study product is associated with rectal mucosal damage characterized by the following:

Parameters to be studied include:

- Epithelial Sloughing

Rationale: The sloughing assay is integral to the evaluation of microbicides in the rectum. The only microbicide previously studied in the rectum, is N-9, which led to rapid exfoliation of the rectal epithelium in mice[35] and variable abnormality in humans[36,37] A recent study has demonstrated that up to 41% of men who have sex with men (MSM) use vaginal products containing spermicides for lubrication during rectal sex[38]. It is important to have confidence that any microbicide going to market will not have a similar effect.

Using the N-9 example, it is expected that evidence of any injury may be apparent within 15 minutes of exposure to an offending agent[36]. In the case of N-9, substantial reconstitution of these changes occurred by 2 hours and microscopically normal

epithelium was noted after 24 hours[39,37,36]. The observation of epithelial sloughing was made by performing a rectal lavage and examining the effluent for epithelial cells. Measurements were conducted using either microscopy, looking for sheets of epithelia, or by counting the cells with a Coulter Counter.

Given the above information, it is best to obtain lavage specimens within the 15 minute –2 hour window after application of a product rectally. Therefore, in this study the first treatment stage will include rectal lavage in order to monitor subjects for epithelial sloughing. Although presence of some epithelial cells in effluent is normal, a dramatic increase from baseline would be concerning. In order to avoid the confounding effects of the standard pre-sigmoidoscopy prep (a hypertonic enema), a Normosol-R pH7.4 (See Appendix XI for package insert) enema will be used instead. Rectal lavage will be performed during the endoscopy at approximately 5 cm. Careful attention will be paid to perform the second lavage at 5 cm to decrease the likelihood of washing the UC-781 off of the mucosa at biopsy sites further up the colon and avoid confounding results caused by a the small amount of KY-like lubricant routinely used during the endoscopy procedure.

- Histopathology

Rationale: Endoscopy will be performed for the sole purpose of obtaining biopsy specimens at baseline, following single exposure, and following one week of QD exposure. Samples will be obtained at each flexible sigmoidoscopy and set aside for batch sectioning and H&E staining. Slides will be reviewed and scored by a qualified pathologist using the qualitative scoring system (see Appendix III) developed for the HPTN 056 study and adapted from validated scoring systems used in inflammatory bowel disease (IBD)[40].

This scoring system is intended to improve upon the coarse scoring system used in past studies. Prior to the HPTN 056 study, one rectal microbicide study using histological data[37], employed a simple scoring system of normal, slightly abnormal, or abnormal. Using this histological system 69% of the placebo recipients and 89%of the N-9 recipients had slightly abnormal or abnormal rectal biopsies. The scoring system developed for the HPTN 056 study might provide better discrimination between abnormal and normal histology.

These indices are research measures only and will not be available or used in real-time to make clinical decisions, but will be analyzed as a secondary endpoint. In the immunologically responsive rectum, histologic assessment alone may be inadequate to evaluate subclinical cellular alterations in mucosal integrity. However, histology will inform in part the safety profile of the study products.

Histology is preferable to visual inspection which is a coarse measurement of mucosal health for several reasons 1) the wide spectrum of normal mucosal appearance, 2) inter-observer variability, 3) intra-observer variability[40]. The single-cell rectal lining is easily damaged and quickly repairs. Presence of epithelial cells in effluent is normal; mucosal redness, flushing, loss of vascular pattern and even small erosions may all be normal variants and/or a result of colonic preparations. Should any significant abnormalities be noted during the exam, they will be recorded as adverse experiences (see Appendix IV and V Toxicity Tables), but endoscopic appearance will not be a study endpoint.

- Microflora

Rationale: As with vaginal administration of microbicides, there is concern that rectal administration could disrupt the rectal ecology and, therefore, assessments of pre/post exposure-related changes are indicated. Whether rectal administration of drug product will lead to a shift in rectal microflora and whether these shifts will impact health has yet to be determined.

Simon and Gorbach's extensive review of the literature on intestinal microflora describes the concentrations and composition of the gut microflora[41]. Human bacterial concentrations in the colon range from 10^{11} to 10^{12} cfu/ml, with anaerobic bacteria outnumbering aerobes by 1000-fold. The predominant colonic colonizers are Bacteroides, Bifidobacterium, and Eubacterium. Other common colonic organisms are Lactobacilli, Clostridia, Enterobacter, Clostridia, and anaerobic gram-positive cocci. The microflora populations are relatively stable with few qualitative changes along the colonic wall within subjects and are relatively independent of dietary changes.

While the microbiota has more routinely been assessed by culture-dependent methods, other methods have indicated that up to 60-80% of colonic microflora remain undefined by culture[42]. Several microbicide studies assessing microflora ecology have utilized a semi-quantitative microflora assay of swab samples. Patton and Hillier have developed this assay to examine, macaque and human vaginal microflora, as well as the macaque rectal microflora. Of note, when Patton exposed macaques to UC-781 gel rectally, no significant change in the rectal microflora occurred[43]. In order to similarly assess for any shifts in human rectal microflora after exposure to UC-781, a swab sample will be collected at baseline and after the 7-day outpatient exposure.

- Mucosal mononuclear cell phenotype

Rationale: Co-receptor expression (e.g., CCR5, CXCR4, etc.) on exposed mucosal immunocytes is important for HIV-1 entry. In healthy HIV-1 seronegative individuals, the expression level of CCR5 is increased seven-fold in mucosal mononuclear cells (MMC) compared to peripheral blood mononuclear cells (PBMC)[44]. CXCR4, however, is expressed on CD45RO+ T cells in similar levels in MMC and PBMC. It was recently shown that MMC are more easily infected with HIV-1 than PBMC[45,44]. Explanations for the high susceptibility to HIV-1 of MMC may include the increased expression of HIV-1 co-receptors, especially CCR5, as well as the activation status of the MMC. The expression of CCR5 has been shown to be up-regulated by pro-inflammatory and T helper (Th)-1 cytokines, while Th-2 cytokines up-regulate CXCR4[46,47]. This suggests that expression of CCR5 and CXCR4 is partly controlled by Th1/Th2 type of cytokines, which have been shown to be up-regulated in rectal mucosa from HIV-infected patients[48]. It will be important to ascertain whether microbicidal agents trigger similar responses and associated increased vulnerability to HIV infection. In order to assess co-receptor expression, flow cytometry will be performed on MMC's derived from biopsy specimens.

- Mucosal cytokine Profile

Rationale: RANTES, macrophage inflammatory protein (MIP)-1α and MIP-1β13 are the natural ligands for CCR5 while stromal-derived factor (SDF)-1 is the ligand for CXCR4. The physiological function of β-chemokines and their receptors is to direct migration of recruited lymphocyte subsets to sites of inflammation and immune activation[49,50,51], furthering the inflammatory cascade. Blocking chemokine activity has proved to be effective for inhibiting the migration of certain leukocytes[52] while up-regulation of chemokine receptors and their ligands are characteristic correlates of mucosal

inflammation[53,54]. Immune activation of resting $CD4^+$ T cells has been shown to trigger viral replication and spread[55,56]. In order to assess cytokine expression PCR for mRNA and Luminex® analysis of secreted cytokines from the biopsy specimens will be quantified at baseline and subsequent to study product administration.

- Mucosal immunoglobulins

<u>Rationale</u>: The gastrointestinal mucosa secretes protective mucosal antibodies (mainly IgA and IgG) continuously which, coupled with the physical barrier effects of mucosal mucus, enhance protection. Selective amplification of HIV-specific antibodies has been the goal of most HIV vaccine trials, including the few that have begun to investigate mucosal specific forms. In microbicide trials, changes from baseline levels of total mucosal Ig (or IgA/IgG subsets) may be used to evaluate potential hypersensitivity or immune compromise induced by the study products [57]. Mucosal secretions will be collected using surgical sponges applied topically. These secretions will then be analyzed for IgA/IgG titers.

- Fecal calprotectin

<u>Rationale</u>: Calprotectin accounts for 60% of the cytoplasmic protein fraction of polymorphonuclear granulocytes and is also found in monocytes, macrophages, and eosinophils[58,59]. Calprotectin plays an important role in innate immunity and has antibacterial, antifungal, and immunomodulatory effects in vivo.

Because intestinal granulocytes end their lifespan by migration through the intestinal wall and granulocyte-derived calprotectin can be found in feces, calprotectin is felt to be a useful indirect index of mucosal inflammation[60,61]. In fact, fecal calprotectin levels are elevated in inflammatory bowel disease[62] and correlate well with disease activity in Crohn's disease and ulcerative colitis[63,64]. In addition, fecal calprotectin levels have been found to be significantly elevated in first-degree relatives of patients with Crohn's disease even though all the relatives were clinically asymptomatic[65]. These data suggest that the fecal calprotectin assay may be sufficiently sensitive to respond to subtle increases in mucosal inflammation. Fecal calprotectin has a sensitivity of 96% in discriminating between healthy controls (2mg/l; 95% CI 2-3 mg/l) and subjects with active inflammatory bowel disease (91 mg/l; 95% CI 59-105 mg/l)[64]. Stool samples will be collected at the time of rectal lavage for the measurement of fecal calprotectin.

- Explants- Mucosal cytokines and susceptibility to HIV infection

<u>Rationale</u>: Direct HIV challenge of colonic mucosa with HIV *ex vivo* may evolve to be the most relevant predictor of future microbicidal effectiveness[27]. In these experiments, colorectal tissue samples (measuring 8 mm x 2 mm x 1 mm each) acquired before and after mucosal UC-781 exposure will be exposed to standardized infective doses of HIV to determine whether the *in vivo* exposure provided a mechanism for reducing infectivity. Peripheral blood will also be drawn for potential comparisons. This has been previously shown to be a useful adjunct in the development of vaginal microbicides[66,67,68]. This will be one of the first uses of this now published assay in rectal microbicide development[69,70].

2.1.3 Tertiary Objective

To determine the pharmacokinetics of UC-781 vaginal microbicide gel administered rectally in a subset of participants.

Parameters to be studied include:

- UC-781 blood levels to determine absorption from the GI tract

Rationale: UC-781 blood levels will provide some indication of the likelihood of developing resistance to this important class of HIV drugs prior to future trials that may include HIV positive participants.

2.2 Study Design

This is a phase I randomized, blinded, placebo-controlled study of the safety and acceptability of the vaginal formulation of UC-781 gel, when administered rectally, to be conducted in 36 adults (12 per group) residing in the Los Angeles, CA area. All of the participants will be HIV negative. The study design is summarized in the Schema above and the Schedule of Events in Appendix I.

This study will involve 2 stages of treatment. Each treatment stage is independent of the other. **This study aims to examine the effects of 2 different dosing regimens, NOT cumulative toxicity over both stages.** Participants will undergo extensive evaluation during 5 study visits over an approximately 7-14 week period. In addition, acceptability assessments will be completed at baseline and Visit 5.

Interested participants will enter a screening period, which includes (Visit 1) and if necessary a medication washout period. Subjects, who are deemed eligible after this screening period, will return for a baseline evaluation (Visit 2), including clinical evaluation and mucosal specimen collection, as well as a baseline behavioral assessment (BBQ). The BBQ questionnaire will be administered by CASI. Subjects may complete the web-based CASI in the privacy of their own home. If a subject does not have access to the internet, prefers to complete the BBQ at the study site, or requires assistance in using the CASI, they will be accommodated at the study site.

After completing Visits 1 and 2, eligible subjects will be randomized to one of three groups: 0.1% UC-781 gel, 0.25% UC-781 gel, or a placebo gel (12 per group). A subset of 9 subjects (3 from each group) will also participate in a pK sub-study, which will include 6 timepoints (Visit 3- pre, 0.25 hr, 2 hr, 4 hr, Visit 3A- 24 hr, and Visit 5- 1 day post-QD dose draw). During randomization, study staff will be notified if the participant is randomized to the pK sub-study. Subjects will be randomized in blocks of 3, one per group to minimize imbalance in treatment assignments. The first three subjects enrolled in each of three treatment groups will be selected for the pK substudy. It is possible that participants may refuse to participate in the substudy, and spots will continue to be offered as needed.

Once randomized, subjects will return to the clinic (Visit 3), where a single dose of the study gel (Treatment Stage 1) will be administered after a preparatory Normosol-R pH7.4 enema. Within approximately 30 minutes, mucosal specimens will be obtained, including post-dose lavage, sponge specimens and colorectal biopsies. Subjects will receive a safety follow-up phone call.

Subjects will return to the clinic for Treatment Stage 2 Clearance (Visit 4), in the week prior to beginning Treatment Stage 2. If given clearance, the subject may begin Treatment Stage 2.

In Treatment Stage 2, subjects will begin to self-administer once daily outpatient doses of the study gel for 7 days. Subjects will receive a follow-up safety phone call mid-way through

Stage 2. After completion of Stage 2 the subject will complete a Product Acceptability Questionnaire (PAQ) by CASI and return to clinic for evaluation and specimen collection (Visit 5). Subjects will receive a safety follow-up phone call.

After completion of Visit 5, subjects will be contacted by an interviewer to complete an in-depth phone interview regarding product acceptability. [The complete results of these interviews will not be shared with the rest of the research staff until the blind has been broken, as subjects may unwittingly reveal information that would break the blind. Only preliminary data that does not compromise the blind may be shared prior to data lockdown and unblinding.]

PRN visits

- Any complications that arise may require further clinical evaluation.

3.0 STUDY POPULATION

Thirty-six HIV negative adults will be selected for the study according to the criteria in Sections 3.1 and 3.2. Participants will be recruited, screened, and enrolled concurrently as described in Section 3.3, 5.1, and 5.2.

Issues related to participant retention and withdrawal from the study are described in Sections 3.4 and 3.5, respectively.

3.1 Inclusion Criteria

Men who meet the following 10 criteria and women who meet the following 12 criteria are eligible for inclusion in the study:

1. ≥ Age of 18
2. HIV-1 status antibody negative as documented at screening
3. Understands and agrees to local STI reporting requirements
4. Able and willing to communicate in English
5. Able and willing to provide written informed consent to take part in the study
6. Able and willing to provide adequate information for locator purposes
7. Availability to return for all study visits, barring unforeseen circumstances
8. A history of consensual RAI at least once in lifetime*

   **Required to assure that subjects have a context for the acceptability assessments.*

9. Willing to abstain from insertion of anything per rectum other than the study gel for the 1 week prior to treatment, 1 week prior each flexible sigmoidoscopy (i.e. during week of study gel use), and 1 week after each flexible sigmoidoscopy.
10. Must agree to use condoms for the duration of the study

In addition to the criteria listed above, female participants must meet the following criteria:

11. Negative pregnancy test

12. Post-menopausal or using an acceptable form of contraception (e.g. barrier method, IUD, hormonal contraception, surgical sterilization, or vasectomization of male partner). If the female subject has female partners only, the method of contraception will be noted as a barrier method in the study documentation.

3.2 Exclusion Criteria

Individuals who meet any of the following criteria at screening will be excluded from the study:

1. HIV positive at baseline

2. History of inflammatory bowel disease

3. Active inflammatory condition of the GI tract at baseline

4. Active rectal infection at baseline

5. ≥ Grade 2 laboratory abnormality at baseline

6. History of an underlying cardiac arrhythmia or renal disease that may be exacerbated by electrolyte abnormalities.

7. History of severe or recent cardiac or pulmonary event

8. History of a large aortic aneurysm

9. History of significant gastrointestinal bleeding

10. Allergy to methylparaben, propylparaben, sorbic acid

11. History of alcoholism or IV drug abuse

12. Unwillingness to refrain from chronic use of aspirin and NSAIDs.

13. Use or warfarin or heparin

14. Use of systemic immunomodulatory medications within 4 weeks of Visit 2

15. Use of rectally administered medications, with the exception of over-the-counter enemas, within 4 weeks of Visit 2

16. Use of product containing nonoxyl-9 rectally within 4 weeks of Visit 2

17. Use of any investigational products within 4 weeks of Visit 2

18. Any other clinical condition or prior therapy that, in the opinion of the investigator, would make the patient unsuitable for the study or unable to comply with the study requirements. Such conditions may include, but are not limited to, current or recent history of severe, progressive, or uncontrolled renal, hepatic, hematological, gastrointestinal, endocrine, pulmonary, neurological, or cerebral disease.

In addition to the criteria listed above, female participants will be excluded if the meet any of the following criteria:

19. Pregnancy

20. Breastfeeding

21. Female of child-bearing potential unwilling to use acceptable form of contraception

3.3 Recruitment Process

Recruitment will occur using four main strategies:

i. Clinician-patient referrals;
ii. Use of existing “study registries” that contain the names and phone numbers of individuals who have given informed consent to be reached for future studies for which they may be eligible.
iii. Participant referrals (participants refer their friends or partners who may meet eligibility criteria); and
iv. Passive self-referral: interested individuals see a study poster or brochure advertising the study and call the study site directly.

Study staff will meet as needed to discuss current recruitment status, targets, and strategies. Staff also will follow-up with all persons who express an interest in the study to ensure that screening appointments are scheduled and carried out in a timely manner.

3.4 Participant Retention

Once a participant enrolls in this study, the study site will make every effort to retain him/her for the duration of follow-up in order to minimize possible bias associated with loss-to-follow-up. The study staff is responsible for developing and implementing local standard operating procedures to target this goal. Components of such procedures include:

- Thorough explanation of the study visit schedule and procedural requirements during the informed consent process, and re-emphasis at each study visit;

- Thorough explanation of the importance of all three treatment phases to the overall success of the study;

- Use of appropriate and timely visit reminder mechanisms (via email and/or telephone); and

- Immediate and multifaceted follow-up on missed visits.

3.5 Participant Withdrawal

Regardless of the participant retention methods just described, participants may voluntarily withdraw from the study for any reason at any time. The Investigator also may withdraw participants from the study in order to protect their safety (e.g. pregnancy, diagnosis of colon cancer, ulcerative colitis) and/or if they are unwilling or unable to comply with required study procedures.

Participants also may be withdrawn if the IRB, study sponsor, government or regulatory authorities terminate the study prior to its planned end date.

Every reasonable effort will be made to complete a final evaluation (as described in Section 5) of participants who terminate from the study prior to the final visit, and study staff will record the reason(s) for all withdrawals from the study in participants' study records. Participants who withdraw or are withdrawn prior to receiving study product will be replaced. Participants who withdraw or are withdrawn after receiving study product will not be replaced.

4.0 STUDY PRODUCT CONSIDERATIONS

4.1 Drug Characteristics

UC-781 is the code name for N-[4-chloro-3-(3-methyl-2-butenyloxy)phenyl]-2-methyl-3-furancarbothioamide. UC-781 is a thiocarboxanilide pentenyloxy ether derivative of the carboxanilide class of compounds. It is a pale yellow powder with a molecular weight of 335.84 AMU and has low miscibility in water.

4.2 Study Product

UC-781 Gel 0.1% is an off-white to light yellow aqueous gel formulation containing 0.1% (w/w) UC-781, Carbomer 974P, methylcellulose, glycerin, purified water and common preservatives (methylparaben and propylparaben). It is adjusted to pH 5.2. The human dose is 3.5 g (approximately 3.5 ml). UC-781 Gel 0.1% is packaged in an overwrapped, single dose, prefilled applicator.

UC-781 Gel 0.25% is a light yellow aqueous gel formulation containing 0.25% (w/w) UC-781, Carbomer 974P, methylcellulose, glycerin, purified water and common preservatives (methylparaben and propylparaben). It is adjusted to pH 5.2. The human dose is 3.5 g (approximately 3.5 ml). UC-781Gel 0.25% is packaged in an overwrapped, single dose, prefilled applicator.

Control Gel is a clear aqueous gel formulation containing non-active substances: sodium chloride, hydroxyethyl cellulose, sorbic acid, sodium hydroxide and purified water. It is adjusted to pH 4.4. The human dose is 3.5 g (approximately 3.5 ml). Control Gel is packaged in an overwrapped, single dose, prefilled applicator.

This control gel has been used in the vaginal UC-781 trials and animal studies. Although the pH of the control gel is not the same as the UC-781 gel, it has been selected for consistency. The normal pH of the rectum is ranges between 6.1-7.5 [71]. However, as a natural protection from secreted bile acids, the rectum secretes bicarbonates contributing to a significant buffering capacity[72]. This buffering capacity should be able to equilibrate small differences in pH between the study products.

4.3 Product Supply and Distribution

Supplies of gel will be shipped to the investigational pharmacy in boxes each containing 8 single-use vaginal applicators. These materials will be prepared under Good Manufacturing Practice (GMP) conditions by Dow Pharmaceutical Sciences, Inc, Petaluma, CA for CONRAD. Product will be shipped to the site under IATA and OSHA standards.

The overwrap for each applicator will be labeled with "Caution: New Drug – Limited by Federal (United States) law to investigational use." A single, blinded label will be affixed to each box. The kit will have an outside two-part, tear-off label. Labels will include the following additional information: randomization number, protocol number, storage conditions and dosing instructions. Only the investigational pharmacy will unblinded to group throughout the study.

Supplies of UC-781 0.1% gel, UC-781 0.25% gel and Control gel are to be stored between 15 - 30°C in a secure area. Subjects will be instructed to store the gel at room temperature and away from direct light. The product has been shown to be stable for 6 months at room temperature, and 6 months at up to 40°C.

4.4 Dose Regimen

Each treatment stage is independent of the other.

Treatment Stage 1- After a preparatory Normosol-R pH7.4 enema, subjects will receive one rectally-administered dose of the study gel approximately 30 minutes prior to flexible sigmoidoscopy. The dose will be delivered via the provided applicator.

Full 7-day recovery period

Up to 3 weeks later:
Treatment Stage 2- Subjects will self-administer one dose of the study gel per rectum QD, preferably at the same time of day, for 7 days.

Study staff will review instructions for dispensing, administering, and storing investigational products with each participant prior to discharge from Visit 4.

4.5 Product Accountability

The investigational pharmacy at the site is required to maintain a complete record of all study products received from the manufacturer and subsequently dispensed and to monitor product expiration dates. All unused study products are to be returned to the study site. At the end of the study, specific instructions will be provided for the return or destruction of the study products by the study site. All drug returned and destroyed will also be recorded on the site accountability logs.

4.6 Adherence Assessment

Participants will be given a Product Use Log at the time product is dispensed. A member of the study staff will complete the header information, which will include the protocol

number and PID copied from the product label. After confirming that the header information is correct and the name on the pharmacy label is the participant's name, the staff member dispensing the study product will sign the log.. The subject will then be asked to complete the log indicating each dose used, the date and any additional comments regarding missed or lost doses. The Product Use Log will be collected at Visit 5 and reviewed by study staff. In addition, on approximately Day 4 of Treatment Stage 2, subjects will receive an interim follow-up call during which adherence will be assessed.

4.6.1 Definition and Management of Non-Adherence

Non-adherence is defined as missing a product dose. Missed doses will not be replaced. Non-adherent participants will be asked their reasons for non-adherence and this information will be recorded on source documents. If the subject discontinues the investigational product due to an AE, the subject will not be considered non-adherent. See Section 6.0 for Safety Monitoring.

4.7 Concomitant Medications

Concomitant medications are defined as medications taken within 4 weeks prior to enrollment (Visit 2) and throughout the study. Concomitant medications will be permitted with the exception of those prohibited under the inclusion/exclusion criteria. All concomitant medications, including dose changes, will be reported in the source documents and case report forms.

5.0 STUDY PROCEDURES

An overview of the study visits and procedures schedule is presented in Appendix I. Participant accrual will take approximately 9-12 months and each participant will be on study for approximately 7-14 weeks. The total duration of the study will be approximately 18 months. Presented below is additional information on visit-specific study procedures. Detailed instructions to guide and standardize all study procedures will be provided in the study-specific procedures manual.
All routine laboratory tests (HIV-1 antibody test, syphilis test, assays for rectal HSV, Chlamydia, and gonorrhea) will be conducted by the CLIA certified UCLA Medical Center Clinical Laboratory or a referral laboratory contracted through the UCLA Medical Center. All laboratory results will be reported to the trial participants once the results are available. Participants who receive unexpected results such as HIV seropositivity and/or rectal infection will be referred to available sources of medical and psychosocial care for appropriate support, counseling, clinical assessment, and treatment.

5.1 Visit 1- Screening Visit (up to 1 month prior to Enrollment Visit)

Written informed consent will be obtained before any screening procedures are initiated. For potential participants who do not meet the study eligibility criteria (e.g. the subject tests positive for HIV), the screening process will be discontinued when ineligibility is determined. Subjects not meeting the eligibility may be re-screened at a later date, if appropriate, as in the case of a required medication washout period. If a participant is not enrolled within 1 month of signing the informed consent form, the screening process must be repeated.

5.1.1 Non-clinical Procedures

- ◎ Obtain informed consent
- ◎ Assign screen ID number
- ◎ Assess eligibility for study based on inclusion/exclusion criteria
- ◎ Collect locator and demographic information
- ◎ Provide HIV pre-test counseling
- ◎ Give subject copies of all consent forms with investigators' contact information
- ◎ Give concomitant medication logs with thorough explanation for use.
- ◎ Review precautions:

  -All participants should refrain from taking medications that may prolong bleeding, such as aspirin-containing products and/or NSAIDS for the 72 hours prior to and following flexible sigmoidoscopy and biopsy. Acetaminophen is permitted.

  -All participants should abstain from insertion of anything per rectum other than the study gel for the 1 week prior to treatment, 1 week prior to each flexible sigmoidoscopy (i.e. during week of study gel use), and 1 week after each flexible sigmoidoscopy.

  -Explain to participant that if in the course of the endoscopy the gastroenterologist sees signs of pathology, such as a polyp, the subject will be given the recommendation to follow-up with their primary care provider for evaluation; i.e. neither polyps nor any other pathology will be removed/treated by the research staff as part of the clinical trial. If the participant does not have a primary care provider, the study staff will provide them with a referral, but the study will not assume the cost of evaluation or treatment. It is recommended that all participants establish a relationship with a primary care provider prior to participation in this study. Participants will be withdrawn from the study pending further evaluation, but may be re-screened after evaluation as appropriate.

5.1.2 Clinical Procedures

- ◎ Medical history, including Review of Systems (ROS)*
  ** Note for female participants this includes notation of the Last Menstrual Period (LMP)*
- ◎ Ascertainment of current medications
- ◎ Basic physical examination, including but not limited to the following systems:
  - Cardiovascular
  - Respiratory
  - Gastrointestinal, including anoscopy
  - Any system indicated by a positive medical history or ROS
- ◎ Collect blood and rectal swab specimens

5.1.3 Laboratory Procedures

- ◎ Safety labs (~10ml of blood)
  - Complete blood count
  - Complete metabolic panel, including magnesium
  - If female of child-bearing potential, Qualitative HCG

- STD Screen (for potential rectal infections only)
  (~10ml of blood)
  HIV-1 serology (See Appendix IV for testing algorithm)
  RPR or VDRL
  (3 anorectal swabs)
  Rectal HSV by culture
  Rectal *Chlamydia trachomatis by culture*
  Rectal *Neisseria gonorrhea by culture*

5.2 Visit 2- Enrollment (Week 0)

5.2.1 Non-clinical Procedures

- Update locator information and remind participant that he or she will receive visit reminders via email and/or telephone.
- Provide test results from screening visit and post-test counseling.
  If any lab abnormalities ≥Grade 2, subject may not proceed with Visit 2. If the investigator feels that the laboratory abnormalities are transient, the lab tests may be repeated within the visit window. If the repeated labs tests are within normal limits, the subject may proceed with Visit 2 at the discretion of the investigator, the DAIDS Medical Officer, the SMC, and the IRB.
- If subject had a positive STI result at Visit 1, the subject will be referred for treatment of that STI. The subject may proceed to enrollment (Visit 2) one week after a CDC-approved (Appendix X) treatment for the STI.
- Collect copy of concomitant medication log
- Review precautions listed in Section 5.1.1.
- Instruct subject in use of CASI for Baseline Behavioral Questionnaire (BBQ).

Eligible participants who are computer literate will be able to respond to the survey either from their homes or using a terminal at one of the researchers' offices.

Participants who are not computer literate will be given a short tutorial on using the computer to answer questions. When the participant feels comfortable, the research assistant will start the CASI program for the participant. The research assistant will remain in the room while the participant answers the questions but will sit so that he does not see the answers being entered. The research assistant will help with computer questions or problems. If a respondent does not understand a question, the research assistant will read the question aloud, referring to a paper copy of the interview and identifying the question by its number.

5.2.2 Clinical Procedures

- Interval medical history*, including AE assessment, will be collected to assure continued healthy status and safety of study interventions.
  ** Note for female participants this includes notation of the LMP.*
- Basic physical examination as defined in section 5.1.2
  (If participant has peri-anal or rectal HSV lesions, referrals for treatment will be provided and Visit 2 will be postponed until outbreak resolved.)
- Collect blood and rectal swab specimens
- Collect rectal sponge specimens
- Administer preparatory Normosol-R pH7.4 enema
- Collect stool specimen

- Perform flexible sigmoidoscopy, including:
  Rectal lavage at approximately 5cm
  14 biopsies at approximately 10cm
  14 biopsies at approximately 30cm
  ** each biopsy measures 8 mm x 2 mm x 1 mm*

5.2.3 Laboratory Procedures

- Safety labs as defined in section 5.1.3
- STD screen as defined in section 5.1.3
- Research panel
  Epithelial sloughing assay (Lavage)
  Histopathology (~1 biopsy)
  Microflora (~1 rectal swab)
  Mucosal mononuclear cell phenotype (~4 biopsies)
  Mucosal cytokine profile (~3 biopsies)
  Immunoglobulin assay (~2 rectal sponges)
  Fecal calprotectin assay (Stool)
  Explants studies (~6 biopsies, ~10ml blood)

5.3 Visit 3- (Week 2 +/- 1 week) Single Dose Administration

5.3.1 Non-clinical Procedures

- Randomize subject and assign participant ID number prior to Visit Day
- Provide test results from Visit 2 and post-test counseling.
  If any lab abnormalities ≥Grade 2, subject may not proceed with Visit 3. If the investigator feels that the laboratory abnormalities are transient, the lab tests may be repeated within the visit window. If the repeated labs tests are within normal limits, the subject may proceed with Visit 3 at the discretion of the investigator, the DAIDS Medical Officer, the SMC, and the IRB.
- If subject had a positive STI result at Visit 2, the subject will be referred for treatment of that STI. The subject may proceed to Treatment Stage 1 (Visit 3) one week after a CDC-approved (Appendix X) treatment for the STI.
- Update locator information and remind participant that he or she will receive visit reminders via email and/or telephone.
- Collect copy of concomitant medication log
- Review precautions listed in Section 5.1.1.

5.3.2 Clinical Procedures

NOTE: During this visit the timepoints listed below are approximate. Every effort will be made to adhere to the timeline, however due to logistics timing will not be exact. However, the sequence of sample collection during the following visits is crucial.

- Interval medical history*, including AE assessment, will be collected to assure continued healthy status and safety of study interventions.
  ** Note for female participants this includes notation of the LMP.*
- Basic physical examination as defined in section 5.1.2
  (If participant has peri-anal or rectal HSV lesions, referrals for treatment will be provided and Visit 3 will be postponed until outbreak resolved.)
- ***If female of child-bearing potential, collect urine for STAT HCG testing***
- Collect blood specimens, including pre-dose pK draw if applicable
- Collect rectal swabs and sponge specimens

- Administer preparatory Normosol-R pH7.4 enema
- Collect stool specimen
- T=0 Administration of single-dose of study gel by study staff
  If female of child-bearing potential, must confirm HCG negative prior to administration.
- T= 15 min pK draw, if applicable
- T= 30 min Collect rectal sponge specimens
  Perform flexible sigmoidoscopy, including:
  Rectal lavage at approximately 5cm
  14 biopsies at approximately 10cm
  14 biopsies at approximately 30cm
  ** each biopsy measures 8 mm x 2 mm x 1 mm*
- T= 2 hr pK draw, if applicable
- T= 4 hr pK draw, if applicable
  Visit 3A- Subject will return to clinic the next day, if in pK sub-study
- T= 24 hr pK draw, if applicable

5.3.3 Laboratory Procedures

- Safety labs as defined in section 5.1.3
- STD screen as defined in section 5.1.3
- Research panel
  Epithelial sloughing assay (Lavage)
  Histopathology (~1 biopsy)
  Mucosal mononuclear cell phenotype (~4 biopsies)
  Mucosal cytokine profile (~3 biopsies)
  Pre- and post-dose Immunoglobulin assay (~4 rectal sponges)
  Fecal calprotectin assay (Stool)
  Explants studies (~6 biopsies, ~10ml blood)
  UC-781 blood level for sub-study participants (~25ml blood)

5.4 Treatment Stage 1- Follow-up Period

- For a full 7 days after the flexible sigmoidoscopy subjects must refrain from insertion of anything per rectum.
- An attempt will be made to contact the subject approximately one day after Visit 3 for a safety follow-up phone call. Potential AEs that arise may require further clinical evaluation.

5.5 Visit 4- (Week 5 +/- 2 weeks) Treatment Stage 2 Clearance

5.5.1 Non-clinical Procedures

- If subject had a positive STI result at Visit 3, the subject will be referred for treatment of that STI. The subject may proceed to (Visit 4) one week after a CDC-approved (Appendix X) treatment for the STI.
- Update locator information and remind participant that he or she will receive visit reminders via email and/or telephone.
- Collect copy of concomitant medication log
- Review precautions listed in Section 5.1.1.
- At this time subjects will be given clearance, if applicable, to start the once daily rectal administration of study product the next day and reminded to complete Product Use Log during Treatment Stage 2.
- Give participant Product Use Log, with instructions for proper use.
- Dispense study product and lubricant with thorough instructions for proper use. Instruction will include self-insertion of an empty applicator while a staff member is available to provide guidance.

5.5.2 Clinical Procedures

- Interval medical history*, including AE assessment, will be collected to assure continued healthy status and safety of study interventions.
  * Note for female participants this includes notation of the LMP.
- Basic physical examination as defined in section 5.1.2
- (If participant has peri-anal or rectal HSV lesions, referrals for treatment will be provided and Visit 3 will be postponed until outbreak resolved.)
- **If female of child-bearing potential, send STAT HCG testing**
- Collect blood, rectal swab specimens, and urine if applicable

5.5.3 Laboratory Procedures

- Qualitative HCG
- STD screen as defined in section 5.1.3

5.6 Treatment Stage 2- Outpatient

Up to 3 weeks after Visit 3:

- Subjects may not begin study product administration, until they have been given clearance by the staff pending clinical labs.
  If any lab abnormalities ≥Grade 2 at Visit 3, subject may not proceed with Treatment Stage 2. If the investigator feels that the laboratory abnormalities are transient, the lab tests may be repeated within the visit window. If the repeated labs tests are within normal limits, the subject may proceed with Treatment Stage 2 at the discretion of the investigator, the DAIDS Medical Officer, the SMC, and the IRB.

- On approximately Day 4 of Treatment Stage 2, an attempt to contact the subject for an interim follow-up phone-call to assess safety, adherence, and troubleshoot any difficulties with administration will be made. Potential AEs that arise may require further clinical evaluation. At this time subjects will also be reminded to complete Product Use Log.

5.7 Visit 5 – (Week 6 +/- 2 weeks) Treatment 2 Follow-up

Although time from Week 0 may vary by several weeks, visit must be scheduled for Day 8 of Treatment Stage 2 (the day following the last dose of study product +/- 1 day).

5.7.1 Non-clinical Procedures

- Update locator information and remind participant that he or she will be contacted to complete an in-depth acceptability interview.
- Collect Product Use Log and unused product.
- Collect copy of concomitant medication log
- Review precautions listed in Section 5.1.1.
- Review instructions for use of CASI to complete Product Acceptability Questionnaire (PAQ).

5.7.2 Clinical Procedures

- Interval medical history*, including AE assessment.
  ** Note for female participants this includes notation of the LMP.*
- Basic physical examination as defined in section 5.1.2

(If participant has positive STI test result and/or peri-anal or rectal HSV lesions, Visit 5 will still be performed for safety evaluation. Every effort will be made to ensure the comfort of the participant make appropriate referrals for treatment.)

- Collect blood and rectal swab specimens
- Collect rectal sponge specimens
- Administer preparatory Normosol-R pH7.4 enema
- Collect stool specimen
- Perform flexible sigmoidoscopy, including:
  - Rectal lavage at approximately 5cm
  - 14 biopsies at approximately 10cm
  - 14 biopsies at approximately 30cm
  - ** each biopsy measures 8 mm x 2 mm x 1 mm*

5.7.3 Laboratory Procedures

- Safety Labs as defined in section 5.1.3
- STD screen as defined in section 5.1.3
- Research panel
  - Epithelial sloughing assay (Lavage)
  - Histopathology (~1 biopsy)
  - Microflora (~1 rectal swab)
  - Mucosal mononuclear cell phenotype (~4 biopsies)
  - Mucosal cytokine profile (~3 biopsies)
  - Immunoglobulin assay (~ 2 rectal sponges)
  - Fecal calprotectin assay (Stool)
  - Explants studies (~6 biopsies, ~10ml blood)
  - UC-781 blood level for sub-study participants (~5ml)

5.8 Treatment Stage 2- Follow-up Period

- For a full 7 days after the flexible sigmoidoscopy subjects must refrain from insertion of anything per rectum.
- An attempt will be made to contact the subject for a safety follow-up phone call approximately one day after Visit 5. Potential AEs that arise may require further clinical evaluation.

5.9 Visit 6- (within 2 weeks of visit 5) In-depth Telephone Interview
Study staff will contact subject to conduct in-depth acceptability interview via telephone (see script in Appendix VI). Interviewers will use a basic script to initiate discussion, but interviews will vary according to participant responses.

6.0 SAFETY MONITORING AND ADVERSE EVENT REPORTING

6.1 Safety Monitoring

The study site Investigators are responsible for continuous close safety monitoring of all study participants, and for alerting the protocol team if unexpected concerns arise. The protocol team will meet via conference call every two to four weeks during the period of study implementation, and additional ad hoc calls will be convened, if required. A decision to convene the SMC may be made at any time that the team agrees than an unacceptable type and/or frequency of AEs has been observed.

In addition, accrual will be suspended and the UCLA SMC will be convened, if **two or more** study participants experience an **AE ≥ Grade 3**. If the SMC feels the AE's may be sufficient to halt the trial, the SMC will be unblinded. The SMC will then analyze the data in an unblinded fashion and make the determination on whether to continue accrual and product use. No other study staff will be unblinded until the study has been terminated and the data locked down.

6.2 Adverse Event Evaluation Criteria

An AE is defined as any untoward medical occurrence in a clinical research participant administered an investigational product and which does not necessarily have a causal relationship with the investigational product.

All gastrointestinal AEs will be defined and graded as described in the 2006 DAIDS Rectal Toxicity Table Addendum (Appendix IV). Any AEs not defined in the DAIDS Rectal Toxicity Table will be defined and graded as described in the DAIDS Toxicity Table (AppendixV). The DAIDS Toxicity Table can be found in Appendix V and on-line at http://rcc.tech-res-intl.com/.

Each adverse event not described above or in the DAIDS Toxicity Table should be graded for severity using the following scale:

- Mild: subject was aware of the adverse event, but she/he was still able to do all activities; no or minimal medical intervention/therapy required.
- Moderate: the subject had to discontinue some activities due to the adverse event; no or minimal medical intervention/therapy required.
- Severe: the subject was incapacitated by the adverse event and unable to perform normal activities; significant medical intervention/therapy required, hospitalization possible.

For pre-existing lab abnormalities and conditions, only changes ≥ one Grade level will be reported as adverse events.

It is the responsibility of the investigator to determine if an adverse event is definitely related, possible related, probably related or, unrelated to the subject's participation the

study. The investigator or designee will assess the relationship of all AEs to the study product based on the DAIDS EAE Reporting Manual, the Investigator's Brochure, and his/her clinical judgment.

"Suspected Adverse Drug Reaction" (SADR) means:
An adverse event that could potentially have a causal relationship to a study agent (definitely, probably, possibly, probably not related or for deaths, pending).

"Unexpected adverse drug experience" means:
Any adverse drug experience in which the specificity or severity is not consistent with the current investigator brochure.

Definitions of relatedness:

**Definitely-**	Adverse event and administration of study agent are related in time **and** a direct association can be demonstrated with the study agent.
**Probably-**	Adverse event and administration of study agent are reasonably related in time **and** the adverse event is more likely explained by the study agent than other causes.
**Possibly-**	Adverse event and administration of study agent are reasonably related in time **and** the adverse event can be explained equally well by causes other than the study agent.
**Probably not-**	Potential relationship between the study agent and adverse event could exist, but is unlikely, **and** the adverse event is most likely explained by causes other than the study agent.
**Not related-**	The adverse event is clearly explained by another cause not related to the study agent (must have documentation to support this determination).

6.3 Adverse Experience Reporting Requirements

Study participants will be provided with a list of danger signs and a 24-hour telephone number. Participants will be instructed to contact the study clinician to report not only danger signs, but also any events they may experience, except for life-threatening events, for which they will be instructed to seek immediate emergency care. Depending on the severity of the event, the clinician will instruct the participant to present to the study site (for more mild events) or to an emergency room (for more serious events) for immediate evaluation. Where feasible and medically appropriate, participants will be encouraged to seek medical care where the study clinician is based, and to request that the clinician be paged or otherwise contacted upon their arrival. With appropriate permission of the participant, records from all non-study medical providers related to such events will be obtained and required data elements will be filed with the source documentation. All participants reporting an AE will be followed clinically, until the AE resolves (returns to baseline), stabilizes or their last study visit. Subjects may be followed for longer periods of time per the discretion of the investigator, DAIDS, and the sponsor.

Pregnancy

Pregnancy will not be considered an Adverse Event for this study. Pregnancy will require the subject to be withdrawn from the study. However, in the event that a subject becomes pregnant during the study every reasonable effort will be made to follow her through pregnancy outcome. Consent for the site to receive a copy of her medical records related to the pregnancy should be obtained.

Anytime a live birth pregnancy is reported, an attempt to collect medical records of the neonate should be made.

Study site staff will document on study CRFs all AEs reported by or observed in enrolled study participants regardless of severity and presumed relationship to the study product and procedures.

Information on all AEs will be included in reports to the US Food and Drug Administration (FDA), and other applicable government and regulatory authorities. Site staff will report information on all AEs and EAEs to their Institutional Review Board (IRB) in accordance with all applicable regulations and local IRB requirements.

6.4 Expedited Adverse Event (EAE) Reporting to DAIDS

The expedited adverse event (EAE) reporting requirements and definitions for this study and the methods for expedited reporting of adverse events (AEs) to the DAIDS Regulatory Compliance Center (RCC) Safety Office are defined in "The Manual for Expedited Reporting of Adverse Events to DAIDS" (DAIDS EAE Manual), **dated May 6, 2004**. The DAIDS EAE Manual is available on the RCC website: http://rcc.tech-res-intl.com/.

AEs reported on an expedited basis must be documented on the DAIDS Expedited Adverse Event Reporting Form (EAE Reporting Form) available on the RCC website: http://rcc.tech-res-intl.com/.

"Expedited adverse event" (EAE) means:
An adverse experience that meets the criteria for expedited reporting to DAIDS.

"Serious adverse drug experience" (SAE) means:
Any adverse drug experience occurring at any dose that results in any of the following outcomes-

- Death (All reported as EAEs)
- a life-threatening adverse drug experience (Only SADRs reported as EAEs)
- inpatient hospitalization or prolongation of existing hospitalization (Only SADRs reported as EAEs)
- persistent or significant disability/incapacity
- congenital anomaly/birth defect (All reported as EAEs, including unintentional fetal loss, per DAIDS)

Important medical events that may not result in death, be life-threatening, or require hospitalization may be considered a serious adverse drug experience if the event is determined to be serious based upon appropriate medical judgment, may jeopardize the subject and/or requires medical or surgical intervention to prevent one of the outcomes listed in this definition.

6.4.1 EAE Reporting Requirements for this Study

EAE Reporting Level
This protocol will use the DAIDS Standard Level of reporting as defined in the DAIDS EAE Manual.

Study Agents for Expedited Reporting to DAIDS
The study agent that must be considered in determining relationships of AEs requiring expedited reporting to DAIDS are UC-781 gel/placebo and the study agent delivery applicators.

Grading Severity of Events
The DAIDS Table for Grading the Severity of Adult and Pediatric Adverse Events (DAIDS AE Grading Table), Version 1.0, December, 2004, must be used and is available on the RCC website at http://rcc.tech-res-intl.com/. In addition to the DAIDS AE Grading Table, there are protocol-specific adverse event grading criteria, which can be found in Section 6.2 and Appendix IV of this protocol.

EAE Reporting Period
AEs must be reported on an expedited basis at the Standard Level during the Protocol-defined EAE Reporting Period, which is:

- The entire study duration for an individual subject (from enrollment until study completion or discontinuation of the subject from study participation for any reason).

After the end of the Protocol-defined EAE Reporting Period stated above, sites must report serious, unexpected, clinical suspected adverse drug reactions if the study site staff becomes aware of the event on a passive basis, i.e., from publicly available information.

6.4.2 Timetable for Reporting

Any adverse events that are serious (whether expected or unexpected) and possibly associated with use of the drug must be immediately reported to CONRAD and DAIDS by telephone (21 CFR 312.64). If there is any question whether the adverse event meets any criteria of severity, it should be reported by telephone to the CONRAD and DAIDS. In addition, a completed Expedited Adverse Event (EAE) Form must be faxed to the RCC Safety Office within 3 business days (by 5 PM EST) after site awareness that the event has occurred at a reportable level and to CONRAD as soon as possible.

CONRAD will notify the FDA by telephone or fax of any unexpected serious adverse events associated with the use of the drug as soon as possible, but no later than 7 calendar days after initial receipt of the information from the investigator. In addition, CONRAD will inform the FDA in writing. The written notification will be made as soon as possible and in no event later than 15 calendar days after initial receipt of the information.

DAIDS and CONRAD will follow all serious adverse events with the cooperation of the investigator.

All serious adverse events will be reported promptly to the Institutional Review Board by the investigator.

Events that are not serious, but are associated with treatment and unexpected must be reported to the Institutional Review Board by the investigator according to local IRB requirements.

7.0 STATISTICAL CONSIDERATIONS

7.1 Review of Study Design

A total of 36 participants randomized to one of 3 treatment arms, each undergoing 2 treatment phases. All subjects will progress through the both phases. In the first treatment phase, subjects will receive a single dose of study product administered rectally in the clinic. In the second treatment phase the subject will self-administer once daily rectally administered outpatient doses for 7 days. After each treatment phase subjects will undergo clinical evaluation and specimen collection.

7.2 Randomization of Treatment Assignments

The randomization codes will be generated by the UCLA MDP Biostatistics Core, using computer-generated random numbers. Randomization will be performed in blocks of six, with each block containing two assignment codes for each of the three treatment groups. These randomization codes can be thus represented as randomly permuted strings of six letters: two A's, two B's, and two C's. Randomization blocks will be generated sequentially allowing for correction of any imbalance due to the withdrawal of a participant prior to receiving any doses of the study agent.

In addition, to randomize subjects for the pK substudy, beginning with the first block of six letters, one "A", one "B", and one "C" will be randomly tagged with a "pK" to denote the subject has been selected for the substudy. Should all subjects consent, the substudy randomization will thus be completed after the first three blocks of 6 subjects. If any subjects refuse substudy participation, this subrandomization will continue as needed until three subjects from each treatment arm have been recruited for the substudy.

Once generated the UCLA investigational pharmacy with primary responsibility for drug dispensing will be responsible for randomizing subjects and maintaining the blind.

7.3 Unblinding

Once the data set has been locked down per the SSP Manual, the investigators may break the blind. See Section 6.1 for unblinding of the SMC in the case of a non-emergent safety review.

7.3.1 Emergency Unblinding Procedures

Subjects may be unblinded for safety or medical treatment purposes. The investigator must, whenever possible, phone the study's designated SMC medical monitor to unblind the code. The medical monitor will notify the investigator and sponsor that a subject was unblinded without revealing what the treatment assignment was. Appropriate documentation must be submitted to the investigator and CONRAD within 3 working days of unblinding. Any subject that is unblinded must be discontinued from the study.

The Emergency Unblinding Code specifying the subject's treatment code is located on the drug label. Only in case of medical emergency, can the investigator break the masking code by scratching off the unmasking cover. This label is affixed to the drug label source.

7.4 Statistical Procedures for Analysis of Safety Data

The frequency of $\geq$ Grade 2 adverse events will be used as the primary measure of safety of UC-781. Because of the relatively small sample size, only large differences between placebo and study drug groups will be detectable. As an example, there is more than a

90% probability that one or more adverse events will be seen in a group of 12 subjects when the true rate of events is 20%. In contrast, when the true event rate is only 1%, there is an 11% chance of observing one or more AE's in a group of 12 subjects. When the adverse event represents an exacerbation of a baseline condition, the adverse event will be reported as the change in grade from baseline. Serious adverse events will be reported as such, regardless of the baseline condition.

7.5 Statistical Procedures for Analysis of Acceptability Data

There will be 36 participants in the study divided in three different experimental conditions (2 gel arms and one placebo arm) with 12 participants each. The quantitative acceptability assessments will consist of one Baseline Behavioral Questionnaire (BBQ) filled in once by all 36 participants, and one Product Acceptability questionnaire (PAQ) filled in once by all participants after Treatment Stage 2. The qualitative in-depth questionnaire will be conducted by phone after visit 5.

In terms of the quantitative data, our main prediction is that after one week of practice in the use of the product, respondents will, on average, report high intentionality to use the product in the future every time they have receptive anal intercourse. Furthermore, participants will report high intentionality to use the product in those occasions when they don't use condoms. High intentionality will be defined as an average score in the upper one third of the Likert scales measuring these intentions. Additionally, we anticipate that, even though many of the individuals who voluntarily enroll in a study like ours will be predisposed to like the product (as measured at baseline by the BBQ Intentions scale), we forecast that the experience of using the gel will on average increase their intentionality after experiencing the product. We do not anticipate differences in intentionality to use the product in the future among the three arms, since the placebo is designed to be quite similar to the UC-781 conditions.

The quantitative data will also provide descriptive statistics of acceptability of the product, the application process, applicator, as well as the degree to which participants were bothered by leakage, soiling, or other problems related to gel use. Descriptive statistics will also help us measure perceived possibility of covert use and willingness to use a higher volume than the one used in the study.

One of the drawbacks of quantitative assessments of microbicide acceptability is that this construct is unlikely to provide a static result that cannot be expected to be valid once and forever. One's willingness to use a product in the future may be affected by a number of interpersonal and contextual circumstances. To tackle this issue, we will rely on measures that are taken close to the end of the study, when participants have had sufficient practice occasions to achieve more stable opinions on what using the product will be like. Furthermore, taking advantage of the relatively small number of participants in the study, we will integrate quantitative and qualitative data closely so as to explore the roles that different factors play in acceptability and the meaning that enema and gel use as HIV-prevention tools ultimately have for the participants. For example, if in the BBQ a participant describes habitually having RAI using only saliva as a lubricant, and this may negatively influence the acceptability of a gel delivered intrarectally, we will be able to explore this issue in detail with the specific participant during in the in-depth interview that will take place after visit 5. This will give us the chance to explore ways to promote or enhance microbicide acceptability in such cases, and also it will elicit some new hypotheses that could be explored in future studies.

The in-depth interviews will be audio-taped, transcribed, and analyzed for content. In the initial stage of this qualitative data processing, two separate investigators will identify categories, themes, and patterns, and develop a coding scheme. Codes generated by the

two investigators will be compared and synthesized to end up with shared coding structure that will constitute the preliminary codebook. This codebook will be refined through further coding and discussion between the coders until consensus is reached. The analysis of coded material will lead to the progressive identification of categories, themes, and patterns. This will involve noting regularities in the setting or people under study and organizing them under a conceptual label. As the analysis progresses, hypotheses will be developed and challenged with the rest of the collected qualitative data. Ultimately, the insights gained from the qualitative data will be integrated with the results of the quantitative assessment with the purpose of creating a full-fledged picture of the different factors affecting acceptability.

7.6 Statistical Procedures for Analysis of Mucosal Damage Parameters

The associations of six sets of mucosal parameters with the use of study product will be examined as a secondary objective. Four of these parameter sets (Histopathology, Cell Phenotype, Cytokine profile, Fecal Calprotectin) are collected three times per patient: at baseline, post-Treatment 1 and post-Treatment 2. The remaining two sets (Epithelial Sloughing and Immunoglobulins) are four times per patient: at baseline, pre- and post-Treatment 1, and post-Treatment 2. For all six parameter panels, the main question of interest is whether the three treatment groups (placebo, low, and high) have systematically different mucosal damage parameters post-Treatment 1 and post-Treatment 2. In particular, we will be assessing whether either of the UC-781 treatment groups have evidence of more mucosal damage than the placebo group. As described below, at least four of the six parameter sets should be stable and sensitive enough that important clinical differences would be statistically detectable.

As subjects will be randomized to the three treatment groups, systematic baseline differences are not expected; however, due to the relatively small study size, it is quite likely that many parameters will be quite different between treatment groups at baseline. All analyses will therefore be conducted both with and without controlling for baseline measurements. For continuous measures, it is well-known[75] that controlling for baseline differences in regression models is more powerful than analyzing changes from baseline.

The basic model for analyzing continuous measures adjusting for baseline differences will be the analysis of covariance (ANCOVA) model regressing the continuous parameter at either post-Treatment 1 or post-Treatment 2 on the continuous baseline value and the categorical treatment group membership (with placebo as the reference group). The estimated regression coefficients for the treatment group will then be directly interpretable as the difference between low vs. placebo and high vs. placebo, post-Treatment 1 or post-Treatment 2 after adjusting for baseline differences. This model without the baseline values reduces to the simple one-way ANOVA model with treatment group as the predictor. For analyzing binary or ordinal measures, we will use the logistic regression version of these models. That is, we will regress the outcome post-Treatment 1 or post-Treatment 2 on group membership and on baseline value (when adjusting for baseline differences).

As we will be analyzing group differences in a large number of parameters, some attempt must be made to account for multiple hypothesis testing. We will address this multiple comparisons problem both informally and formally. Informally, particular group differences significant at the 0.05 level or smaller will be flagged as at least suggestive and those with extremely strong p-values (e.g. 0.001 or less) will be carefully examined. In addition, differences seen consistently for related parameters will carry more weight. Formally, we will apply corrections to the p-values. Of these, the Bonferonni method is the simplest, but also the weakest. We will employ the more powerful stepdown methods[76] implemented in

the MULTTEST procedure in SAS v 9.1 (SAS Institute, Cary, NC).

We will also investigate longitudinal (three or four time points) and/or multivariate (simultaneous examination of multiple measures in a set) modeling techniques. The longitudinal analyses will most likely not be additionally informative, as there are only three time points for most of the parameters. Multivariate techniques are expected to result in potentially substantial power increases. Our previous studies with phenotype and cytokine panels have shown strong correlations between measures as well as good stability over time (i.e. high intrasubject correlations) in steady state for many of the measures. In this setting, for example, factors derived using principal components analysis would likely have stronger association with group membership than any one particular measure in the set. An additional strength of the longitudinal and multivariate approaches is that they provide an alternative solution to the multiple testing problem that arises with examining each timepoint and each parameter individually.

- Epithelial Sloughing

The simplest analysis for the epithelial sloughing measurements will look at the presence or absence of sloughing. Additionally, we may employ the five point ordinal scale of[77,78], which ranges from 0 (no evidence of sloughing) up to 4 (evidence in all four quadrants). For these data, there is baseline measurement will allow screening out of post-Treatment 1 false positives.

- Histopathology

As in the sloughing data, we will begin by examination of a two point (normal versus abnormal) or three point (normal, slightly abnormal, abnormal) scales. Based on initial analyses of the HTPN 056[57] histopathology panel data, we do not expect that quantitative measures will be substantially more informative than simple qualitative readings.

- Mucosal mononuclear cell phenotype

Many of the flow cytometry measurements from the HPTN 056 study are quite stable across time[57,79,80]. Parameters with high intrasubject correlations (RFI measures are log-transformed) include %CD3, %CD4, %CD8, %CD31, CD38RFI on CD4+, CD38RFI on CD8+, %CD38||CD4, and %CD38||CD8 which have intrasubject correlations of between 0.7 and 0.9. Thus, we expect that clinically important differences in these parameters between treatment groups would be found to be statistically significant in our analyses. CCR5-related parameters had moderate stability (intrasubject correlations of around 0.5).

- Mucosal cytokine Profile

The cytokine data in HPTN 056 and earlier work of our group[57] for RANTES, IFN-γ, and IL-10 all showed strong stability (intrasubject correlations between 0.7 or 0.8; analysis is on log-transformed scale for all three of these). Again, this suggests that any clinically meaningful differences between treatment groups are likely to result in small p-values.

- Mucosal immunoglobulins

The immunoglobulin data collected in HPTN 056[57] appears to be moderately stable within subject (intrasubject correlations are between 0.3 and 0.5 for various measures of IgA and IgG).

- Fecal calprotectin

The fecal calprotectin marker has been shown to be extremely sensitive for discriminating several clinical conditions such as active and inactive Crohn's disease[81,82]. The large majority of measurements for inactive subjects were below normal thresholds and nearly all measurements for active subjects were above normal thresholds. For our study, this suggests that this marker will be particularly helpful in detecting evidence of mucosal damage in the treatment groups.

7.7 Statistical Procedures for Microflora Studies

Microflora measures will be graded on a 0 to 4 ordinal scale and recorded at baseline and post-exposure. Depending on the empirical distributions across the points in this scale, statistical procedures will either involve analysis of the actual pre-post differences (ordinal) or dichotomized versions of the pre and post scores (binary). In the ordinal case, we will use one sample and multi-sample signed rank tests to examine whether (1) there is an overall change in microflora levels and (2) whether the treatment groups differ significantly from each other in pre-post change. Similarly, if dichotomization is more appropriate, then the binary baseline and post-exposure data will be analyzed using exact McNemar tests (to examine if levels change significantly pre to post) and Fisher tests (to examine whether the pre and post prevalence levels differ between groups).

7.8 Statistical Procedures for Explant Studies

The main question of interest for the explant studies is whether one or both of the UC-781 treatment groups have reduced infectivity post-treatment. As virus growth varies according to the day of observation, comparisons will be made when exponential virus growth ("soft endpoint") is achieved using an improved statistical method developed for the Microbicide Quality Assurance Program (MQAP-NICHD) [83]. Optical Density (O.D.) data from all p24 assays will be compared to a universal standard curve for O.D. values within a + 95% confidence interval of the plate standards. Differences between experimental groups will be determined using Repeated Measures ANOVA and ANCOVA (adjusting for baseline differences).

7.9 Statistical Procedures for Analysis of Pharmacokinetic Data

Pharmacokinetics of UC-781 will be evaluated after rectal administration. This will include the UC-781 pK profile of the initial dose (Visit 3) and UC-781 1 day post-QD dose level (Visit 5). The analysis will be performed by investigating plasma concentrations of UC-781 and determining the AUC. Possible UC-781 accumulation will be determined by comparing the median UC-781 AUC on Visit 5 to the median AUC on Visit 3. Maximum concentration of drug (C_{max}) and time to reach maximum concentration of drug (T_{max}) will be calculated as part of the analysis as stated above.

7.10 Statistical Procedures for Missing Data

All reasonable efforts will be made to obtain complete data for all patients; however, missing observations will occur due to missed visits, patients lost to follow-up, or noncompliance with the full acceptability assessment or secondary measures. In order to account for this, and to be able to perform intent to treat analysis on the outcomes, multiple imputation[84] will be employed. The imputation will be implemented using the MI procedure in SAS or the IveWARE library[85]. In order to construct these models independently of the data from the current study, imputation model building will be based wherever possible on the data from HPTN 056.

8.0 HUMAN SUBJECTS CONSIDERATIONS

8.1 Ethical Review

This protocol and the template informed consent form will be reviewed and approved by the DAIDS Prevention Science Review Committee with respect to scientific content and compliance with applicable research and human subjects regulations.

The protocol, site-specific informed consent form, participant education and recruitment materials, and other requested documents (and any subsequent modifications) also will be reviewed and approved by the ethical review bodies responsible for oversight of research conducted at the study site.

Subsequent to initial review and approval, the responsible local Institutional Review Board (IRB) will review the protocol at least annually. The Investigator will make safety and progress reports to the IRB at least annually, and within three months of study termination or completion. These reports will include the total number of participants enrolled in the study, the number of participants who completed the study, all changes in the research activity, and all unanticipated problems involving risks to human subjects or others. The study site will submit documentation of continuing review to the DAIDS Protocol Registration Office in accordance with the current DAIDS Protocol Registration Policy and Procedures Manual.

8.2 Informed Consent

It is the Investigator's responsibility to ensure that the Elements of Informed Consent (refer to Federal Regulation 21 CFR 50.25 and ICH GCP 4.8.10) and Health Insurance Portability Accountability Act (HIPAA) guidelines are followed and documented in the source document file. The process for obtaining informed consent from potential subjects should be clearly documented and appropriately filed with in the site's standard operating procedures.

For each subject, including screen failures, the study monitor will ensure that informed consent and HIPAA procedures were followed and properly documented. To document informed consent procedures, it is recommended that all signed original informed consent forms (ICF) are filed in each subject's source document file with source notes denoting the date and time of consent(s), the person(s) involved with the consent process (e.g., study nurse and physician who answered questions), and a statement that the subject was indeed given a copy of the signed and dated ICF to take home.

A note in the source documents must state that the subject underwent the Informed Consent Process prior to the beginning of any study-specific procedures.

Subjects will be given ample time to review the informed consent and notify their male partner of their intention to participate in the study. The subject will be given the informed consent and be scheduled to return to the clinic to complete the consent process.

If, during the trial, an informed consent revision where new information that might affect the subject's willingness to participate (e.g., study drug safety or change in study procedures) is presented, each "active" study subject (defined as subjects in screening or enrolled) must read and sign the revised ICF. If administrative changes (e.g., contact information) are made, it is not necessary to re-consent active subjects. However, it is recommended that a summary of administrative changes be prepared and provided to active subjects. If a subject terminates the study and an ICF revision occurs after their participation has ended, they do NOT need to read

and sign the revised ICF, but must be informed of any serious safety issues that may appear during the course of the trial.

8.3 Risks

RISKS FROM PHLEBOTOMY

Study participants may experience discomfort when undergoing phlebotomy. During phlebotomy, participants may feel dizzy or faint, or develop a bruise, swelling or infection where the needle is inserted. The risks of the blood draws include pain, bruising, fainting, lightheadedness, and on rare occasions, infection.

RISKS FROM FLEXIBLE SIGMOIDOSCOPY WITH BIOPSIES

Flexible sigmoidoscopy is a commonly practiced medical procedure and the endoscopic procedures done in this trial will not involve any unusual risks or discomforts. The risks associated with these procedures include mild discomfort and the feeling of having a "bloated stomach". Endoscopic biopsies are painless and heal quickly within 3 days. On extremely rare occasions, the endoscopic procedure or biopsies may lead to pain, infection (sepsis), bleeding or perforation of the gastrointestinal tract. Perforation occurs approximately once out of every 100,000 procedures. If this extremely rare complication occurs, antibiotics and surgery to repair the tear may be necessary. The impact of the rectal application of UC-781 on the intestinal mucosa is unknown and may increase the risk of these complications.

RISKS OF SEDATION

A flexible sigmoidoscopy is usually preformed without sedation, although the subject may request to have intravenous sedative medication given to control for discomfort. There is a minimal risk of bruising and/or thrombophlebitis at the IV site. Depending upon the sedation used and the amount given the subject may experience temporary drowsiness and/or lightheadedness. If the subject receives sedation, s/he will need to arrange for a ride home. The subject may also feel nauseated and lose his/her appetite for the remainder of the day. Rare, but severe and life-threatening adverse reactions to conscious sedation are aspiration, cardiac arrest, and/or pulmonary arrest.

RISKS FROM STD TESTING

Participants may become embarrassed, worried, or anxious when completing their HIV-related interviews and/or receiving HIV/STD counseling. They also may become worried or anxious while waiting for their HIV test results or after receiving HIV-positive test results. Trained counselors will be available to help participants deal with these feelings. Although the study site will make every effort to protect participant privacy and confidentiality, it is possible that partcipants' involvement in the study could become known to others, and that social harms may result (i.e., because participants could become known as "high risk" for HIV infection). For example, participants could be treated unfairly or discriminated against, or could have problems being accepted by their families and/or communities.

RISKS FROM ENEMAS

The main risk from having an enema is temporary discomfort. A hollow tube about the thickness of a pencil will be used to put approximately 125ml of Normosol-R pH7.4 into the

rectum and flush it out again (a larger volume may be required if the initial volume does not produce results), along with any stool that is there. This may cause a "bloated" or "crampy" feeling. Some air may be pumped into the rectum as well, causing flatulence. The tube is small, but it might cause some anal or rectal discomfort if the subject has any hemorrhoids or other painful conditions. The impact of the rectal application of UC-781 on the intestinal mucosa is unknown and may increase the risk of discomfort.

RISKS FROM RECTAL SPONGE AND SWAB COLLECTION

There is no risk from the rectal sponge and swabs themselves. Subjects may have minor discomfort from the insertion of the anoscope used during the insertion of the rectal sponges/swabs and the lining of the rectum may become irritated. This irritation may last for the remainder of the day. The impact of the rectal application of UC-781 on the intestinal mucosa is unknown and may increase the risk of discomfort.

RISKS FROM ANOSCOPY

While anoscopy is a common medical procedure, it is possible that the subject could experience mild irritation of the lining, discomfort, bleeding, dizziness, or embarrassment. The impact of the rectal application of UC-781 on the intestinal mucosa is unknown and may increase the risk of discomfort and/or bleeding.

RISK FROM THE APPLICATOR

The applicator is small and is made of plastic. It is possible that the subject may feel some discomfort from the applicator. The applicator has been designed for vaginal, not rectal use. To minimize the risk of trauma during applicator insertion, subjects will be given a lubricant to use in conjunction with the applicator and written instructions, as well as an in-person tutorial, on proper use.

RISKS FROM UC-781 OR PLACEBO

Minimal local irritation and little or no systemic adverse effects are expected with the use of UC-781 gel per rectum at the concentration proposed in this study. However, this formulation of UC-781 gel has not been tested rectally in humans previously. Therefore, the risks of treatment are not known and careful monitoring for adverse effects is planned. A summary of AEs reported to date in clinical studies of vaginal UC-781 and related compounds, which may be relevant for the use of the UC-781 rectally is provided in section 1.2.

RISKS OF COMPUTER ASSISTED QUESTIONNAIRE

There may be discomfort or embarrassment related to questions dealing with sexual behaviors and personal habits. If some of the questions upset or make the subject uncomfortable and they may choose not to answer them.

RISKS OF IN-DEPTH PHONE INTERVIEW

There may be discomfort or embarrassment related to questions dealing with sexual behaviors and personal habits. If some of the questions upset or make the subject uncomfortable and they may choose not to answer them. While not anticipated, there is also the potential risk of a violation of privacy and confidentiality, if someone overhears the telephone conversation.

The procedures listed above may involve risks that are currently unforeseeable.

8.4 Benefits

There may be no direct benefits to participants in this study. However, participants and others may benefit in the future from information learned from this study. Specifically, information learned in this study may lead to the development of a safe and effective microbicide that reduces the risk of HIV transmission.

In addition, participants will receive HIV counseling and testing as part of the study screening process, as well as rectal exams. Participants also will be screened for a number of STDs, and provided STD treatment if applicable.

8.5 Incentives

Pending IRB approval, participants will be compensated for their time and effort in this study, and/or be reimbursed for travel to study visits. Site-specific reimbursement amounts will be specified in the study informed consent forms.

8.6 Confidentiality

All study-related information will be stored securely at the study site. All participant information will be stored in locked file cabinets in areas with access limited to study staff. All specimens sent to the research laboratories will be identified by a coded number only to maintain participant confidentiality. All records that contain names or other personal identifiers, such as locator forms and informed consent forms, will be stored in an area with limited access, separately from study records identified by code number. All databases will be secured with password-protected access systems.

Participant's study information will not be released without the written permission of the participant, except as necessary for monitoring by the NIAID and/or its contractors, the site IRB, and US government and regulatory agencies (e.g. FDA).

A Certificate of Confidentiality will be obtained for this study from the US Department of Health and Human Sciences. This certificate protects study staff from being compelled to disclose study-related information by any US Federal, State or local civil, criminal, administrative, legislative or other proceedings. It thus serves to protect the identity and privacy of study participants.

8.7 Communicable Disease Reporting Requirements

Study staff will comply with all applicable local requirements to report communicable diseases identified among study participants to local health authorities. Participants will be made aware of all reporting requirements during the study informed consent process.

8.8 Study Discontinuation

The study also may be discontinued at any time by NIAID, the IRB(s) or US government and regulatory agencies.

9.0 LABORATORY SPECIMENS AND BIOHAZARD CONTAINMENT

9.1 Local Laboratory Specimens

UCLA Clinical Laboratory
Clinical labs will be run by the UCLA clinical laboratory or a referral laboratory contracted by the UCLA clinical laboratory.

- Safety labs
  Complete blood count
  Complete metabolic panel, including magnesium
  If female of child-bearing potential, Qualitative HCG

- STD Screen (for potential rectal infections only)
  HIV-1 serology (See Appendix II for testing algorithm)
  RPR or VDRL
  Rectal HSV
  Rectal *Chlamydia trachomatis*
  Rectal *Neisseria gonorrhea*

UCLA Mucosal Immunology Core Laboratory

- Research panel
  Epithelial sloughing assay
  Histopathology
  Mucosal mononuclear cell phenotype
  Mucosal cytokine profile
  Immunoglobulin assay
  Fecal calprotectin assay
  Explants studies

9.2 Outside Laboratory Specimens

Genelogic Laboratories

- pK Studies

Genova Diagnostics

- Fecal calprotectin assay

University of Pittsburgh

- Rectal microflora assay

The study site will adhere to standards of good laboratory practice and local standard operating procedures for proper collection, processing, labeling, transport, and storage of specimens to the local laboratory and outside laboratories.

9.3 Specimen Storage

Study staff will store all specimens collected in this study at least through the end of the study. Storage of all tissue samples will follow local standard operating procedure to ensure the anonymity and confidentiality of the trial subjects. The specimens will be

destroyed at the end of the study or transferred to Dr. Anton's Mucosal Immunology Core Tissuebank, after all protocol-required and quality assurance testing has been completed.

9.4 Biohazard Containment

As the transmission of blood-borne pathogens can occur through contact with contaminated needles, blood, and blood products, appropriate blood and secretion precautions will be employed by all personnel in the drawing of blood and shipping and handling of all specimens for this study, as currently recommended by the United States Centers for Disease Control and Prevention. All infectious specimens will be transported in accordance with US regulations (42 CFR 72).

10.0 ADMINISTRATIVE PROCEDURES

10.1 Study Activation

Following ethical review and approval, the study site will submit required administrative documentation (as listed in the study-specific procedures manual) to the MDP Regulatory CORE B. CORE staff will work with study site staff and complete "protocol registration" in accordance with DAIDS procedures. Included in this step will be CORE and DAIDS review of the study informed consent form.

Pending successful protocol registration and submission of all required documents, CORE staff will "activate" the site to begin study operations. Study implementation may not be initiated until a study activation notice is provided to the site.

10.2 Study Coordination

Study implementation will be directed by this protocol as well as the SSP manual. The SSP manual will outline procedures for conducting study visits; data and forms processing; safety assessment, management and reporting; and other study operations. The Protocol Team will develop study case report forms and as part of the study activation process, the Investigator will identify all case report forms to be used as source documents.

Close coordination between protocol team members will be necessary to track study progress, respond to queries about proper study implementation, and address other issues in a timely manner. Rates of accrual, adherence, and follow-up will be monitored closely by the team as well as the MDP Executive Committee. The Project Leader, DAIDS Medical Officer, Protocol Biostatistician, and MDP Regulatory CORE will address issues related to study eligibility as needed to assure consistent case management and documentation.

10.3 Study Monitoring

On-site study monitoring will be performed in accordance with DAIDS policies. Study monitors will visit the site to

- verify compliance with human subjects and other research regulations and guidelines;
- assess adherence to the study protocol, study-specific procedures manual, and local counseling practices; and
- confirm the quality and accuracy of information collected at the study site and entered into the study database.

Site investigators will allow study monitors to inspect study facilities and documentation (e.g., informed consent forms, clinic and laboratory records, other source documents, and case report forms), as well as observe the performance of study procedures. Investigators also will allow inspection of all study-related documentation by authorized representatives of the MDP Regulatory CORE, NIAID, and US government and regulatory authorities. A site visit log will be maintained at the study site to document all visits.

10.4 Protocol Compliance

The study will be conducted in full compliance with the protocol. The IRB, CONRAD, and DAIDS must be informed of all protocol deviations during the annual review. All correspondence regarding the protocol deviations will be filed in the regulatory files.

The protocol will not be amended without prior written approval by the MDP Program Director, the Director of the MDP Regulatory CORE, and NIAID Medical Officer. All protocol amendments must be submitted to and approved by the relevant local IRB and the DAIDS Regulatory Compliance Center (RCC) prior to implementing the amendment. CONRAD will be informed of any protocol amendments.

10.5 Investigator's Records

The study site investigator will maintain, and store in a secure manner, complete, accurate, and current study records throughout the study. Study records include administrative documentation — including site registration documents and all reports and correspondence relating to the study — as well as documentation related to each participant screened for and/or enrolled in the study — including informed consent forms, locator forms, case report forms, notations of all contacts with the participant, and all other source documents. Records will be stored according to 21 CFR 312.62, which states, “An investigator shall retain records required to be maintained under this part for a period of 2 years following the date a marketing application is approved for the drug for the indication for which it is being investigated; or if no application is to be filed or if the application is not approved for such indication, until 2 years after the investigation is discontinued and FDA is notified”.

10.6 Use of Information and Publications

Publication of the results of this study will be governed by the MDP Program Director. Any presentation, abstract, or manuscript will be submitted by the Investigator to the CONRAD and DAIDS for review prior to submission.

11.0 INVESTIGATOR AGREEMENT

Investigator Agreement:

I agree to conduct this clinical trial in accordance with the design and specific provisions of this protocol; minor deviations from the protocol will be reported to the sponsor, DAIDS, and the IRB on an annual basis. I agree to conduct the study in full accordance with generally accepted standards of Good Clinical Practice as described in the Declaration of Helsinki and in the Statement of Investigator (Form FDA 1572), which I have also signed. I have read and understand the information in this protocol and in the Investigator's Brochure, including the potential risks and side effects of the product under investigation and will ensure that all colleagues and employees conducting the study are aware of this information and their obligations under this protocol.

I agree to report all information or data in accordance with the protocol and, in particular, to report any serious adverse event experiences. I agree to maintain all study documentation for at least two years from the date of US FDA marketing approval for the study product for the indication in which it was studied. If no marketing approval is filed, or if the application is not approved, the records must be retained for two years after the FDA is notified that the IND is discontinued. Any presentation, abstract, or manuscript will be made available by the investigators to DAIDS and CONRAD (the Sponsor) for review prior to submission.

____ ________________________

Signature of Principal Investigator ***Date***

____

Printed Name of Principal Investigator

REFERENCE LIST

1. Mosher, W. D., Chandra, A., and Jones, J. Sexual behavior and selected health measures: men and women 15-44 years of age, United States, 2002. *Advance data from vital heath statistics* 2005;362:

2. Civic, D. College students' reasons for nonuse of condoms within dating relationships. *J.Sex.Marital.Ther.* 2000;26:95-105.

3. Gross, M., Holte, S. E., Marmor, M., Mwatha, A., Koblin, B. A., and Mayer, K. H. Anal sex among HIV-seronegative women at high risk of HIV exposure. The HIVNET Vaccine Preparedness Study 2 Protocol Team. *J Acquir Immune Defic Syndr* 2000;24:393-398.

4. Erickson, P. I., Bastani, R., Maxwell, A. E., Marcus, A. C., Capell, F. J., and Yan, K. X. Prevalence of anal sex among heterosexuals in California and its relationship to other AIDS risk behaviors. *AIDS.Educ.Prev.* 1995;7:477-493.

5. Koblin, B. A., Chesney, M. A., Husnik, M. J., Bozeman, S., Celum, C. L., Buchbinder, S., Mayer, K., McKirnan, D., Judson, F. N., Huang, Y., and Coates, T. J. High-risk behaviors among men who have sex with men in 6 US cities: baseline data from the EXPLORE Study. *Am.J.Public.Health.* 2003;93:926-932.

6. Shelton, A. A. and Welton, M. L. The pelvic floor in health and disease. *West J Med* 1997;167:90-98.

7. McKenna, K. E. The neurophysiology of female sexual function. *World Journal of Urology* 2002;20:93-100.

8. Shafik, A., Shafik, I., and El-Sibai, O. Effect of vaginal distension on anotrectal function: identification of the vagino-anorectal reflex. *Acta Obstet Gynecol Scand* 2005;84:225-229.

9. Janig, W. and McLachlan, E. M. Organization of lumbar sacral outflow to distal colon and pelvic organs. *Physiological Reviews* 1987;67:1332-1404.

10. Pezzone, M. A., Liang, R., and Fraser, M. O. A model of neural cross-talk and irritation in the pelvis: implications for the overlap of chronic pelvic pain disorders. *Gastroenterology* 2005;128:1953-1964.

11. Laumann, E. O., Gagnon, J., and Michael, R. T. The social organization of sexuality: sexual practices in the United States. *The University of Chicago Press* 1994;

12. Catania, J., Canchola, J., Binson, D., Dolcini, M. M., Paul, J. P., Fisher, L, Choy, K., Pollack, L., Chang, J., Yarber, W. L, Heiman, J. R., and Coates, T. J. National trends in condom use among at-risk heterosexuals in the United States. *JAIDS* 2001;27:176-182.

13. Osmond, D. H., Catania, J., Pollack, L., Canchola, J., Jaffe, D., MacKellar, D. A., and Valleroy, L. A. Obtaining HIV test results with a home collection test kit in a community telephone sample. *J Acquir Immune Defic Syndr* 2000;24:363-368.

14. Siegel, K., Krauss, B. J., and Karus, D. Reporting recent sexual practices: gay men's disclosure of HIV risk by questionnaire and interview. *Arch.Sex Behav.* 1994;23:217-230.

15. Rogers, S. M., Willis, G., Al-Tayyib, A., Villarroel, M. A., Turner, C. F., Ganapathi, L., Zenilman, J., and Jadack, R. Audio computer assisted interviewing to measure HIV risk behaviours in a clinic population. *Sex Transm.Infect* 2005;81:501-507.

16. Tourangeau, R. and Smith, T. W. Asking Sensitive Questions: The Impact of Data Collection Mode, Question Format, and Question Context. *Public Opin Q* 1996;60:275-304.

17. Tourangeau, R., Rasinski, K., Jobe, J. B., Smith, T. W., and Pratt, W. F. Sources of error in a survey of sexual behavior. *J Off Stat* 2006;13:341-365.

18. Turner, C. F., Ku, L., Rogers, S. M., Lindberg, L. D., Pleck, J. H., and Sonenstein, F. L. Adolescent sexual behavior, drug use, and violence: increased reporting with computer survey technology. *Science* 1998;280:867-873.

19. Van Damme, L., Ramjee, G., Alary, M., Vuylsteke, B., Chandeying, V., Rees, H., Sirivongrangson, P., Mukenge-Tshibaka, L., Ettiegne-Traore, V., Uaheowitchai, C., Karim, S. S., Masse, B., Perriens, J., and Laga, M. Effectiveness of COL-1492, a nonoxynol-9 vaginal gel, on HIV-1 transmission in female sex workers: a randomised controlled trial. *Lancet.* 2002;360:971-977.

20. Roehr, B., Gross, M., and Mayer, K. Creating a research and development agenda for rectal microbicides that protect against HIV infection. 2001;

21. O'Leary, A. D. and Sweeney, E. C. Lymphoglandular complexes of the colon: structure and distribution. *Histopathology* 1986;10:267-283.

22. Langman, J. M. and Rowland, R. The number and distribution of lymphoid follicles in the human large intestine. *J Anat.* 1986;149:189-194.

23. Patterson, B. K., Landay, A., Andersson, J., Brown, C., Behbahani, H., Jiyamapa, D., Burki, Z., Stanislawski, D., Czerniewski, M. A., and Garcia, P. Repertoire of chemokine receptor expression in the female genital tract: implications for human immunodeficiency virus transmission. *Am J Pathol* 1998;153:481-490.

24. Balzarini, J., Naesens, L., Verbeken, E., Laga, M., Van Damme, L., Parniak, M., Van Mellaert, L., Anne, J., and De Clercq, E. Preclinical studies on thiocarboxanilide UC-781 as a virucidal agent. *AIDS.* 1998;12:1129-1138.

25. Barnard, J., Borkow, G., and Parniak, M. A. The thiocarboxanilide nonnucleoside UC781 is a tight-binding inhibitor of HIV-1 reverse transcriptase. *Biochemistry.* 1997;36:7786-7792.

26. Balzarini, J., Pelemans, H., Aquaro, S., Perno, C. F., Witvrouw, M., Schols, D., De, Clercq E., and Karlsson, A. Highly favorable antiviral activity and resistance profile of the novel thiocarboxanilide pentenyloxy ether derivatives UC-781 and UC-82 as inhibitors of human immunodeficiency virus type 1 replication. *Mol.Pharmacol.* 1996;50:394-401.

27. Lard-Whiteford, S. L. Recommendations for the Nonclinical Development of Topical Microbicides for Prevention of HIV Transmission: An Update. *J.Acquir.Immune.Defic.Syndr.* 2004;36:541-552.

28. Balzarini, J., De, Clercq E., Carbonez, A., Burt, V., and Kleim, J. P. Long-term exposure of HIV type 1-infected cell cultures to combinations of the novel quinoxaline GW420867X

with lamivudine, abacavir, and a variety of nonnucleoside reverse transcriptase inhibitors. *AIDS Res.Hum.Retroviruses* 2000;16:517-528.

29. Report to the CDC Microbicide Working Group: Laboratory Evaluation of Topical Microbicides for the Prevention of HIV Transmission. 2003;

30. Borkow, G., Barnard, J., Nguyen, T. M., Belmonte, A., Wainberg, M. A., and Parniak, M. A. Chemical barriers to human immunodeficiency virus type 1 (HIV-1) infection: retrovirucidal activity of UC781, a thiocarboxanilide nonnucleoside inhibitor of HIV-1 reverse transcriptase. *J Virol.* 1997;71:3023-3030.

31. Buckheit, R. W., Jr., Snow, M. J., Fliakas-Boltz, V., Kinjerski, T. L., Russell, J. D., Pallansch, L. A., Brouwer, W. G., and Yang, S. S. Highly potent oxathiin carboxanilide derivatives with efficacy against nonnucleoside reverse transcriptase inhibitor-resistant human immunodeficiency virus isolates. *Antimicrob Agents Chemother* 1997;41:831-837.

32. CONRAD. Investigator's Brochure: UC-781. 2006;Version 3.0:

33. Patton, D. L. Personal Communication. 2006;

34. Mauck, C., Rosenberg, Z., and Van Damme, L. Recommendations for the clinical development of topical microbicides: an update. *AIDS* 2001;15:857-868.

35. Phillips, D. M. and Zacharopoulos, V. R. Nonoxynol-9 enhances rectal infection by herpes simplex virus in mice. *Contraception* 1998;57:341-348.

36. Phillips, D. M., Taylor, C. L., Zacharopoulos, V. R., and Maguire, R. A. Nonoxynol-9 causes rapid exfoliation of sheets of rectal epithelium. *Contraception* 2000;62:149-154.

37. Tabet, S. R., Surawicz, C., Horton, S., Paradise, M., Coletti, A. S., Gross, M., Fleming, T. R., Buchbinder, S., Haggitt, R. C., Levine, H., Kelly, C. W., and Celum, C. L. Safety and toxicity of nonoxynol-9 gel as a rectal microbicide. *Sex Transm Infect* 1999;26:564-571.

38. Gross, M., Buchbinder, S. P., Celum, C., Heagerty, P., and Seage, G. R., III. Rectal microbicides for U.S. gay men. Are clinical trials needed? Are they feasible? HIVNET Vaccine Preparedness Study Protocol Team. *Sex Transm.Dis* 1998;25:296-302.

39. Phillips, D. M., Sudol, K. M., Taylor, C. L., Guichard, L., Elsen, R., and Maguire, R. A. Lubricants containing N-9 may enhance rectal transmission of HIV and other STIs. *Contraception* 2004;70:107-110.

40. Geboes, K., Riddell, R., Ost, A., Jensfelt, B., Persson, T., and Lofberg, R. A reproducible grading scale for histological assessment of inflammation in ulcerative colitis. *Gut.* 2000;47:404-409.

41. Simon, G. L. and Gorbach, SL. The human intestinal microflora. *Dig.Dis.Sci.* 1986;31:147S-162S.

42. Nielsen, D. S., Moller, P. L., Rosenfeldt, V., Paerregaard, A., Michaelsen, K. F., and Jakobsen, M. Case study of the distribution of mucosa-associated Bifidobacterium species, Lactobacillus species, and other lactic acid bacteria in the human colon. *Appl.Environ.Microbiol.* 2003;69:7545-7548.

43. Patton, D. L., Sweeney, Y. C., Moncla, B. J., Parniak, M., Rohan, L. C., and Hillier S.L. Preclinical Safety Evaluations of UC-781 in Macaque Studies. *Microbicides 2006* 2006;

44. Anton, P. A., Elliott, J., Poles, M. A., McGowan, I. M., Matud, J., Hultin, L. E., Grovit-Ferbas, K., Mackay, C. R., Chen, I. S. Y., and Giorgi, J. V. Enhanced levels of functional HIV-1 co-receptors on human mucosal T cells demonstrated using intestinal biopsy tissue. *AIDS* 2000;14:1761-1765.

45. Lapenta, C., Boirivant, M., Marini, M., Santini, S. M., Logozzi, M., Viora, M., Belardelli, F., and Fais, S. Human intestinal lamina propria lymphocytes are naturally permissive to HIV-1 infection. *Eur J Immunol* 1999;29:1202-1208.

46. Weissman, D., Dybul, M., Daucher, M. B., Davey, R. T., Jr., Walker, R. E., and Kovacs, J. A. Interleukin-2 up-regulates expression of the human immunodeficiency virus fusion coreceptor CCR5 by CD4+ lymphocytes in vivo. *J Infect Dis* 2000;181:933-938.

47. Patterson, B. K., Czerniewski, M., Andersson, J., Sullivan, Y., Su, F., Jiyamapa, D., Burki, Z., and Landay, A. Regulation of CCR5 and CXCR4 expression by type 1 and type 2 cytokines: CCR5 expression is downregulated by IL-10 in CD4-positive lymphocytes. *Clin Immunol* 1999;91:254-262.

48. Kotler, D. P., Reka, S., and Clayton, F. Intestinal mucosal inflammation associated with human immunodeficiency virus infection. *Dig.Dis Sci.* 1993;38:1119-1127.

49. Raport, C. J., Gosling, J., Schweickart, V. L., Gray, P. W., and Charo, I. F. Molecular cloning and functional characterization of a novel human CC chemokine receptor (CCR5) for RANTES, MIP-1beta, and MIP-1alpha. *J Biol Chem* 1996;271:17161-17166.

50. Furie, M. B. and Randolph, G. J. Chemokines and tissue injury. *Am J Pathol* 1995;146:1287-1301.

51. Springer, T. A. Traffic signals for lymphocyte recirculation and leukocyte emigration: the multistep paradigm. *Cell* 1994;76:301-314.

52. Gonzalo, J. A., Lloyd, C. M., Wen, D., Albar, J. P., Wells, T. N., Proudfoot, A., Martinez, A., Dorf, M., Bjerke, T., Coyle, A. J., and Gutierrez-Ramos, J. C. The coordinated action of CC chemokines in the lung orchestrates allergic inflammation and airway hyperresponsiveness. *J Exp Med* 1998;188:157-167.

53. Luster, A. D. Chemokines--chemotactic cytokines that mediate inflammation. *N Engl J Med* 1998;338:436-445.

54. MacDermott, R. P., Sanderson, I. R., and Reinecker, H. C. The central role of chemokines (chemotactic cytokines) in the immunopathogenesis of ulcerative colitis and Crohn's disease. *Inflammatory Bowel Diseases* 1998;4:54-67.

55. Nabel, G. and Baltimore, D. An inducible transcription factor activates expression of human immunodeficiency virus in T cells. *Nature* 1987;326:711-713.

56. Pantaleo, G. and Fauci, A. S. Immunopathogenesis of HIV infection. *Annu.Rev.Microbiol.* 1996;50:825-854.

57. McGowan, I. M., Elliott, J., Andrews, K., Adler, A., Cho, D., and Anton, P. A. HPTN-056: Characterization of Baseline Mucosal Indices of Injury and Inflammation in Men for Use in Rectal Microbicide (RM) Trials. *Microbicides 2006*. Cape Town, SA. 2006;

58. Johne, B., Fagerhol, M. K., Lyberg, T., Prydz, H., Brandtzaeg, P., Naess-Andresen, C. F., and Dale, I. Functional and clinical aspects of the myelomonocyte protein calprotectin. *Mol.Pathol.* 1997;50:113-123.

59. Bjerke, K., Halstensen, T. S., Jahnsen, F., Pulford, K., and Brandtzaeg, P. Distribution of macrophages and granulocytes expressing L1 protein (calprotectin) in human Peyer's patches compared with normal ileal lamina propria and mesenteric lymph nodes. *Gut.* 1993;34:1357-1363.

60. Brun, J. G., Ulvestad, E., Fagerhol, M. K., and Jonsson, R. Effects of human calprotectin (L1) on in vitro immunoglobulin synthesis. *Scand.J.Immunol.* 1994;40:675-680.

61. Yui, S., Mikami, M., and Yamazaki, M. Induction of apoptotic cell death in mouse lymphoma and human leukemia cell lines by a calcium-binding protein complex, calprotectin, derived from inflammatory peritoneal exudate cells. *J.Leukoc.Biol.* 1995;58:650-658.

62. Tibble, J. A., Sigthorsson, G., Foster, R., Forgacs, I., and Bjarnason, I. Use of surrogate markers of inflammation and Rome criteria to distinguish organic from nonorganic intestinal disease. *Gastroenterology* 2002;123:450-460.

63. Roseth, A. G., Aadland, E., Jahnsen, J., and Raknerud, N. Assessment of disease activity in ulcerative colitis by faecal calprotectin, a novel granulocyte marker protein. *Digestion.* 1997;58:176-180.

64. Tibble, J., Teahon, K., Thjodleifsson, B., Roseth, A., Sigthorsson, G., Bridger, S., Foster, R., Sherwood, R., Fagerhol, M., and Bjarnason, I. A simple method for assessing intestinal inflammation in Crohn's disease. *Gut.* 2000;47:506-513.

65. Thjodleifsson, B., Sigthorsson, G., Cariglia, N., Reynisdottir, I., Gudbjartsson, D. F., Kristjansson, K., Meddings, J. B., Gudnason, V., Wandall, J. H., Andersen, L. P., Sherwood, R., Kjeld, M., Oddsson, E., Gudjonsson, H., and Bjarnason, I. Subclinical intestinal inflammation: an inherited abnormality in Crohn's disease relatives? *Gastroenterology.* 2003;124:1728-1737.

66. Shattock, R. J., Griffin, G. E., and Gorodeski, G. I. In vitro models of mucosal HIV transmission. *Nat.Med.* 2000;6:607-608.

67. Greenhead, P., Hayes, P., Watts, P. S., Laing, K. G., Griffin, G. E., and Shattock, R. J. Parameters of human immunodeficiency virus infection of human cervical tissue and inhibition by vaginal virucides. *J Virol.* 2000;74:5577-5586.

68. Fletcher, P., Kiselyeva, Y., Wallace, G., Romano, J., Griffin, G., Margolis, L., and Shattock, R. The nonnucleoside reverse transcriptase inhibitor UC-781 inhibits human immunodeficiency virus type 1 infection of human cervical tissue and dissemination by migratory cells. *J Virol.* 2005;79:11179-11186.

69. Watts, P, Elliott, J., McGowan, I., Anton, P, Griffin, G. E., and Shattock RJ. A human colorectal explant model for HIV-1 infection and microbicide evaluation. *The 11th Confernce on Retroviruses and Opportunistic Infections* 2004;

70. Abner, S. R., Guenthner, P. C., Guarner, J., Hancock, K. A., Cummins, J. E., Jr., Fink, A., Gilmore, G. T., Staley, C., Ward, A., Ali, O., Binderow, S., Cohen, S., Grohskopf, L. A., Paxton, L., Hart, C. E., and Dezzutti, C. S. A Human Colorectal Explant Culture to

Evaluate Topical Microbicides for the Prevention of HIV Infection. *J Infect Dis* 2005;192:1545-1556.

71. Nugent, S. G., Kumar, D., Rampton, D. S., and Evans, D. F. Intestinal luminal pH in inflammatory bowel disease: possible determinants and implications for therapy with aminosalicylates and other drugs. *Gut* 2001;48:571-577.

72. Bottger, W. M., Schoonen, B. J., Moolenaar, F., Visser, J., and Meijer, D. K. A study on the buffering activity of the human rectum. In vivo demonstration of. *Pharm.Weekbl.Sci.* 1989;11:9-12.

73. Bloom, S. Diarrhoea. 2002;1st:351-353.

74. Rockall, T. Upper Gastrointestinal Bleeding: Peptic Ulcers. 2002;1st:139-139.

75. Vickers, A. J. The use of percentage change from baseline as an outcome in a controlled trial is statistically inefficient: a simulation study. *BMC.Med.Res.Methodol.* 2001;1:6-

76. Westfall, P. H. and Young, S. S. Resampling-based Multiple Testing. 1993;

77. Patton, D. L., Cosgrove Sweeney, Y. T., Rabe, L. K., and Hillier, S. L. Rectal applications of nonoxynol-9 cause tissue disruption in a monkey model. *Sex.Transm.Dis.* 2002;29:581-587.

78. Patton, D. L., Sweeney, Y. C., Cummings, P. K., Meyn, L., Rabe, L. K., and Hillier, S. L. Safety and efficacy evaluations for vaginal and rectal use of BufferGel in the macaque model. *Sex Transm.Dis.* 2004;31:290-296.

79. Shacklett, B. L., Yang, O. O., Hausner, M. A., Elliot, J., Hultin L., Price, C., Fuerst, M., Matud, J., Hultin, P., Cox, C., Ibarrondo, J., Wong, J. T., Nixon, D. F., Anton, P. A., and Jamieson, B. D. Optimization of methods to assess human mucosal T-cell responses to HIV infection and vaccination. *J Immunol Methods* 2003;279:17-31.

80. Gurney, K. B., Elliott, J., Nassanian, H., Song, C., Soilleux, E., McGowan, I., Anton, P. A., and Lee, B. Binding and transfer of human immunodeficiency virus by DC-SIGN+ cells in human rectal mucosa. *J Virol* 2005;79:5762-5773.

81. Gaya, D. R., Lyon, T. D., Duncan, A., Neilly, J. B., Han, S., Howell, J., Liddell, C., Stanley, A. J., Morris, A. J., and Mackenzie, J. F. Faecal calprotectin in the assessment of Crohn's disease activity. *QJM.* 2005;98:435-441.

82. Langhorst, J., Elsenbruch, S., Mueller, T., Rueffer, A., Spahn, G., Michalsen, A., and Dobos, G. J. Comparison of 4 neutrophil-derived proteins in feces as indicators of disease activity in ulcerative colitis. *Inflamm.Bowel.Dis* 2005;11:1085-1091.

83. Beer, B. E., Doncel, G. F., Krebs, F. C., Shattock, R. J., Fletcher, P. S., Buckheit, R. W., Jr., Watson, K., Dezzutti, C. S., Cummins, J. E., Bromley, E., Richardson-Harman, N., Pallansch, L. A., Lackman-Smith, C., Osterling, C., Mankowski, M., Miller, S. R., Catalone, B. J., Welsh, P. A., Howett, M. K., Wigdahl, B., Turpin, J. A., and Reichelderfer, P. In vitro preclinical testing of nonoxynol-9 as potential anti-human immunodeficiency virus microbicide: a retrospective analysis of results from five laboratories. *Antimicrob Agents Chemother* 2006;50:713-723.

84. Schafer, J. L. Analysis of incomplete multivariate data. 1997;

85. Raghunathan, T., Solenberger, P., and Van Hoewyk, J. IVEware: Imputation and variance estimation software installation instructions and user guide. 2000;
